# Investigating public support for biosecurity measures to mitigate pathogen transmission through the herpetological trade

**DOI:** 10.1371/journal.pone.0262719

**Published:** 2022-01-21

**Authors:** Elizabeth F. Pienaar, Diane J. Episcopio-Sturgeon, Zachary T. Steele

**Affiliations:** 1 Warnell School of Forestry and Natural Resources, University of Georgia, Athens, Georgia, United States of America; 2 Mammal Research Institute, University of Pretoria, Pretoria, Gauteng, South Africa; 3 School of Natural Resources and Environment, University of Florida, Gainesville, Florida, United States of America; 4 Department of Biological Sciences, Old Dominion University, Norfolk, Virginia, United States of America; University of South Dakota, UNITED STATES

## Abstract

The expanding global trade in herpetofauna has contributed to new infectious disease dynamics and pathways that allow for the rapid spread of pathogens geographically. Improved biosecurity is needed to mitigate adverse biodiversity, economic and human health impacts associated with pathogen transmission through the herpetological trade. However, general lack of knowledge of the pathogen transmission risks associated with the global trade in herpetofauna and public opposition to biosecurity measures are critical obstacles to successfully preventing pathogen transmission. In 2019 we administered a survey to 2,007 members of the public in the United States of America to ascertain their support for interventions to prevent the spread of *Batrachochytrium dendrobatidis* (*Bd*), *Batrachochytrium salamandrivorans* (*Bsal*), ranaviruses, and *Salmonella* through the herpetological trade. We presented survey respondents with different potential hazards associated with pathogen transmission through this trade, namely ecological, economic, and human health impacts. We used structural equation models to determine how these different hazards and respondents’ characteristics influenced respondents’ support for quarantine and veterinary observation of herpetofauna imported into the United States, mandatory tests for diseases of concern, and best practices to reduce stress and improve the care of live herpetofauna during transport to the United States. Respondents’ values and their perceived susceptibility and sensitivity to different hazards associated with pathogen transmission were key determinants of their support for biosecurity. Respondents with strong biospheric and altruistic values demonstrated sensitivity to ecological and human health impacts associated with pathogen transmission, whereas respondents with strong egoistic values demonstrated sensitivity to economic impacts. Respondents had limited knowledge of *Bd*, *Bsal* or ranaviruses, the size of the herpetological trade, or how this trade may contribute to pathogen transmission. Improved outreach and education on pathogen transmission through the herpetological trade is required, but it is important that messages are tailored to people with different values to elicit their support for biosecurity.

## Introduction

Pathogens (e.g., viruses, bacteria, fungi) transmitted through the wildlife trade have received increasing attention in the past 15 years due to the H1N1 (swine flu), H5N1 (bird flu), and SARS-CoV-2 (COVID-19) outbreaks [1,2]. Wildlife are targets of, and reservoirs for, pathogens that may infect native species, domestic animals, and humans. Wildlife are considered to be the source of at least 70% of all emerging diseases [3,4]. Increasing global trade in wildlife and environmental change have generated new infectious disease dynamics and pathways that allow for the rapid spread of pathogens geographically and between species, thereby threatening biodiversity and animal and public health [3–6].

In this paper we focus on pathogen transmission through the herpetological trade, which has resulted in severe ecological, economic, and human health consequences [7–9]. The fungal pathogens *Batrachochytrium dendrobatidis* (*Bd*) and *Batrachochytrium salamandrivorans* (*Bsal*) cause chytridiomycosis (‘chytrid’), an emerging infectious disease that is considered the leading infectious disease threat to biodiversity [7,8]. Ranaviruses (family *Iridoviridae*) are widespread, host-unspecific emerging dsDNA viruses that produce systemic infections in amphibians, reptiles, and fish [10,11]. *Salmonella* bacteria are zoonotic pathogens that cause salmonellosis and are transmitted to poultry, livestock, and humans through direct and indirect contact with herpetofauna [9,12]. Transmission of *Bd*, *Bsal*, ranaviruses and *Salmonella* has been exacerbated by the trade in herpetofauna [5,10,13].

*Batrachochytrium dendrobatidis*, *Bsal* and ranaviruses are main drivers of global declines in amphibian populations [7,10,14,15], which is concerning because recent estimates suggest that 41% of amphibian species are threatened globally [16]. *Batrachochytrium dendrobatidis* has infected >500 species from all three orders of Amphibia and has contributed to declines and extinctions of >200 amphibian species globally [10,15]. *Batrachochytrium salamandrivorans* has been detected across salamanders, frogs, and toads, including some of the most widely traded amphibian species [8], and has resulted in mass mortalities of native wild salamander populations [13,15]. Both *Bd* and *Bsal* are extremely difficult to eradicate once they are established in the wild [7,13].

Ranaviruses infect >175 ectothermic vertebrate species across 52 families [14] and are characterized by high infection prevalence and sudden mortality in multiple species [11]. Ranaviruses may cause severe systemic diseases in marine and freshwater fish and have negatively impacted aquaculture industries globally [14]. Ranaviruses infect species that are both economically important (e.g., the North American bullfrog *Rana catesbeiana*) and of conservation concern (e.g., the gopher tortoise *Gopherus polyphemus*) [14]. Interclass transmission of ranaviruses may occur [14]. Humans accidentally disperse ranaviruses through the transportation of contaminated water or soil (e.g., through recreational activities, agricultural grazing, and other anthropogenic disturbance [11]) and by using fishing bait infected with ranaviruses (e.g., *Ambystoma tigrinum* virus infection in barred tiger salamander *Ambystoma mavortium* larvae sold as fishing bait in the southwestern United States [14]).

*Salmonella* is a globally important zoonotic pathogen (typically transmitted through human consumption of *Salmonella*-contaminated food of animal origin), which results in $3.6 billion annually in economic costs [9]. *Salmonella enterica* is the second most frequently reported zoonotic pathogen in the World Organization for Animal Health (OIE) World Animal Health Information System (WAHIS)-Wild interface [3]. *Salmonella* may remain viable for >30 days in most microcosms and colonizes both wild and captive amphibians and reptiles [12,17,18]. The trade in amphibians and reptiles (which are natural reservoirs of *Salmonella* [12,17,18] has contributed to *Salmonella* transmission, with ≥6% of human salmonellosis cases being attributed to direct or indirect contact with reptiles [9,18,19]. Amphibians and reptiles may also contribute to environmental *Salmonella* contamination in agricultural and recreational areas [17]. Although infection usually causes self-limited gastroenteritis in humans, severe illness and death may occur in children, the elderly and immunocompromised adults [9,18,19]. Antimicrobial and multi-drug resistance in *Salmonella* strains carried by pet herpetofauna is a growing public health safety concern, and may result in increased disease severity, longer hospitalizations, and higher economic costs, leading the World Health Organization to include *Salmonella* on its priority list of 12 antibiotic-resistant bacteria [9,12].

The transmission of *Bd*, *Bsal*, ranaviruses and *Salmonella* through the live herpetological trade occurs because herpetofauna are often shipped at high densities [12,20]. Mixing of animals under dense conditions induces stress, especially in hierarchical, territorial, and aggressive animals or animals with largely solitary behaviors in the wild (e.g., Tokay geckos *Gekko gecko* which are imported for the pet trade [12]). Stress associated with captivity and transport results in immunosuppression, increased mutations and exchange of antibiotic resistance among enteric bacteria, increased pathogen prevalence, and increased shedding and transmission of pathogens by captive herpetofauna [12,20]. Studies show that pathogen prevalence and serotype richness in captive herpetofauna may increase in the 6 months following import [12], which suggests that disease risks may increase once animals have been distributed into the domestic trade.

Biosecurity at ports of entry is thus critical to preventing pathogen transmission through the live herpetological trade [5,21]. However, to date, poor application of biosecurity at ports of entry has resulted in rapid pathogen spread through the transport of infected animals into new regions [5,6,10,15]. Border inspections of wildlife imports are typically aimed at seizures of illegal shipments, rather than prevention of pathogen spread [5]. For example, the United States Fish and Wildlife Service (USFWS) primarily assesses the conservation status of imported animals into the United States, the Center for Disease Control (CDC) focuses on health risks associated with non-human primates, African rodents and bats, and the United States Department of Agriculture (USDA) regulates non-domestic hoofstock, birds and mammals that are imported from countries that are positive for reportable diseases [5]. Improved practices to reduce animal stress and pathogen transmission during transport, more rigorous screening of herpetofauna imports for pathogens, and quarantine of imported animals are needed to prevent pathogen pollution through the introduction of novel pathogens, unique genetic strains of existing pathogens, or multi-drug and antibiotic resistant pathogens into new regions [12,20,21]. Once captive herpetofauna are released into the domestic trade it is extremely difficult or impossible for government agencies to prevent pathogen transmission to native wildlife, domestic animals, or humans, owing to jurisdictional boundaries across government agencies and insufficient funding and staff to actively monitor and regulate trade [12,21].

Unfortunately, implementation of biosecurity measures at ports of entry is often highly political, owing to the economic importance of trade and the substantial costs to the state of biosecurity [22,23]. Investment in effective biosecurity relies on an educated public, legislature and business community that recognizes the risks of pathogen transmission through the herpetological trade and is willing to financially and politically support biosecurity [24–26]. However, social sciences research on public support for biosecurity measures to prevent pathogen transmission through the herpetological trade is missing from the literature–a critical gap given the size of the global trade in herpetofauna [3,6]. Our study was designed to help address this research gap. We conducted research in the United States to test how the public’s values, risk perceptions, knowledge of disease transmission, trust in government, and demographic characteristics influenced their support for biosecurity measures to prevent pathogen transmission through imports of live herpetofauna.

Existing research shows that the public’s support for actions to mitigate risks depends on their risk perceptions [25,27]. Although actual risk depends on the true consequences of a hazard and the objective probability of a negative outcome, risk perceptions are subjective judgments that vary across individuals and influence their behavior under uncertainty [24,28,29]. Risk perceptions are commonly measured in terms of severity and susceptibility (perceived likelihood of occurrence) [24,30]. People’s risk perceptions depend on the specific hazards being evaluated (e.g., pathogen transmission associated with the herpetological trade), their familiarity with or knowledge of these risks, their risk sensitivity (i.e., the weight that they place on risk), their attitudes towards the agents generating risk (e.g., the herpetological trade), and their moral concerns about human interference with nature [24,27,28,30,31]. Women and older individuals tend to have higher risk perceptions related to pathogen transmission through the wildlife trade, whereas individuals with children under the age of 18 or a university degree have lower risk perceptions [24]. Prior research suggests that higher risk perceptions associated with pathogen transmission increase support for biosecurity [24,32].

Research also suggests that public support for biosecurity depends on the public’s trust in the government to mitigate disease risks (referred to as social trust) [6,27,29,30,32–35]. Social trust encompasses the public’s willingness to rely on decision-makers and agency staff who are responsible for biosecurity. Social trust plays an important role in public support for government actions when the public lacks the knowledge, ability, or resources to make independent decisions or identify appropriate actions to mitigate risks such as pathogen transmission through the import of herpetofauna [33,36]. The public is likely to express higher levels of social trust if they evaluate agencies’ past performance in managing risk (i.e., perceived competence or ability) positively and if the agency shares their understanding of a problem, the options available to address the problem, and the relative effectiveness of each of these options [29,30,32,33,35,37].

People’s support for improved biosecurity at ports of entry likely also depends on their values. We focus on four core values which underpin people’s behavior: biospheric, altruistic, egoistic, and hedonic values [38–40]. Biospheric values pertain to people’s concern for the environment, altruistic values encompass their concern for other people’s welfare and wellbeing, egoistic values focus on concern for personal resources, power and achievement, and hedonic values relate to pleasure, comfort, and reduced effort [39]. The relative weights that people place on each of these four values influence their environmental self-identity, personal norms, and risk perceptions [31], which in turn determine their behavior [39,40]. Individuals with strong biospheric and altruistic values (self-transcendence values) are more likely to support pro-environmental interventions because they are concerned about conservation and/or how improved environmental quality supports the health and wellbeing of current and future generations [31,39,40]. By contrast, individuals with strong egoistic and hedonic values (self-enhancement values) are less likely to support interventions that require behavior change and funding, which may be uncomfortable or costly to the individual [38–40]. Thus, we would expect that people with strong biospheric or altruistic values would be more likely to support improved biosecurity, whereas individuals with strong egoistic or hedonic values would be less likely to support these interventions.

However, messaging may be used to highlight disease-related risks that would resonate with individuals who have strong egoistic or hedonic values, such as economic costs or loss of recreational activities associated with pathogen transmission. Health concerns related to pathogen transmission suggest that the public may also be responsive to One Health messages and justification for improved biosecurity [24]. One Health emphasizes that human and animal health are interdependent and fundamentally linked to ecosystem health [3]. If the general public perceives that pathogen transmission is exacerbated by human activities (e.g., the trade in herpetofauna) then their support for biosecurity measures is likely to be higher [30]. However, if the general public perceives diseases to be ‘natural’ (i.e., a natural phenomenon) then their support for biosecurity is likely to be lower [30].

**Fig 1:**
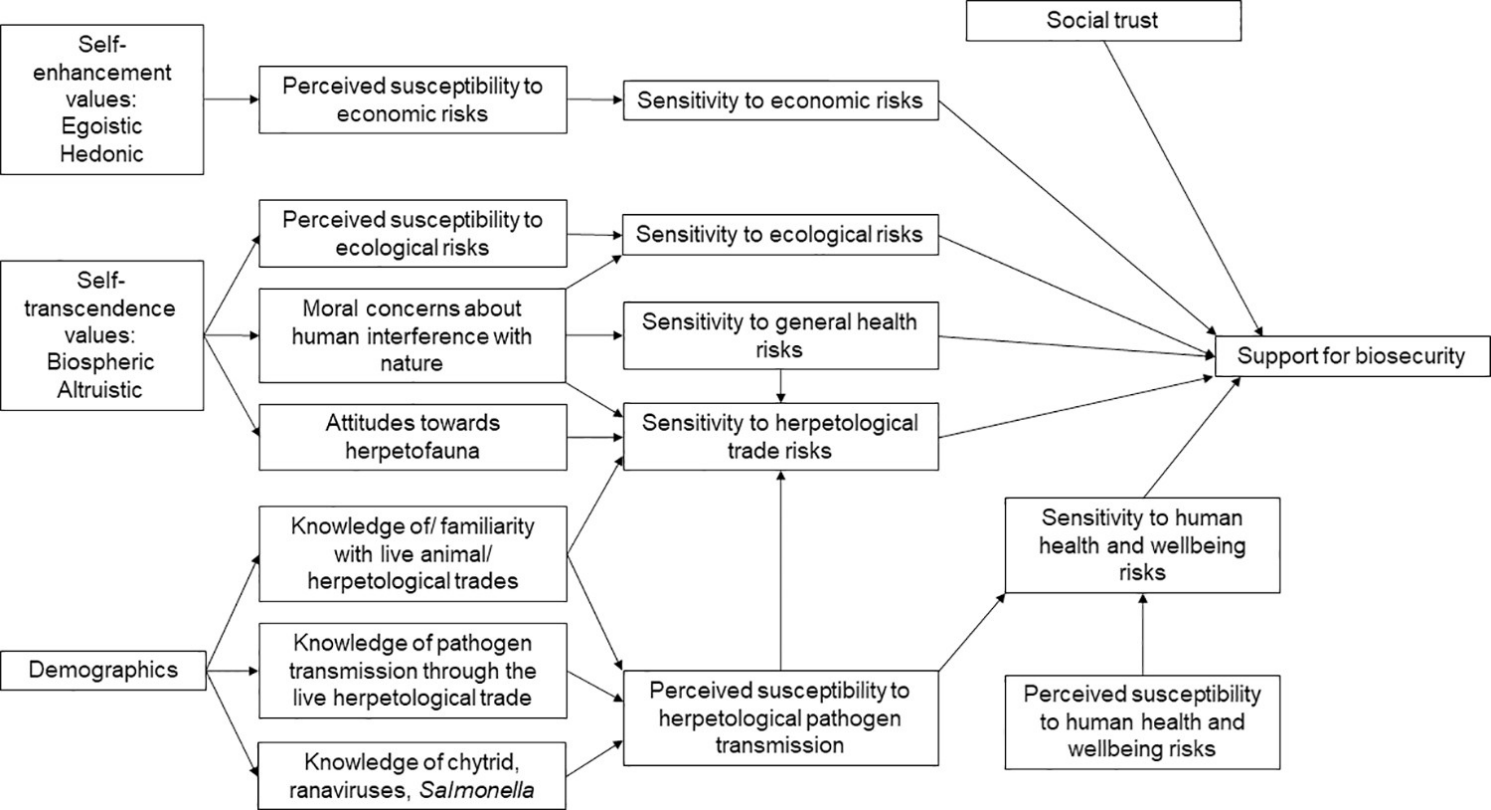
Conceptual model of how respondents’ socio-psychological and demographic characteristics may influence their support for biosecurity measures to prevent the transmission of pathogens into the United States through the importation of herpetofauna.

Based on the existing literature, we predicted that the public’s support for improved welfare of herpetofauna during transport and enhanced biosecurity at ports of entry would be positively correlated with risk perceptions (including prior familiarity with or knowledge of pathogens transmitted by herpetofauna, risk sensitivity, and moral concerns about human interference with nature), perceptions that pathogen transmission is exacerbated by human activities, social trust, and biospheric or altruistic values. We further predicted that the public’s support for improved biosecurity would be negatively correlated with egoistic or hedonic values, or the perception that diseases are a natural phenomenon. Finally, we predicted that demographics and different risks associated with pathogen transmission (ecological, economic, human welfare) would influence public support for biosecurity measures. See Fig 1 for our conceptual framework of how different risks associated with the live herpetological trade and the general public’s values, attitudes, risk perceptions and demographics would influence their support for biosecurity.

## Methods

### Study area

The United States is the largest importer of wildlife globally, with legal imports of 10 to 20 million individual animals each year, which has contributed to the spread of pathogens [6]. It is a main importer of live aquatic animals and herpetofauna [3], largely to supply the pet industry [5]. Based on records from the USFWS Law Enforcement Management Information System (LEMIS), which documents imports and exports of live organisms and wildlife products, between 1999 and 2010, 56 million amphibians and 18 million reptiles were imported into the United States. However, disease surveillance at ports of entry is mandatory for only a small subset of known pathogens transmitted through this trade [6].

We conducted our research in California, Florida, New York, and Texas, four states that play an important role in the trade in live herpetofauna and fish. The USFWS LEMIS dataset showed that in 2015 these four states accounted for the greatest share of amphibian, reptile, and fish imports into the United States (file source: APRIL2015_Lemis_rawdata). Approximately half of all declared wildlife imports are transported through the ports of New York, Los Angeles (California), and Miami (Florida) [5]. Samples of live frogs imported through California and New York demonstrated an infection prevalence of 62% for *Bd* and 8.5% for ranaviruses [41]. Risk models show that the west coast and southeastern United States are at greatest risk of *Bsal* introduction and spread, largely owing to active trade in salamanders and suitable environmental conditions for *Bsal* transmission in wild habitats [8,15]. Although current regulations have reduced the risk of *Bsal* introduction to the United States, incomplete knowledge of which species carry *Bsal* at the time that regulations were implemented has resulted in continued imports of species that may be hosts for the pathogen. Introduction of *Bsal* to the United States could cause an amphibian chytridiomycosis panzootic [13]. Moreover, imports of Indonesian Tokay geckos for the pet trade demonstrated a group prevalence of 31–73% for *Salmonella* [12]. These animals are often released by pet owners owing to their aggressive behavior, which has resulted in established, breeding populations in Florida and Texas, and potential introductions of drug-resistant pathogens from Southeast Asia into new hosts [20]. Reptile-associated salmonellosis accounts for ~5% of all human cases in the United States [12]. During the *Salmonella* outbreak of 2015, California and Texas recorded the highest number of cases of human infection associated with the turtle trade [19].

Our focus on multiple states was also motivated by the fact that it is important when conducting social sciences research on public support for biosecurity to capture regional variations in public attitudes and opinions, thereby improving study rigor [30]. Each of our study states contains large human populations (~39.5 million residents in California, ~21.5 million residents in Florida, ~19.5 million residents in New York, ~29.0 million residents in Texas) who may contribute to pathogen transmission by engaging in the live animal trade (e.g., purchase, transport, or release of diseased pets) or recreational activities (e.g., the use of infected fishing bait), and may differ in their support for biosecurity based on different attitudes and opinions (e.g., regional differences in political views and support for Federal government management of disease risks).

### Sample population

We administered an online questionnaire to adult members of the public from July 17^th^ to September 4^th^, 2019. We paid a company that administers online surveys (Qualtrics) to implement the questionnaire in counties that we selected based on household income and population density. We surveyed both urban and rural residents and attained geographic spread of respondents across each state. We stratified the sample based on income because we posited that higher income individuals might be more likely to contribute to pathogen transmission by engaging in the live herpetological trade, for example by purchasing exotic or rare pets [42]. We selected counties within each state for inclusion in the sample to ensure that we captured the 10^th^, 30^th^, 50^th^, 70^th^ and 90^th^ percentiles of population density and household income across each of the states. We instructed Qualtrics to obtain 500 completed surveys for each state, with equal numbers of survey respondents for each selected county in the state. Based on 2010 Census data, we instructed Qualtrics to recruit respondents who reflected the gender composition (~51% female), age composition (~13% of respondents aged 18–24 years; ~19% aged 25–34 years; ~17% aged 35–44 years; ~17% aged 45–54 years; ~16% aged 55–64 years; ~11% aged 65–74 years; ~7% aged 75 years or older), and race and ethnicity composition of these states (≥11% of respondents who identified as Black or African American; ≥8% who identified as Asian; ≥ 68% who identified as white; ≥32% who identified as Hispanic and/or Latino). We implemented sampling quotas to ensure our final sample was representative of the general public in our study region. Respondents were presented with a written informed consent document before they agreed to participate in the survey. Our study was approved by the University of Florida Institutional Review Board (IRB protocol # 201901788).

### Questionnaire design

#### Pretesting

Prior to finalizing the questionnaire, we thoroughly pretested the survey with 14 experts in the herpetological trade, herpetological diseases, communication, and survey design, and 23 members of the public. Pretests confirmed that members of the public required baseline information to accurately answer questions. We conferred with subject experts in how to effectively present this information without leading or biasing research participants. We used recommended communication techniques (e.g., the use of short, informative sentences and bullet points, the use of graphics and images) to convey information to research participants. We also instructed Qualtrics to measure how long survey respondents spent reading each page of the survey as a means of identifying whether respondents were reading the information provided. Participants who sped through the survey were removed from the final sample and replaced, in order to ensure we collected quality data. A copy of the survey questions is provided in S1 Appendix.

#### Knowledge of the live animal trade

We tested respondents’ prior familiarity with the wildlife trade, with specific focus on the herpetological trade. After informing respondents that wildlife are traded to provide food, bait, skin and fur, to supply the aquaculture, medicine, pet and sport hunting industries, and to provide animals for zoos, research and education [5,43], we asked respondents “How knowledgeable are you about the animal trade?” on a scale of not at all (0) to extremely knowledgeable (10). We then informed respondents that 56 million amphibians, 18 million reptiles, 13.6 million insects and arachnids, 4 million birds and 2 million mammals were imported into the United States between 1999 and 2010 (numbers based on USFWS LEMIS records). We asked respondents “Is the number of live amphibians/reptiles imported into the United States lower or higher than you expected?” (‘much lower than I expected’ = -2, ‘lower than I expected’ = -1, ‘about what I expected’ = 0, ‘higher than I expected’ = 1, ‘much higher than I expected’ = 2). We asked whether respondents were aware that amphibians are imported to the United States for human consumption and use as fishing bait, and that amphibians and reptiles are imported as pets (‘not at all aware’ = 1, ‘slightly aware’ = 2, ‘moderately aware’ = 3, ‘highly aware’ = 4) [5,13,44]. We also asked respondents if they had eaten frog legs (yes = 1, no = 0, I don’t know = 0) or been fishing (yes, no) in the past year, if they had used salamanders as fishing bait (yes, no, I don’t know), and if they knew anyone who owns a pet reptile or amphibian (yes, no).

#### Risk perceptions

We informed respondents that a captive animal is a live animal that is kept or transported and sold for the animal trade and that native wildlife are wild animals that live in an environment where they have been historically found. To measure respondents’ sensitivity to general health risks [24], we asked them to rate how important it was to them to protect the health of animals in the live animal trade, native wildlife, the natural environment, pets, livestock, and humans (‘not at all’ = 1, ‘slightly’ = 2, ‘moderately’ = 3, ‘very’ = 4, ‘extremely’ = 5). These questions captured general health risk sensitivity because we did not frame them in terms of chytridiomycosis, ranavirosis or salmonellosis. We focused on risks to human, domestic animal, wildlife, and ecosystem health because this is consistent with a One Health framework. To capture respondents’ moral concerns about human interference with nature [24], we asked them to indicate whether they agreed with the following statements: “most environmental problems are caused by humans interfering with nature” and “the occurrence of wildlife disease has been made worse by humans and their activities” (‘strongly disagree’ = -2, ‘somewhat disagree’ = -1, ‘neither agree nor disagree’ = 0, ‘somewhat agree’ = 1, ‘strongly agree’ = 2).

To measure knowledge of disease risks, we asked respondents what percentage of captive amphibians and reptiles they thought were healthy. We then informed respondents that although the trade in live amphibians and reptiles is economically important, this trade contributes to pathogen transmission through various pathways: 1) live animals are stressed during transport, which weakens their immune system; 2) inadequate care and nutrition increases the likelihood of pathogen transmission between animals during transport; 3) because animals are housed in high densities they are exposed to pathogens; 4) animals may transmit pathogens to humans through direct contact; and 5) pathogens may also be transmitted by people releasing pets and fish, throwing away unused bait, and throwing out animal products or contaminated materials [9,12–14,18,20,43,45,46]. We measured respondents’ familiarity with these pathogen transmission risks by asking them whether they had read anything or seen any news on pathogen transmission by the live amphibian and reptile trade in the past year (yes, no, I’m not sure).

To assess respondents’ prior knowledge of chytridiomycosis, ranavirosis and salmonellosis, we asked them if they had heard of these diseases prior to the survey (yes = 1, no = 0, I’m not sure = 0). We then explained that chytridiomycosis is a disease that infects amphibians through contact with an infected animal or *Bd* and *Bsal* (which can survive in water or moist areas) [47]. We stated that amphibians absorb oxygen, water, and electrolytes through their skin and that because chytridiomycosis thickens amphibians’ skin they die because they cannot breathe or absorb water and electrolytes [7]. We also informed respondents that ranaviruses affect amphibians, reptiles, and fish by causing fluid build-ups under their skin, blood vessel damage, weakness and difficulty breathing. We explained that ranaviruses transmit across animals through contact with the virus, an infected animal or infected water, and that the virus can survive outside a living host for >30 days [11,14]. Finally, we explained that *Salmonella* is a bacterial pathogen that affects both animals and humans (with humans experiencing headaches, nausea, vomiting, fever, and chills). We stated that reptiles and amphibians are carriers of *Salmonella* and humans can be infected by contact with an infected animal, the animal’s waste, or surfaces that an animal has touched [9,17–19]. After providing information about each disease (and the pathogens that cause these diseases), we measured respondents’ level of concern (‘not at all’ to ‘extremely’) about pathogen transmission from captive herpetofauna (once they have been imported into the United States) to potential, relevant risk targets (other captive animals, native wildlife, pets, livestock, and humans). We measured respondents’ perceptions of risk susceptibility by asking them what they considered the risk of pathogen transmission to be (‘none’ = 1, ‘low’ = 2, ‘moderate’ = 3, ‘high’ = 4, ‘very high’ = 5).

We implemented four different survey versions that focused on different impacts of pathogen transmission (ecological impacts, economic impacts, human health and wellbeing impacts, all impacts) to assess whether different hazards and the perceived severity of risks influenced respondents’ support for improved transport conditions and biosecurity at ports of entry [25]. For all survey versions we explained that if amphibian and reptile species are extirpated then insect populations would increase. For the ecological survey version, we explained that chytridiomycosis currently affects >500 species, with the potential to affect 6,000 species, and has been linked to the decline or extinction of at least 501 amphibian species [7]. We further stated that ranaviruses infect >175 species and are one of the leading causes of death of amphibians in the United States. We explained that the extirpation of amphibians and reptiles would reduce biodiversity and generate trophic cascades, such as the loss of predator species. For the economic survey version, we stated that increased pest populations might result in crop damage, and that aquaculture, the pet trade and the frog leg trade are economically important industries that could be negatively impacted by disease [13]. For the human health survey version, we stated that herpetofauna eat insects that people consider pests (e.g., mosquitoes, flies, beetles, grasshoppers, slugs), and that increased pest populations from a decline in herpetofauna might result in increased prevalence of insect-borne diseases in the human population (e.g., West Nile virus, malaria, Zika virus, Lyme disease). Finally, we stated that 202 people contracted *Salmonella* from turtles from 2015 to 2016, and that although most people recover from salmonellosis without treatment, the disease can be dangerous for children, older adults, and pregnant women [9,17–19]. The ‘all impacts’ survey version presented respondents with the ecological, economic, and human health and wellbeing impacts of pathogen transmission.

After presenting the above information we asked further questions to capture respondents’ risk perceptions pertaining to these different hazards. For the ecological survey version, we asked respondents “How concerned are you about a loss of biodiversity from the disease-related deaths of native amphibians and reptiles?” (‘not at all’ to ‘extremely’; measure of risk sensitivity) and “What do you think the risk is that the diseases discussed in this survey could result in a loss of biodiversity?” (‘none’ to ‘very high’; measure of risk susceptibility). For the economic survey version, we asked respondents “How concerned are you about a negative economic impact to agriculture/aquaculture/the amphibian and reptile pet trade/the frog leg market from disease-related deaths of native amphibians and reptiles?” (‘not at all’ to ‘extremely’; measure of risk sensitivity) and “What do you think the risk is that the diseases discussed in this survey could result in a negative economic impact to agriculture/aquaculture/the amphibian and reptile pet trade/the frog leg market?” (‘none’ to ‘very high’; measure of risk susceptibility). For the human health and wellbeing survey version we asked respondents “How concerned are you about the spread of *Salmonella* from captive amphibians and reptiles to other amphibians and reptiles in the live animal trade/native amphibians and reptiles/pets/livestock/ humans” (‘not at all’ to ‘extremely’). We then asked, “How concerned are you about an increase in insect pests/insect-borne diseases from the disease-related deaths of native amphibians and reptiles?” (‘not at all’ to ‘extremely’; measure of risk sensitivity) and “What do you think the risk is that the diseases discussed in this survey could result in an increase in insect pests/insect-borne diseases?” (‘none’ to ‘very high’; measure of risk susceptibility). The survey version that presented all impacts asked respondents to report their perceived susceptibility and sensitivity to ecological, economic, and human health and wellbeing risks.

#### Biosecurity measures

We presented respondents with three different biosecurity measures to mitigate the pathogen transmission risks associated with imports of herpetofauna: 1) a law requiring the quarantine and veterinary observation of all amphibians and reptiles imported into the United States; 2) mandatory tests of all shipments of amphibians and reptiles for selected diseases of concern; and 3) a mandatory ‘Best Practices Program’ that would require live amphibian and reptile importers and exporters to improve the care and reduce the stress of transported animals and decontaminate all shipping materials [7,13]. Respondents indicated whether they would support or oppose each of these actions (‘strongly oppose’ = 1, ‘slightly oppose’ = 2, ‘neutral’ = 3, ‘slightly favor’ = 4, ‘strongly favor’ = 5).

#### Social trust

Consistent with prior researchers’ [35] definition of trust in management (a core aspect of social trust), we measured respondents’ social trust by asking them their level of agreement (strongly disagree to strongly agree) with five statements that the government has 1) the knowledge, 2) money, and 3) sufficient skilled people to mitigate pathogen transmission through the amphibian and reptile trade, 4) has been effective in mitigating pathogen transmission risks, and 5) can be trusted to mitigate pathogen transmission through the amphibian and reptile trade. We focused on agency competence in our definition of social trust.

#### Respondents’ values

We used the Environmental Portrait Value Questionnaire (E-PVQ), which was adapted from the Schwartz Value Survey [48,49], to measure biospheric, altruistic, egoistic, and hedonic values [39]. Recent research has demonstrated the validity and reliability of the E-PVQ in measuring values that are most relevant to explaining environmental beliefs and behaviors, including environmental self-identity, pro-environmental personal norms, and support for climate change policy [39,40]. We presented respondents with 17 gender matched statements (e.g., “It is important to him to protect the environment”, “It is important to her that every person has equal opportunities”, “It is important to him to enjoy life’s pleasures”, “It is important to her to have authority over others”) and asked respondents to indicate how similar that individual was to them on a 7-point scale from ‘not at all like me’ (1) to ‘very much like me’ (7).

#### Respondent characteristics

We asked respondents whether they liked or disliked amphibians, reptiles, and fish (strongly dislike = -2, dislike = -1, neither like nor dislike = 0, like = 1, strongly like = 2). We also asked respondents whether they owned any pets, livestock, or poultry. Finally, we collected information on respondents’ gender, age, education, race, number of household members <18 years old, and political views (extremely liberal to extremely conservative).

#### Data analysis

We conducted all analysis using STATA/SE version 16. Consistent with our four survey versions, we estimated four structural equation models (SEM) that highlighted the ecological (model 1), economic (model 2), human health and wellbeing (model 3), and all hazards (model 4) associated with pathogen transmission through the herpetological trade. Our prior predictions on how different variables would impact support for biosecurity are captured in Fig 1. We used a two-step approach for structural equation modeling by first testing the measurement models (i.e., confirmatory factor analysis, CFA) that capture how observed variables load on latent factors, and then estimating the structural relationship among latent factors (i.e., structural regression) [25]. We conducted tests for internal consistency and CFA to verify the dimensionality of observed variables that were used to generate scales that measured theoretical constructs (e.g., risk sensitivity, social trust, values). We considered Cronbach’s alpha ≥0.8 to be a good measure of internal consistency [50], although alpha ≥0.7 is adequate [51]. Most observed variables were not normally distributed. Accordingly, we used the asymptotically distribution free estimation method when conducting CFA, which relaxes assumptions of normality and is asymptotically equivalent to maximum likelihood estimation for large samples [50]. We estimated standardized coefficients to identify each variable’s estimated factor loading [25,50], with factor loadings ≥0.5 deemed sufficient for inclusion of the variable in the scale [37]. We considered standardized factor loadings to be significant at the p ≤0.05 level, and a scale to be unidimensional if the comparative fit index (CFI) ≥0.95 and the root mean squared error of approximation (RMSEA) ≤0.05 [50]. We assessed SEM model fit based on the CFI, residual values, and the meaningfulness of the estimated model [33,50]. We considered CFI ≥0.90 and RMSEA ≤0.08 (90% confidence interval of 0–0.10) to be indicative of good model fit for each of the SEM models [25,33,52].

## Results

We collected a total of 2,007 completed surveys (498 from California, 498 from Florida, 507 from New York, 504 from Texas). A total of 1,054 respondents (52.5%) were female (range of 50.2% for Texas to 55.2% for New York; S1 Table). The median age range for respondents was 45–54 years (median of 45–54 years for California, Florida, and New York; median of 35–44 years for Texas; S1 Table). The median education level for respondents was some college or an associate or technical degree for respondents from each state (S1 Table). A total of 1,585 respondents (79.0%) described themselves as white (range of 69.7% for California to 85.1% for Florida) and 463 respondents (23.1%) stated that they were Hispanic and/or Latino (range of 14.8% for New York to 37.5% for Texas; S1 Table). On average, respondents described their political views as moderate (4.12±1.60 where extremely liberal = 1, moderate = 4, and extremely conservative = 7; 3.86±1.65 for California; 4.33±1.59 for Florida; 3.88±1.54 for New York; 4.40±1.54 for Texas). In total, 607 respondents (30.2%) stated that their household contains members <18 years old (range of 27.1% for Florida to 37.5% for Texas; S1 Table). Most respondents (n = 1,338, 66.7%) owned pets, primarily dogs and/or cats (S1 Table). Only a small share of respondents owned fish (n = 142, 7.1%), reptiles (n = 64, 3.2%), or amphibians (n = 10, 0.5%; S1 Table). Similarly, only a small share of respondents owned livestock (n = 43, 2.1%), poultry (n = 39, 1.9%), or both livestock and poultry (n = 47, 2.3%; S1 Table).

### Support for biosecurity measures

Most respondents strongly supported a law that requires the quarantine and veterinary observation of all imported herpetofauna (n = 1,120, 55.8%), mandatory tests of all shipments of herpetofauna for diseases of concern (n = 1,293, 64.4%), and a mandatory ‘Best Practices Program’ to improve the care and reduce the stress of transported animals and decontaminate all shipping materials (n = 1,399, 69.7%; Table 1). Cronbach’s alpha (0.828–0.852 depending on the survey version) confirmed that the items used to measure respondents’ support for biosecurity measures represented a single scale (standardized coefficients>0.7, p<0.001; S2 Table).

**Table 1:** Respondents’ support for management actions to mitigate the disease transmission risks associated with the live herpetological trade (n = 2,007).

	Median	Percent of respondents				
		Strongly oppose	Slightly oppose	Neutral	Slightly support	Strongly support
A law that requires the quarantine and veterinary observation of all amphibians and reptiles imported into the United States	Strongly support	1.6	2.0	11.8	28.8	55.8
Mandatory tests of all shipments of amphibians and reptiles for selected diseases of concern	Strongly support	1.6	1.6	7.9	24.5	64.4
A mandatory ‘Best Practices Program’ requiring live amphibian and reptile importers and exporters to improve the care and reduce the stress of transported animals and decontaminate all shipping materials	Strongly support	0.8	1.0	6.9	21.5	69.7

### Knowledge of the live animal trade

On average, respondents indicated that they were moderately knowledgeable about the live animal trade (median = 5, 4.41±2.71, range: 0–10, n = 2,007). Most respondents indicated that the number of live amphibians and reptiles imported into the United States was higher or much higher than they expected (82.2%, median = ‘higher than I expected’). Few respondents were highly aware that live frogs are imported for human consumption (6.9%), amphibians are imported for use as fishing bait (8.0%), and both amphibians and reptiles are imported to supply the pet industry (15.6%; S3 Table). These three items used to measure respondents’ awareness of the reasons for live amphibian and reptile imports (hereafter, ‘knowledge of herpetological imports’) represented a single scale (Cronbach’s alpha≥0.704; standardized coefficients≥0.56, p<0.001; S4 Table). In total, 118 respondents (5.9%) had eaten frog legs and 505 respondents (25.2%) had been fishing in the past year, of which only 34 respondents (1.7%) had used salamanders as fishing bait. A total of 528 respondents (26.3%) knew someone who owns a pet reptile, 64 respondents (3.2%) knew someone who owns a pet amphibian, and 225 respondents (11.2%) knew someone who owns both a pet reptile and a pet amphibian.

### Attitudes towards herpetofauna and fish

On average, respondents disliked snakes, liked turtles, tortoises, freshwater fish, and saltwater fish, and neither liked nor disliked lizards or amphibians (frogs, toads, salamanders, newts, n = 2,007; S5 Table). Respondents’ ‘attitudes towards herpetofauna’ represented a single scale (Cronbach’s alpha≥0.871, RMSEA≤0.042, CFI≥0.973 for the different survey versions). Whether respondents liked or disliked freshwater and saltwater fish were excluded from the scale (standardized coefficients<0.5). All remaining six indicators of attitudes were significant at the p≤0.001 level with standardized coefficients≥0.57 (S6 Table).

### Risk perceptions

Most respondents considered it very or extremely important to protect the health of animals in the live animal trade, native wildlife, the natural environment, pets, livestock, and humans (n = 2,007; S7 Table). Both Cronbach’s alpha (≥0.886) and CFA (RMSEA≤0.05, CFI≥0.954) confirmed that these items represented a single measure of ‘sensitivity to general health risks’ for each survey version (standardized coefficients≥0.68, p<0.001; S8 Table). Respondents’ perceptions of the importance of protecting the health of humans was excluded from the scale, based on standardized factor loadings and included as a separate variable in the SEM models.

When asked about their moral concerns about human interference with nature, most respondents agreed that “most environmental problems are caused by humans interfering with nature” (strongly disagree = 2.1%, somewhat disagree = 3.8%, neither agree nor disagree = 10.2%. somewhat agree = 38.9%, strongly agree = 38.9%) and “the occurrence of wildlife disease has been made worse by humans and their activities” (strongly disagree = 1.7%, somewhat disagree = 4.2%, neither agree nor disagree = 19.8%, somewhat agree = 38.9%, strongly agree = 35.4%). Most respondents were very or extremely concerned about pathogen transmission from captive amphibians and reptiles to other captive animals, native wildlife, pets, livestock, and humans (median = very concerned, n = 2,007; S9 Table). These items used to measure respondents’ ‘sensitivity to herpetological trade risks’ represented a single scale (Cronbach’s alpha≥0.893; RMSEA≤0.043, CFI≥0.971). All indicators of sensitivity to herpetological trade risk were significant at the p<0.001 level (standardized coefficients≥0.60; S10 Table).

Respondents had limited prior knowledge of pathogen transmission through the herpetological trade. Respondents thought that approximately half of captive amphibians and reptiles (mean = 49.4%, median = 49%) in the live animal trade are healthy animals. Only 215 respondents (10.7%) had read or seen any news on the pathogen transmission risk of the live amphibian and reptile trade in the past year (no: 78.3%; I’m not sure: 11.0%). Most respondents had not heard of chytridiomycosis (no: 92.3%, yes: 5.0%, I’m not sure: 2.7%) or ranavirosis (no: 86.2%, yes: 8.9%, I’m not sure: 5.0%) before, but they had heard of salmonellosis (no: 3.9%, yes: 95.6%, I’m not sure: 0.6%). In total, 1,012 respondents (50.4%) stated that they knew that amphibians and reptiles could transmit *Salmonella* to humans. Most respondents thought that the risks that *Bd*, *Bsal*, ranaviruses and *Salmonella* would be transmitted from captive herpetofauna to other organisms (captive herpetofauna, native herpetofauna, native fish, pets, livestock, and/or humans) were high or very high (median = high, n = 2,007; S11 Table). Cronbach’s alpha (≥0.925) and CFA (RMSEA≤0.05) confirmed that these items used to measure respondents’ ‘perceived susceptibility to herpetological pathogen transmission’ represented a single scale (standardized coefficients≥0.61, p<0.05; S12 Table).

Most respondents who were presented with the ecological impacts of pathogen transmission were very or extremely concerned about the transmission of *Bd*, *Bsal* and ranaviruses to other herpetofauna and native fish and the loss of biodiversity from the disease-related deaths of native amphibians and reptiles (median = very concerned, n = 995; S13 Table). The items used to measure respondents’ ‘sensitivity to the ecological risks’ associated with pathogen transmission through the herpetological trade represented a single scale (Cronbach’s alpha>0.95, RMSEA<0.04, CFI>0.98, standardized coefficients≥0.79, p<0.001; S14 Table). Most respondents thought that there was a high or very high risk that diseases could result in a loss of biodiversity (none = 1.4%, low = 5.6%, moderate = 22.6%, high = 37.5%, very high = 32.9%; n = 995).

On average, respondents who were presented with the economic impacts of pathogen transmission were very concerned about negative economic impacts to agriculture and aquaculture (median = very concerned) and moderately concerned about negative economic impacts to the pet trade and frog leg market (median = moderately concerned, n = 995; S15 Table). These items used to measure respondents’ ‘sensitivity to the economic risks’ associated with pathogen transmission through the herpetological trade represented a single scale (Cronbach’s alpha≥0.838; RMSEA≤0.03; CFI>0.99; standardized coefficients≥0.55, p<0.001; S16 Table). Most respondents considered the risk that diseases could result in negative economic impacts to agriculture, aquaculture, the pet trade and the frog leg market to be high or very high (n = 995; S17 Table). These items used to measure respondents’ ‘perceived susceptibility to the economic risks’ associated with pathogen transmission through the herpetological trade represented a single scale (Cronbach’s alpha>0.86; RMSEA≤0.05; CFI>0.98; standardized factor loadings≥0.56, p<0.001; S18 Table).

On average, respondents who were presented with the human health and wellbeing impacts of pathogen transmission were very concerned about the transmission of *Salmonella* from captive herpetofauna to other amphibians and reptiles in the live animal trade, native herpetfauna, pets, livestock, and humans (median = very concerned, n = 993; S19 Table). Most respondents were very or extremely concerned about an increase in insect pests (not at all = 2.2%, slightly = 7.4%, moderately = 22.2%, very = 38.5%, extremely = 29.8%) or insect-borne diseases (not at all = 2.0%, slightly = 6.4%, moderately = 19.5%, very = 38.4%, extremely = 33.6%) from disease-related deaths of native amphibians and reptiles. The items used to measure respondents’ ‘sensitivity to human health and wellbeing risks’ represented a single scale (Cronbach’s alpha>0.9; RMSEA≤0.05; CFI>0.95; standardized coefficients≥0.55, p<0.001; S20 Table). Most respondents thought the risks that diseases would result in an increase in insect pests (none = 1.0%, low = 6.0%, moderate = 22.2%, high = 39.7%, very high = 31.1%) or insect-borne diseases (none = 1.3%, low = 5.5%, moderate = 21.5%, high = 39.2%, very high = 32.5%) were high or very high.

### Social trust

On average, respondents neither agreed nor disagreed with the statements used to measure social trust (median = ‘neither agree nor disagree’, n = 2,007; S21 Table). Cronbach’s alpha (>0.8) and CFA (CFI>0.95) confirmed that the items used to measure ‘social trust’ represented a single scale. All indicators of social trust had substantial standardized loadings (≥0.55) that were significant at the p<0.001 level across the different survey versions (S22 Table).

### Respondents’ values

Based on the E-PVQ, respondents most strongly endorsed the statements “It is important to respect nature” (biospheric value) and “It is important that every person is treated justly” (altruistic value; median response of ‘very much like me’; S23 Table). The items used to measure ‘biospheric values’ generated a single scale (Cronbach’s alpha>0.85, CFI>0.95), each with statistically significant (p<0.001) standardized loadings (≥0.71; S24 Table). Similarly, the items used to measure ‘altruistic values’ (Cronbach’s alpha>0.78; RMSEA<0.05; CFI>0.99; standardized coefficients≥0.52, p<0.001; S25 Table) and ‘hedonic values’ were unidimensional (Cronbach’s alpha>0.78; standardized coefficients≥0.67, p<0.001; S26 Table). Cronbach’s alpha for the items that were used to measure ‘egoistic values’ was adequate (≥0.72). After accounting for error correlation between items, we determined that these items generated a single scale (RMSEA<0.05; CFI>0.99; all standardized loadings≥0.5, p<0.001; S27 Table). De-identified survey data are presented in S2 Appendix.

### Structural equation models

**Fig 2:**
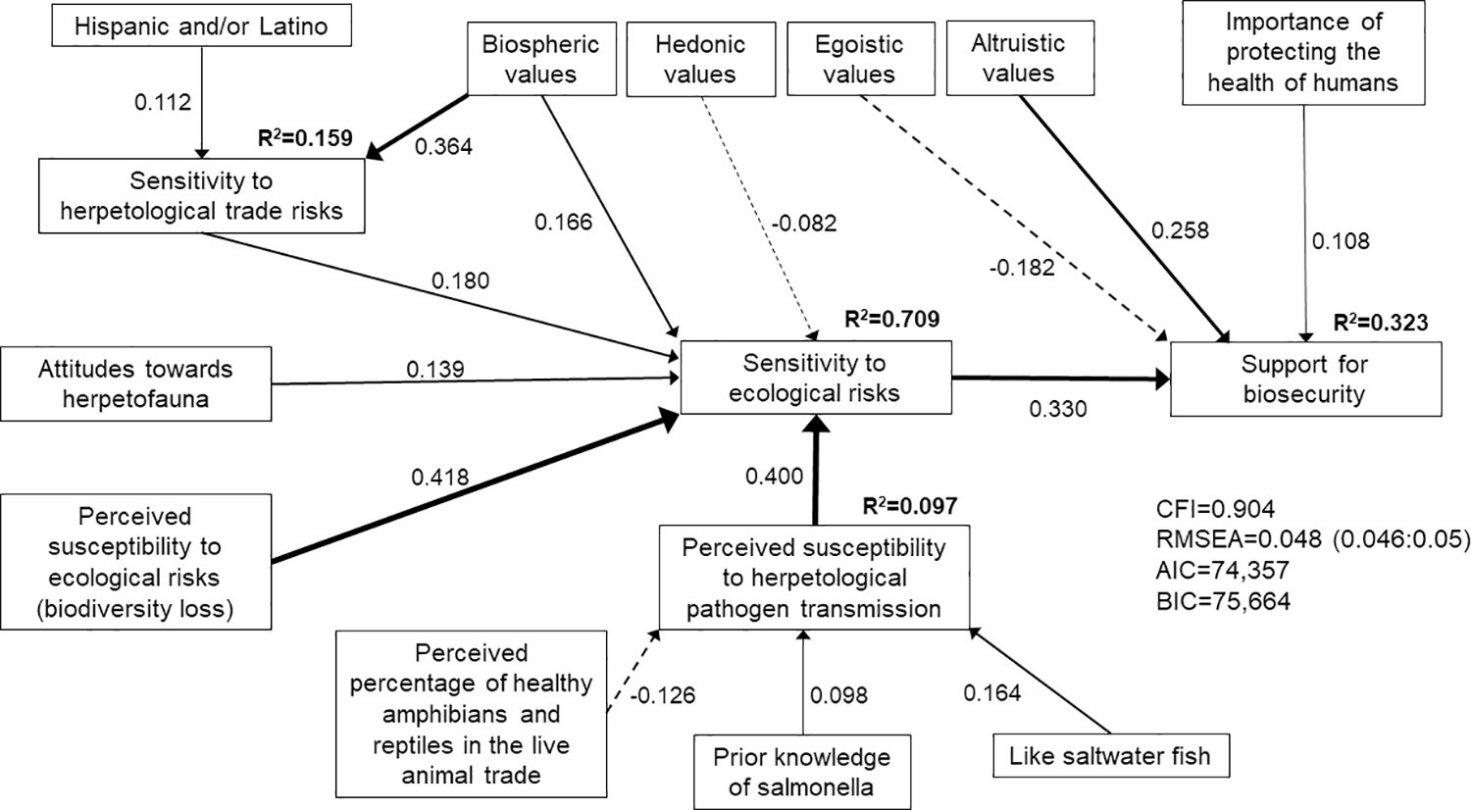
Structural equation model of direct and indirect determinants of respondents’ support for improved biosecurity measures when presented with the ecological risks associated with pathogen transmission through the live herpetological trade (model 1; n = 507). Solid lines indicate positive correlations. Dashed lines indicate negative correlations. Line weight indicates strength of correlation.

**Fig 3:**
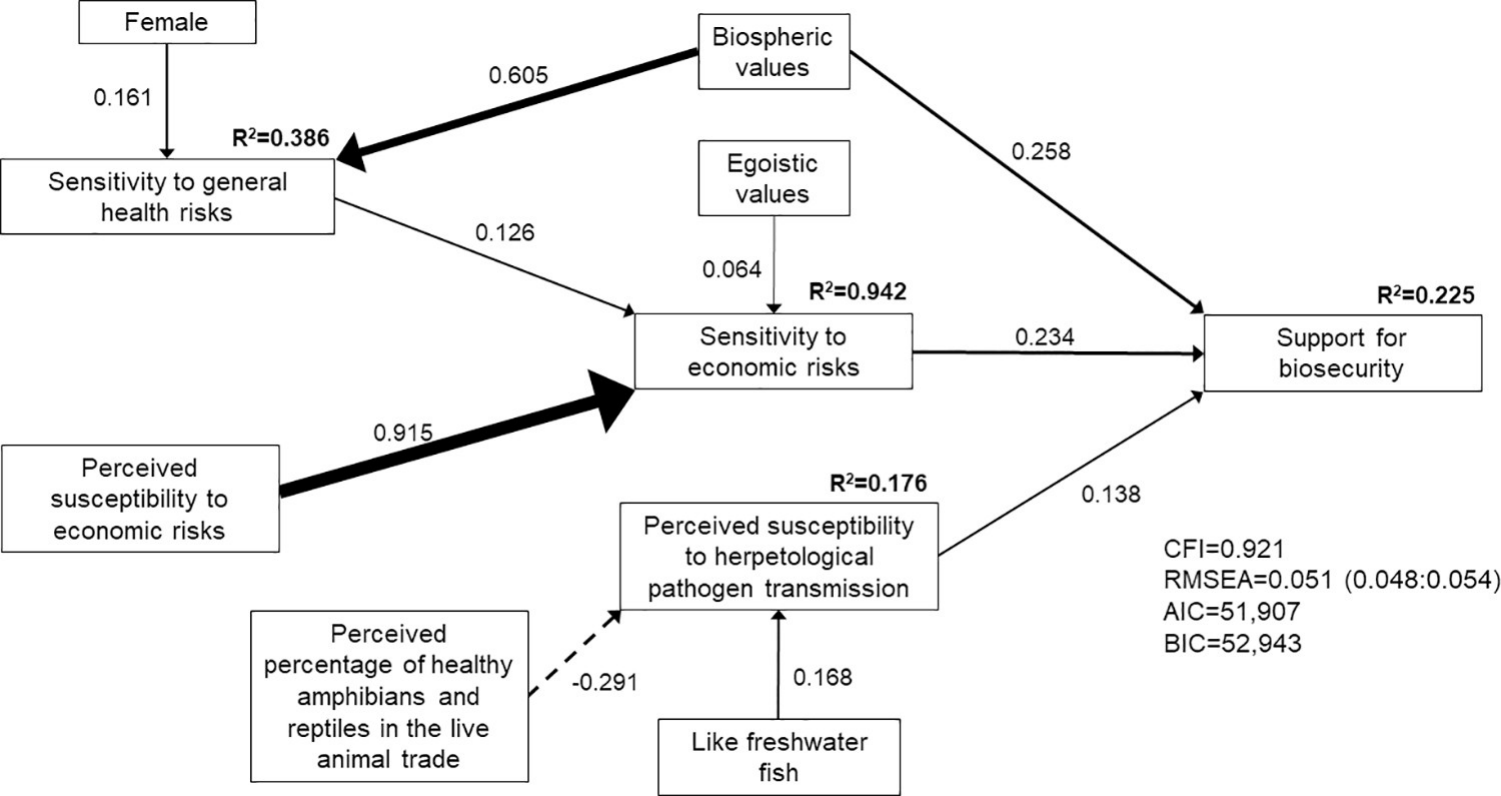
Structural equation model of direct and indirect determinants of respondents’ support for improved biosecurity measures when presented with the economic risks associated with pathogen transmission through the live herpetological trade (model 2; n = 507). Solid lines indicate positive correlations. Dashed lines indicate negative correlations. Line weight indicates strength of correlation.

**Fig 4:**
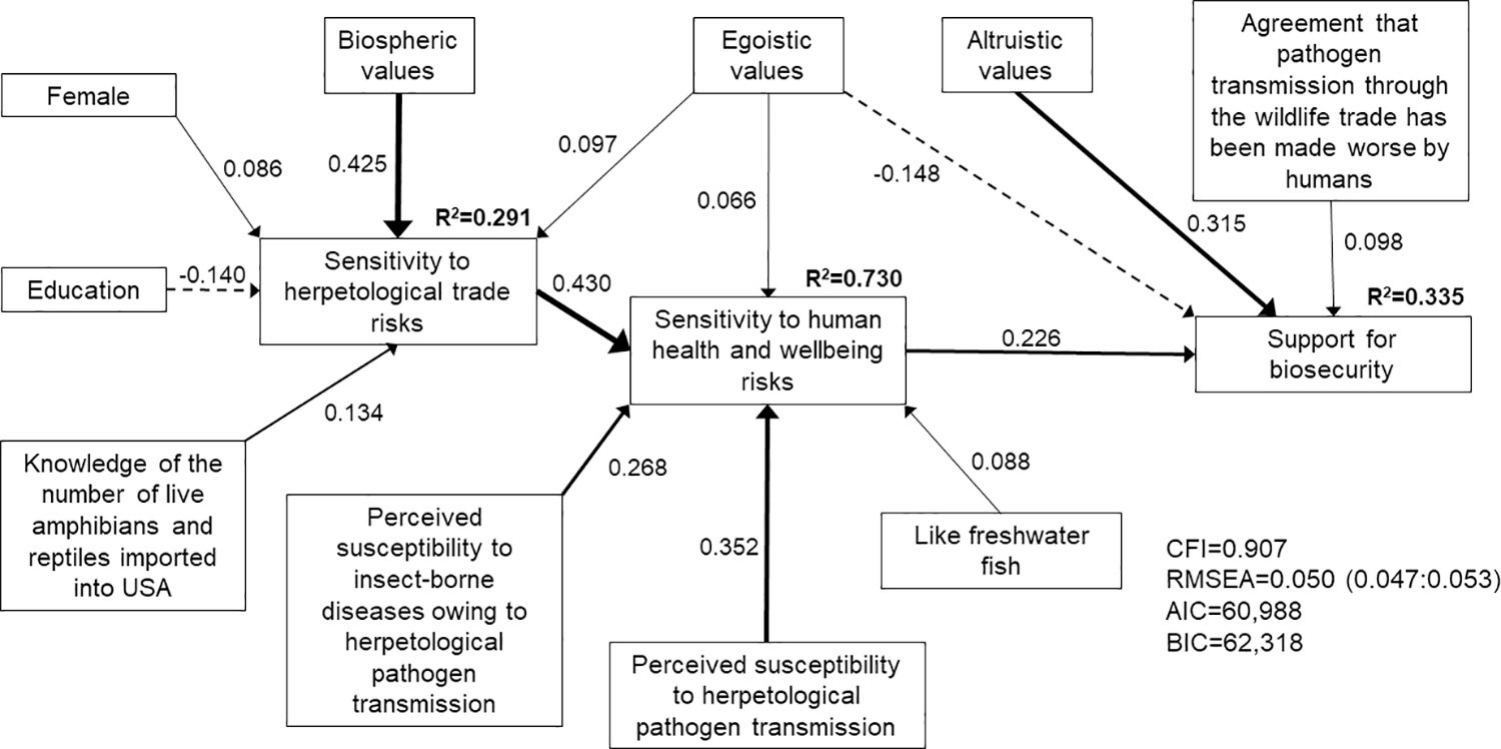
Structural equation model of direct and indirect determinants of respondents’ support for improved biosecurity measures when presented with the human health and wellbeing risks associated with pathogen transmission through the live herpetological trade (model 3; n = 502). Solid lines indicate positive correlations. Dashed lines indicate negative correlations. Line weight indicates strength of correlation.

**Fig 5:**
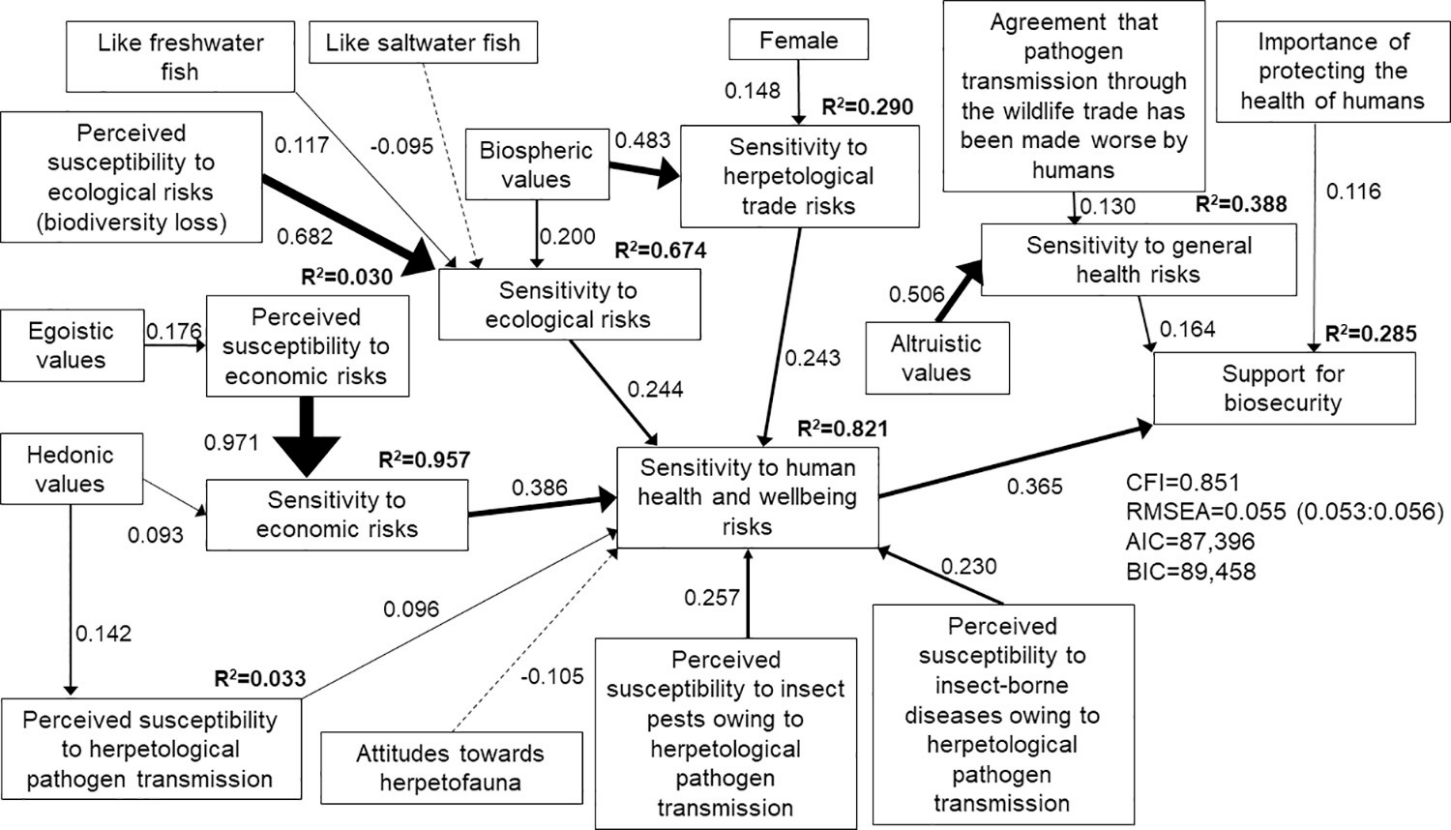
Structural equation model of direct and indirect determinants of respondents’ support for improved biosecurity measures when presented with the ecological, economic, and human health and wellbeing risks associated with pathogen transmission through the live herpetological trade (model 4; n = 498). Solid lines indicate positive correlations. Dashed lines indicate negative correlations. Line weight indicates strength of correlation.

The ecological (model 1; Fig 2), economic (model 2; Fig 3), and human health and wellbeing (model 3; Fig 4) SEMs all met the criteria for good model fit. However, the best fit model that included all hazards associated with pathogen transmission (model 4; Fig 5) did not meet the criteria of CFI≥0.90. Nonetheless, we present insights from model 4 because RMSEA = 0.055 (0.053:0.056) and the estimated coefficients made sense, i.e., two of the three model fit criteria were met. We focus on the structural regression component of the SEMs below, although the complete model specifications that include the measurement models are provided in the supporting information (S28–S31 Tables).

Consistent with our prior predictions, respondents’ support for biosecurity measures was positively correlated with their risk sensitivity. Respondents with higher sensitivity to ecological risks (β = 0.330, p<0.001, model 1; Fig 2), economic risks (β = 0.234, p<0.001, model 2; Fig 3) and human health and wellbeing risks (β = 0.226, p<0.001, model 3; Fig 4) associated with pathogen transmission through the herpetological trade were more likely to support biosecurity measures. When all three types of risks were presented to respondents, their support for biosecurity measures was directly positively correlated with their sensitivity to human health and wellbeing risks (β = 0.366, p<0.001, model 4; Fig 5), and indirectly positively correlated with their sensitivity to ecological and economic risks. Model 4 suggested that respondents with higher sensitivity to ecological (β = 0.243, p<0.001) or economic risks (β = 0.387, p<0.001) also had higher sensitivity to human health and wellbeing risks associated with pathogen transmission.

In turn, respondents’ sensitivity to risks was positively correlated with their perceived susceptibility to risks. Respondents who were more concerned about the loss of biodiversity owing to herpetological pathogen transmission had higher sensitivity to ecological risks (β = 0.418, p<0.001, model 1; β = 0.682, p<0.001, model 4). Respondents with higher perceived susceptibility to economic risks had higher sensitivity to economic risks (β = 0.915, p<0.001, model 2; β = 0.970, p<0.001, model 4). Similarly, respondents with higher perceived susceptibility to human health and wellbeing risks had higher sensitivity to these risks. Respondents who expressed greater concern about an increase in insect pests (β = 0.257, p<0.001, model 4) and insect-borne diseases owing to herpetological pathogen transmission were more likely to be sensitive to human health and wellbeing risks (β = 0.268, p<0.001, model 3; β = 0.230, p<0.001, model 4).

Respondents with higher perceived susceptibility to herpetological pathogen transmission were more sensitive to ecological risks (β = 0.400, p<0.001, model 1) and human health and wellbeing risks (β = 0.352, p<0.001, model 3; β = 0.096, p = 0.024, model 4). We also found a direct positive correlation between respondents’ perceived susceptibility to herpetological pathogen transmission and their support for biosecurity for the SEM model that focused on economic risks (β = 0.138, p = 0.026, model 2). However, respondents who believed that captive herpetofauna in the live wildlife trade are healthy were less likely to perceive susceptibility to herpetological pathogen transmission (β = -0.126, p<0.001, model 1; β = -0.291, p<0.001, model 2). We found some evidence that respondents with prior knowledge of salmonellosis were more likely to perceive susceptibility to herpetological pathogen transmission (β = 0.098, p = 0.016, model 1).

Respondents with prior knowledge of the number of live herpetofauna imported into the United States (β = 0.134, p = 0.002, model 3) and prior knowledge of herpetological imports (β = 0.107, p = 0.036, model 4) were more sensitive to herpetological trade risks, which in turn increased their sensitivity to human health and wellbeing risks (β = 0.430, p<0.001, model 3; β = 0.243, p<0.001, model 4). Our results further suggested that respondents with greater sensitivity to herpetological trade risks had greater sensitivity to the ecological risks associated with herpetological pathogen transmission (β = 0.180, p<0.001, model 1). Women (β = 0.086, p = 0.042, model 3; β = 0.151, p = 0.001, model 4) and Hispanic and/or Latino respondents (β = 0.112, p = 0.005, model 1) demonstrated greater sensitivity to herpetological trade risks, whereas respondents with more years of education demonstrated lower sensitivity (β = -0.140, p = 0.001, model 3).

Women also demonstrated greater sensitivity to general health risks (β = 0.161, p<0.001, model 2). Respondents’ sensitivity to general health risks was positively correlated with their support for biosecurity measures (positive correlation with economic risks for model 2: β = 0.126, p<0.001; direct positive correlation with support for biosecurity for model 4: β = 0.164, p = 0.002). Similarly, respondents who placed importance on protecting the health of humans demonstrated greater support for biosecurity (β = 0.108, p = 0.020, model 1; β = 0.116, p = 0.013, model 4). Respondents who agreed that disease prevalence has been made worse by humans and their activities (moral concerns) were more likely to support biosecurity measures (direct positive correlation with support for model 3: β = 0.098, p = 0.025; positive correlation with sensitivity to general health risks for model 4: β = 0.130, p = 0.012).

Respondents who liked herpetofauna were more sensitive to ecological risks associated with herpetological pathogen transmission (β = 0.139, p<0.001, model 1). By contrast, these individuals were less sensitive to human health and wellbeing risks associated with herpetological pathogen transmission when taking all possible risks associated with pathogen transmission into account (β = -0.104, p = 0.001, model 4). We found a positive correlation between whether respondents liked fish and their perceptions of susceptibility to herpetological pathogen transmission (β = 0.164, p = 0.008 for saltwater fish in model 1; β = 0.168, p = 0.046 for freshwater fish in model 2), their sensitivity to ecological risks (β = 0.117, p = 0.013 for freshwater fish in model 4), and their sensitivity to human health and wellbeing risks (β = 0.088, p = 0.032 for freshwater fish in model 3).

We found no evidence that social trust influenced support for biosecurity (p>0.05 across models). However, respondents’ values were important determinants of their support for biosecurity measures. Respondents with biospheric values demonstrated greater sensitivity to ecological risks (β = 0.166, p<0.001, model 1; β = 0.201, p<0.001, model 4), herpetological trade risks (β = 0.364, p<0.001, model 1; β = 0.425, p<0.001, model 3; β = 0.481, p<0.001, model 4) and general health risks (β = 0.605, p<0.001, model 2). We also found a direct positive correlation between biospheric values and respondents’ support for biosecurity measures for the economic risks SEM (β = 0.258, p<0.001, model 2). Similarly, we found a direct positive correlation between altruistic values and support for biosecurity measures for two of the models (β = 0.258, p<0.001, model 1; β = 0.315, p<0.001, model 3). Respondents with strong altruistic values were also more sensitive to general health risks (β = 0.505, p<0.001, model 4). We found a direct negative correlation between egoistic values and support for biosecurity measures for the SEM models that stressed the ecological and human health and wellbeing hazards associated with pathogen transmission (β = -0.182, p = 0.007, model 1; β = -0.148, p = 0.001, model 3). However, respondents with egoistic values were more likely to be sensitive to economic risks (β = 0.064, p = 0.014, model 2) and to perceive susceptibility to economic risks associated with herpetological pathogen transmission (β = 0.174, p<0.001, model 4). Respondents with egoistic values also demonstrated greater sensitivity to human health and wellbeing risks (β = 0.066, p = 0.038, model 3) and herpetological trade risks (β = 0.097, p = 0.037, model 3), thereby indirectly increasing their support for biosecurity. Finally, we found some evidence that respondents with strong hedonic values were less likely to be sensitive to ecological risks (β = -0.082, p = 0.022, model 1) but more likely to be sensitive to economic risks associated with herpetological pathogen transmission (β = 0.093, p = 0.001, model 4) and to perceive susceptibility to this pathogen transmission (β = 0.141, p = 0.001, model 4).

## Discussion

Securing public support for biosecurity measures depends on a clear understanding of the public’s opinions, values, and goals, in order to increase public trust in these measures and the need for their implementation [35]. We found strong support for increased quarantine and veterinary observation, mandatory tests for diseases of concern, and best practices to reduce stress and improve the care of live herpetofauna, in order to reduce pathogen transmission through the herpetological trade. In contrast to previous studies [6,27,29,30,32,34], we found no evidence that respondents’ support for biosecurity depended on their trust in government, or their age, children under the age of 18, ownership of pets or domestic animals, engagement in recreational activities (e.g., fishing), and exposure to the live herpetological trade (consumption of frog legs, use of salamanders as fishing bait, or acquaintance with an individual who owns herpetofauna). We found some, albeit limited, evidence that women and Hispanic or Latino members of the public were more sensitive to risks associated with the live herpetological trade, whereas more educated individuals were less sensitive to these risks [24]. Rather, respondents’ risk perceptions and values played a key role in determining their support for biosecurity, which is consistent with previous findings that the public’s attitudes, beliefs, and risk perceptions are stronger determinants of their support for managing invasion risks associated with the live animal trade than their demographic characteristics [53,54]. Our finding that respondents’ socio-psychological characteristics were key determinants of their support for biosecurity has important implications for communications and messaging about biosecurity.

Respondents’ support for biosecurity strongly depended on their sensitivity to risk, which was determined by both the hazards being evaluated and their perceived susceptibility to risk [24,32]. As we predicted, individuals with strong biospheric values demonstrated greater sensitivity to ecological risks associated with pathogen transmission. These individuals also demonstrated greater sensitivity to herpetological trade risks, expressing concern about pathogen transmission from captive herpetofauna to other captive animals, native wildlife, pets, livestock, and humans. Although we did not find a direct link between respondents’ altruistic values and their sensitivity to human health and wellbeing risks associated with pathogen transmission, respondents with strong altruistic values placed importance on protecting the health of captive animals, native wildlife, the natural environment, pets, livestock, and humans. Altruistic individuals were also supportive of biosecurity when the ecological and human health and wellbeing hazards associated with the herpetological trade were highlighted. This suggests that individuals with strong self-transcendence (biospheric, altruistic) values will be receptive to One Health justifications for improved biosecurity related to the live herpetological trade, which is consistent with previous findings that these individuals tend to support interventions that improve environmental quality and secure the health and wellbeing of current and future generations [31,39,40].

Although prior studies have found weak and inconsistent relationships between self-enhancing (egoistic, hedonic) values and support for conservation measures [38,39], our findings suggest that individuals with strong egoistic values may be persuaded to support biosecurity measures for the live herpetological trade if the economic and public health risks associated with pathogen transmission are communicated. Highlighting adverse economic impacts to industries, and associated losses of revenues and employment, may resonate with egoistic individuals. We also found some evidence that egoistic individuals recognized the important role that the herpetological trade may play in transmitting pathogens and the associated negative impacts to human health and wellbeing. Egoistic individuals may be sensitive to these risks because they recognize the costs of healthcare and value their health. Although only *Salmonella* could be transmitted to humans, we highlighted that declines in herpetofauna populations driven by *Bd*, *Bsal* and ranaviruses could increase pest populations and transmission of insect-borne diseases. Emphasizing public health risks [55] and resilience (i.e., measures to secure communities’ economic welfare and way of life [56]) has been shown to be an effective messaging strategy to gain support for climate change interventions amongst politically conservative individuals. A similar approach may be used to tailor One Health justifications for mitigating herpetological pathogen transmission to individuals with strong self-enhancing values and those who disagree that environmental problems and pathogen transmission are exacerbated by humans (i.e., individuals who do not have moral concerns about human interference with nature and/or consider disease to be natural). However, we note other researchers’ [24,30] caution that messaging strategies pertaining to pathogen transmission by wildlife that emphasize protecting human health and economic interests may have unintended negative consequences.

Emphasizing health effects on valued wildlife species may resonate with individuals with strong self-enhancing values, as well as individuals with strong self-transcendence values, when these pathogens are not zoonotic [30]. Unfortunately, herpetofauna are typically not considered charismatic species, and members of the general public often hold negative attitudes and emotions towards these species [57,58]. It is therefore unsurprising that attitudes towards herpetofauna only appeared as a positive, indirect determinant of support for biosecurity in the model that emphasized the ecological risks associated with pathogen transmission by the herpetological trade. Respondents who liked herpetofauna (including snakes, which are often feared [57,58]) demonstrated greater sensitivity to the ecological risks associated with pathogen transmission–which is consistent with the finding that biophilia results in increased emotional connection to nature [58]. By contrast, liking for fish indirectly increased support for biosecurity across models, suggesting that people’s support for biosecurity could be attained by increasing their awareness of how pathogen transmission may adversely impact fish biodiversity [59] and economically and culturally important fisheries [60]. However, more research is needed to ascertain how attitudes towards herpetofauna and fish might influence support for biosecurity, since our measure of attitudes was restricted to asking whether respondents liked these taxa.

We found little evidence that respondents’ risk perceptions or support for biosecurity depended on their knowledge of the live herpetological trade or pathogen transmission risks associated with this trade. Nonetheless, respondents’ general lack of knowledge of the scale of the live herpetological trade, the number of industries that may contribute to the spread of pathogens, the prevalence of pathogens in the herpetological trade, the existence of *Bd*, *Bsal* and ranaviruses, and that *Salmonella* may be transmitted to humans by herpetofauna demonstrates that improved education and outreach on these topics is required. Imparting this information to the public may generate or reinforce intentions to support biosecurity measures. Given well-documented gaps between knowledge and behavior [61,62], we stress that information should be presented using different message framings that will resonate with members of the public who hold different values and beliefs [54,63]. Equally importantly, education and messaging should provide the public with suggested actions they can take to mitigate pathogen transmission risks. Our study focused on biosecurity actions that were unlikely to directly impact respondents in terms of cost or inconvenience, but our findings may also be used to consider how improved biosecurity behaviors by individuals may be leveraged by combining appropriate messaging with actions to make these behaviors easier to adopt (e.g., providing bait disposal containers at fishing sites [62]).

## Conclusions

Trade in live herpetofauna poses serious ecological, economic, and public safety risks through the transmission of pathogens to native wildlife, domestic animals, and humans. Although improved biosecurity is required in the United States to mitigate pathogen transmission, decision-makers may be loath to implement effective biosecurity measures if they are concerned about public opposition to these measures. We found strong support across members of the public for biosecurity measures to mitigate pathogen transmission through the herpetological trade, namely increased quarantine and veterinary observation of herpetofauna imports, mandatory tests for diseases of concern for imported herpetofauna, and best practices to reduce stress and improve the care of live herpetofauna during transport. Respondents’ values and their perceived susceptibility and sensitivity to different hazards associated with pathogen transmission by captive herpetofauna were key determinants of their support for biosecurity. Different messages should be tailored to members of the public with different values to elicit their support for biosecurity. Our results suggest that individuals with strong biospheric or altruistic values are likely to respond to messaging about the transmission of pathogens from captive herpetofauna to native animals, domestic animals, and humans as well as the ecological impacts associated with pathogen transmission (such as loss of biodiversity). Individuals with strong egoistic values are likely to respond to messaging about the economic and public health risks associated with pathogen transmission. When possible, communication about pathogen transmission should also provide suggested actions that individuals can take to enhance the effectiveness of government-implemented biosecurity.

## Supporting information

S1 Appendix Survey questionnaire. Multiple images used in the survey have been omitted because not all images are part of the creative commons.
(PDF)

S2 Appendix De-identified data set. (XLSX)

S1 Table Survey respondents’ demographic characteristics and ownership of domestic animals. (PDF)

S2 Table Confirmatory factor analysis for respondents’ support for biosecurity for different survey versions that presented the ecological impacts, economic impacts, human health and wellbeing impacts, or all impacts of pathogen transmission. (PDF)

S3 Table Distribution of respondents’ prior knowledge of reasons for herpetological imports (n = 2,007). (PDF)

S4 Table Confirmatory factor analysis for respondents’ prior knowledge of reasons for herpetological imports (‘knowledge of herpetological imports’) for different survey versions that presented the ecological impacts, economic impacts, human health and wellbeing impacts, or all impacts of pathogen transmission. (PDF)

S5 Table Distribution of responses to the question “How much do you like or dislike the following animals?” (n = 2,007). (PDF)

S6 Table Confirmatory factor analysis for respondents’ ‘attitudes towards herpetofauna’ for different survey versions that presented the ecological impacts, economic impacts, human health and wellbeing impacts, or all impacts of pathogen transmission. (PDF)

S7 Table Distribution of responses to the questions used to measure respondents’ sensitivity to general health risks (n = 2,007). (PDF)

S8 Table Confirmatory factor analysis for the importance that respondents placed on protecting the health of animals in the live animal trade, native wildlife, the natural environment, pets, and livestock (‘sensitivity to general health risks’) for different survey versions that presented the ecological impacts, economic impacts, human health and wellbeing impacts, or all impacts of pathogen transmission. (PDF)

S9 Table Distribution of responses to the questions used to measure respondents’ sensitivity to herpetological trade risks (n = 2,007). (PDF)

S10 Table Confirmatory factor analysis for respondents’ level of concern about pathogen transmission from captive herpetofauna to other captive animals, native wildlife, pets, livestock, and humans (‘sensitivity to herpetological trade risk’) for different survey versions that presented the ecological impacts, economic impacts, human health and wellbeing impacts, or all impacts of pathogen transmission. (PDF)

S11 Table Distribution of respondents’ risk perceptions pertaining to transmission of Bd, Bsal, ranaviruses and Salmonella by captive herpetofauna (n = 2,007). (PDF)

S12 Table Confirmatory factor analysis for respondents’ ‘susceptibility to herpetological pathogen transmission’ for different survey versions that presented the ecological impacts, economic impacts, human health and wellbeing impacts, or all impacts of pathogen transmission. (PDF)

S13 Table Distribution of respondents’ risk concerns about the ecological impacts of pathogen transmission through the herpetological trade (n = 995). (PDF)

S14 Table Confirmatory factor analysis for respondents’ concern pertaining to ecological impacts of pathogen transmission through the herpetological trade (‘sensitivity to ecological risks’). (PDF)

S15 Table Distribution of respondents’ risk concerns about the economic impacts of pathogen transmission through the herpetological trade (n = 995). (PDF)

S16 Table Confirmatory factor analysis for respondents’ concern pertaining to economic impacts of pathogen transmission through the herpetological trade (‘sensitivity to economic risks’). (PDF)

S17 Table Distribution of respondents’ risk perceptions related to the economic impacts of pathogen transmission through the live herpetological trade (n = 995). (PDF)

S18 Table Confirmatory factor analysis for respondents’ perceived ‘susceptibility to economic risks’ associated with pathogen transmission through the live herpetological trade. (PDF)

S19 Table Distribution of respondents’ concerns about the human health and wellbeing impacts of pathogen transmission through the live herpetological trade (n = 993). (PDF)

S20 Table Confirmatory factor analysis for respondents’ concern pertaining to human health and wellbeing impacts of pathogen transmission through the live herpetological trade (‘sensitivity to human health and wellbeing risks’). (PDF)

S21 Table Respondents’ trust in the government to mitigate pathogen transmission risks associated with the live herpetological trade (n = 2,007). (PDF)

S22 Table Confirmatory factor analysis for respondents’ ‘social trust’ for different survey versions that presented the ecological impacts, economic impacts, human health and wellbeing impacts, or all impacts of pathogen transmission. (PDF)

S23 Table Distribution of responses to the Environmental Portrait Value Questionnaire: “Below are some statements about a random man/woman/person. How similar is this person to you?” (n = 2,007).
(PDF)

S24 Table Confirmatory factor analysis for respondents’ ‘biospheric values’. (PDF)

S25 Table Confirmatory factor analysis for respondents’ ‘altruistic values’. (PDF)

S26 Table Confirmatory factor analysis for respondents’ ‘hedonic values’. (PDF)

S27 Table Confirmatory factor analysis for respondents’ ‘egoistic values’. (PDF)

S28 Table Structural equation model of respondents’ support for improved biosecurity measures when presented with the ecological risks associated with pathogen transmission through the live herpetological trade (model 1, n = 507). (PDF)

S29 Table Structural equation model of respondents’ support for improved biosecurity measures when presented with the economic risks associated with pathogen transmission through the live herpetological trade (model 2, n = 507). (PDF)

S30 Table Structural equation model of respondents’ support for improved biosecurity measures when presented with the human health and wellbeing risks associated with pathogen transmission through the live herpetological trade (model 3, n = 505). (PDF)

S31 Table Structural equation model of respondents’ support for improved biosecurity measures when presented with the ecological, economic, and human health and wellbeing risks associated with pathogen transmission through the live herpetological trade (model 4, n = 488). (PDF)
