# Supplementary material for: Investigating public support for biosecurity measures to mitigate pathogen transmission through the herpetological trade
Source: PLoS One. 2022 Jan 21;17(1):e0262719. doi: 10.1371/journal.pone.0262719 (PMC8782347; doi:10.1371/journal.pone.0262719)
Supplement: S1 Appendix — Multiple images used in the survey have been omitted because not all images are part of the creative commons. (PDF) [file pone.0262719.s001.pdf]

Opinions on the Legal Trade in Live animals

This questionnaire will provide you with information about the legal trade in live animals, and ask you about the legal trade in live animals.

Demographics

First, we will ask you a few background questions to ensure we've heard from all the different types of people who live in the United States.

What is your sex?
- Male
- Female
- I prefer not to say

What is your age?
- Under 18 years
- 18 to 24 years
- 25 to 34 years
- 35 to 44 years
- 45 to 54 years
- 55 to 64 years
- 65 to 74 years
- 75 years or over

Are you Hispanic and/or Latino?
- Yes
- No
- I prefer not to say

Choose one or more races that you consider yourself to be:
- American Indian or Alaska Native
- Asian
- Black or African American
- Native Hawaiian or Pacific Islander
- White
- Other: ____________________
- I prefer not to say

What is the highest degree or level of school that you have completed?
- Less than 12th grade
- High school graduate or GED
- Some college / Associate or technical degree
- Bachelor's degree
- Graduate or professional degree

What state do you live in?

What is your zip code?

Are there any members in your household under the age of 18?

- Yes
- No

Do you currently have any pets?

- Yes
- No

What kind of pets do you own?

- Cat
- Dog
- Bird
- Fish
- Rodent or small mammal
- Reptile (such as a snake, lizard, turtle, or tortoise)
- Amphibian (such as a frog, toad, salamander, or newt)
- Insect / arachnid
- Other: _________________

Do you currently own any livestock or poultry?

- Yes, livestock
- Yes, poultry
- Yes, both
- No

Animals in the live animal trade are captured or bred so that they can be sold to different industries. These animals are alive when they are transported and sold.

Native wildlife are wild animals that live in an environment where they have been historically found.

Please rate how important it is to you to protect the **health** of:

	Not at all	Slightly	Moderately	Very	Extremely
Animals in the live animal trade	◻	◻	◻	◻	◻
Native wildlife	◻	◻	◻	◻	◻
The natural environment	◻	◻	◻	◻	◻
Pets	◻	◻	◻	◻	◻
Livestock, such as cows, sheep, and goats	◻	◻	◻	◻	◻
Humans	◻	◻	◻	◻	◻

Please indicate how much you agree with the following statements:

	Strongly disagree	Somewhat disagree	Neither agree nor disagree	Somewhat agree	Strongly agree
Most environmental problems are caused by humans interfering with nature	□	□	□	□	□
The occurrence of wildlife disease has been made worse by humans and their activities	□	□	□	□	□

This survey contains questions about amphibians, reptiles, and fish.

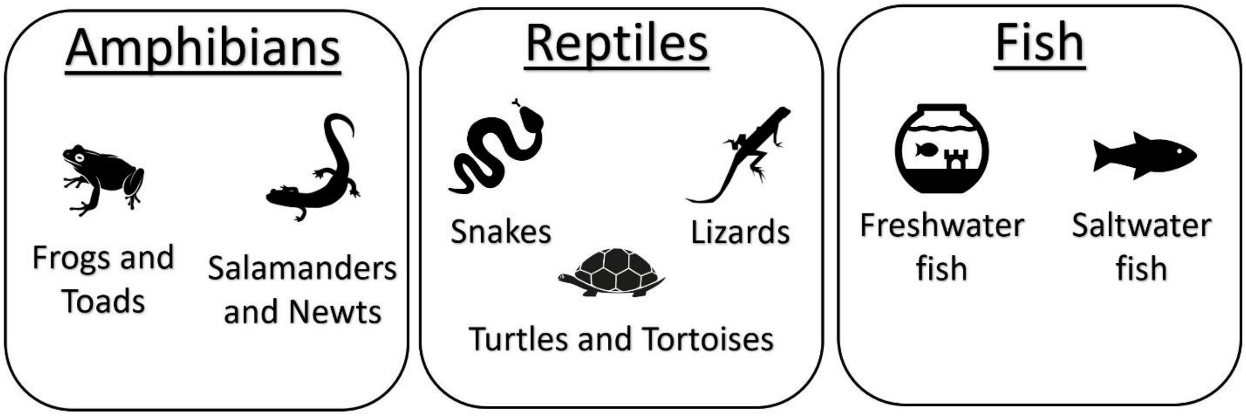

How much do you like or dislike the following animals?

	Strongly dislike	Dislike	Neutral	Like	Strongly like
Snakes	□	□	□	□	□
Lizards	□	□	□	□	□
Turtles/tortoises	□	□	□	□	□
Frogs	□	□	□	□	□
Toads	□	□	□	□	□
Salamanders/newts	□	□	□	□	□
Freshwater fish	□	□	□	□	□
Saltwater fish	□	□	□	□	□

The International Animal Trade

Animals are sold for several uses:

- Food
- Aquaculture
- Bait
- Medicine

- Pets
- Sport hunting
- Skin and fur
- Research/education
- Zoos

[*Images used to illustrate this question are omitted from this document.*]

How knowledgeable are you about the animal trade?

Not at all										Extremely
0	1	2	3	4	5	6	7	8	9	10

<u>Amphibians and Reptiles</u>

The rest of this survey focuses on the trade in live **amphibians** and **reptiles**. The United States is one of the **biggest importers of amphibians and reptiles in the world**.

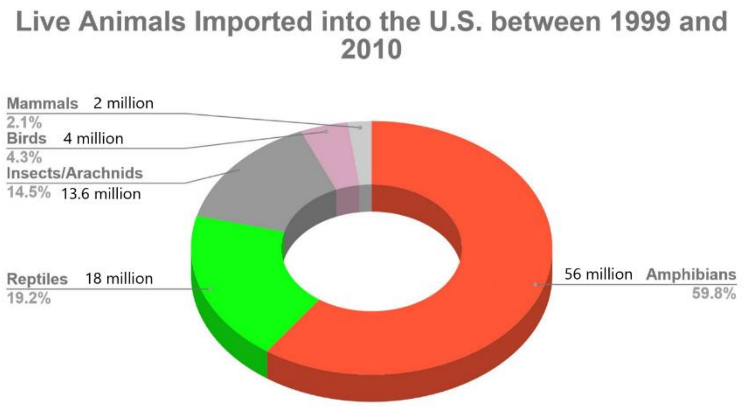

Is the number of live amphibians/reptiles imported into the United States lower or higher than you expected?

- Much lower than I expected
- Lower than I expected
- About what I expected
- Higher than I expected
- Much higher than I expected

This map gives you an idea of the geographic origins of amphibians and reptiles that are traded in the United States. It shows <u>imports</u> of **amphibians** and **reptiles** into the United States between 2009 and 2014.

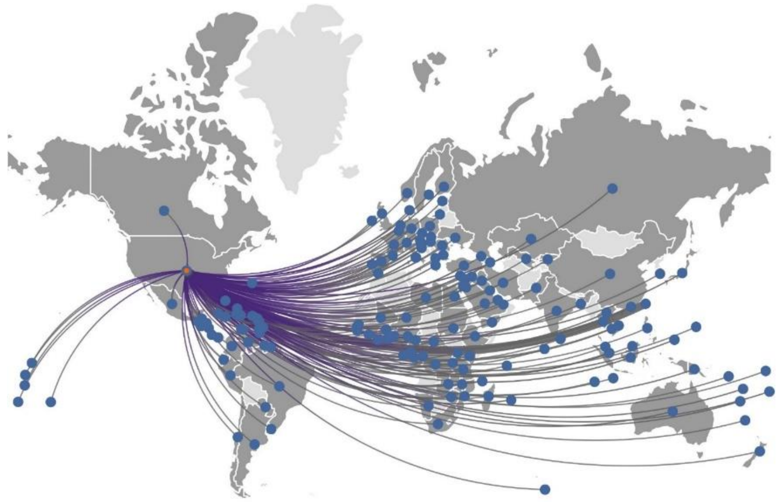

<u>The Live Amphibian and Reptile Trade</u>

The rest of the survey will focus on the trade in live frogs for human consumption; the use of amphibians as fishing bait; and the trade in amphibians and reptiles as pets.
[*Images used to illustrate this question are omitted from this document.*]

Were you aware that live animals are imported to the United States to supply these industries?

	Not at all aware	Slightly aware	Moderately aware	Highly aware
The trade in live frogs for human consumption	☐	☐	☐	☐
The use of amphibians as fishing bait	☐	☐	☐	☐
The amphibian and reptile pet industry	☐	☐	☐	☐

<u>Human Consumption</u>

People spend an estimated **<u>$45 million</u>** on frog legs globally each year.
The U.S. imports **<u>2,200 tons</u>** of live frogs each year for consumption.
[*Images used to illustrate this question are omitted from this document.*]

Have you eaten frog legs in the past year?

- Yes
- No
- I don't know

<u>Bait Trade</u>

Salamander larvae or 'water' dogs are a popular bait for fishermen across the United States.
In Arizona, up to **<u>75%</u>** of fishermen use salamanders as bait.

[*Images used to illustrate this question are omitted from this document.*]

Have you been fishing in the past year?

- Yes
- No

Do you use salamanders as fishing bait?

- Yes
- No
- I don't know

The Amphibian and Reptile Pet Trade

Many amphibians and **most reptiles** are imported into the U.S. for the pet trade.
4.7 million households in the U.S. have at least one pet reptile.
[*Images used to illustrate this question are omitted from this document.*]

Do you know anyone who owns a pet reptile or amphibian?

- Yes, a pet reptile
- Yes, a pet amphibian
- Yes, both
- No

A **captive** animal refers to a live animal that is being kept/transported and sold for the animal trade.

What **percentage** (out of 100%) of captive amphibians and reptiles in the live animal trade do you think are healthy animals? Click and drag the bar to where you feel is appropriate.

Healthy captive amphibians and reptiles	0	25	50	75	100

Disease Risks of the Live Amphibian and Reptile Trade

The trade in live amphibians and reptiles is economically important, but there are risks associated with the trade. One of those risks is the transmission of diseases.

The images below show how the live amphibian and reptile trade contributes to disease transmission.
[*Images used to illustrate this question are omitted from this document.*]

1. Stress from transport. Stress weakens an animal's immune system.
2. Inadequate care and nutrition. Stressed animals are more likely to be affected by diseases.
3. Housed in high densities. Other captive animals are exposed to the diseases.
4. Contact with humans. Animals can transmit diseases to humans.
5. Live animals or contaminated materials introduced in the wild. Diseases can be transmitted to wild animals by:
    a. People releasing pets and fish

b. People throwing away unused bait
c. People throwing out animal products or contaminated materials

Have you read anything or seen any news on the disease transmission risk of the live amphibian and reptile trade in the past year?

- Yes
- No
- I'm not sure

A **captive** animal refers to a live animal that is being kept/transported and sold for the animal trade.

How **<u>concerned</u>** are you about disease transmission from captive amphibians and reptiles to:

	Not at all	Slightly	Moderately	Very	Extremely
Other captive animals	□	□	□	□	□
Native wildlife	□	□	□	□	□
Pets	□	□	□	□	□
Livestock, such as cows, sheep, and goats	□	□	□	□	□
Humans	□	□	□	□	□

This survey will focus on 3 diseases that we will explain:

- Chytrid
- Ranavirus
- Salmonella

<u>Chytrid</u>: A disease that only affects **amphibians**.

- Amphibians absorb oxygen, water, and electrolytes through their skin.
- Chytrid thickens amphibians' skin. They die because they cannot breather or absorb water and electrolytes.

[*Images used to illustrate this question are omitted from this document.*]

Had you heard of chytrid prior to this survey?

- Yes
- No
- I'm not sure

<u>Chytrid</u>: How is it transmitted?

- Contact with an infected animal
- Contact with the chytrid fungus, which can survive up to **7 weeks in water or moist areas**

[*Images used to illustrate this question are omitted from this document.*]

What do you think the **risk** is that chytrid would be transmitted from captive amphibians to…

	None	Low	Moderate	High	Very high
Other captive amphibians	◻	◻	◻	◻	◻
Native amphibians	◻	◻	◻	◻	◻

Ranavirus: A virus that affects **amphibians**, **reptiles**, and **fish**
It causes:

- Fluid build-up under the skin
- Blood vessel damage
- Weakness and difficulty breathing

[*Images used to illustrate this question are omitted from this document.*]

Had you heard of ranavirus prior to this survey?

- Yes
- No
- I'm not sure

Ranavirus: How is it transmitted?

- Transmits across animals, for example from amphibians to fish, through:
  - Contact with the virus, an infected animal, or infected water
- The virus can **survive outside of a living host for more than 30 days**

[*Images used to illustrate this question are omitted from this document.*]

What do you think the **risk** is that ranavirus would be transmitted from captive amphibians and reptiles to…

	None	Low	Moderate	High	Very high
Other captive amphibians and reptiles	◻	◻	◻	◻	◻
Native amphibians and reptiles	◻	◻	◻	◻	◻
Native fish	◻	◻	◻	◻	◻

Salmonella: A bacterial infection that affects **animals** and **humans**
Humans experience

- Headaches
- Nausea and vomiting
- Fever and chills

[*Images used to illustrate this question are omitted from this document.*]

Had you heard of salmonella prior to this survey?

- Yes
- No
- I'm not sure

Salmonella: How is it transmitted?

- Reptiles and amphibians can carry the infection with no symptoms

- Humans catch it from contact with:
    - An infected animal, the animal's waste, or surfaces and infected animal has touched

[*Images used to illustrate this question are omitted from this document.*]

Prior to this survey, did you know that amphibians and reptiles could transmit salmonella to humans?

- Yes
- No

What do you think the **<u>risk</u>** is that salmonella would be transmitted from captive amphibians and reptiles to…

	None	Low	Moderate	High	Very high
Other captive amphibians and reptiles	◻	◻	◻	◻	◻
Native amphibians and reptiles	◻	◻	◻	◻	◻
Pets	◻	◻	◻	◻	◻
Livestock, such as cows, sheep, and goats	◻	◻	◻	◻	◻
Humans	◻	◻	◻	◻	◻

<u>Additional Impacts of Amphibian and Reptile Trade Diseases</u>

To recap, the different species affected by chytrid, ranavirus, and salmonella are:

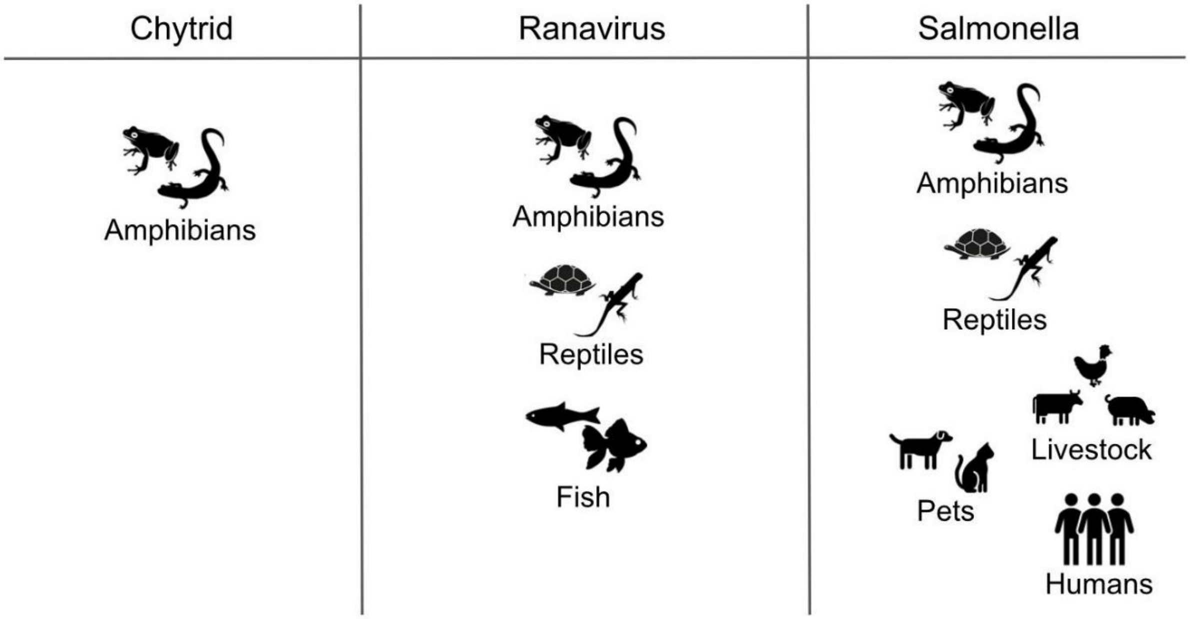

These diseases negatively affect the **<u>environment</u>**, **<u>economy</u>**, and **<u>human health</u>**.

Environmental Impacts

Chytrid: Currently infects 520+ species

- Has the potential to infect 6,000 species
- Linked to the **decline or extinction of at least 501** amphibian species

[*Images used to illustrate this question are omitted from this document.*]

How **concerned** are you about the spread of chytrid from captive amphibians to…

	Not at all	Slightly	Moderately	Very	Extremely
Other amphibians in the live animal trade	☐	☐	☐	☐	☐
Native amphibians	☐	☐	☐	☐	☐

Ranavirus: Known to infect over **200** species

- Ranavirus is one of the **leading causes of death** of amphibians in the United States

[*Images used to illustrate this question are omitted from this document.*]

How **concerned** are you about the spread of ranavirus from captive amphibians and reptiles to…

	Not at all	Slightly	Moderately	Very	Extremely
Other amphibians and reptiles in the live animal trade	☐	☐	☐	☐	☐
Native amphibians and reptiles	☐	☐	☐	☐	☐
Native fish	☐	☐	☐	☐	☐

Loss of biodiversity

Biodiversity is the variety of species in an environment. The loss of one or more species results in decreased biodiversity.

The following shows how the loss of 2 amphibian and reptile species impacts biodiversity.

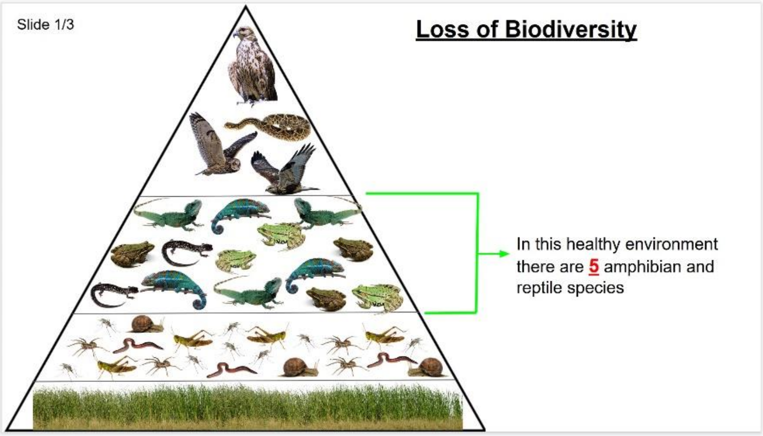

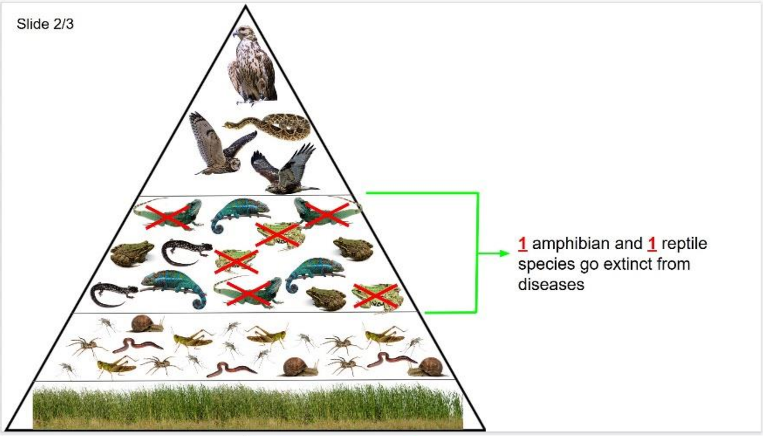

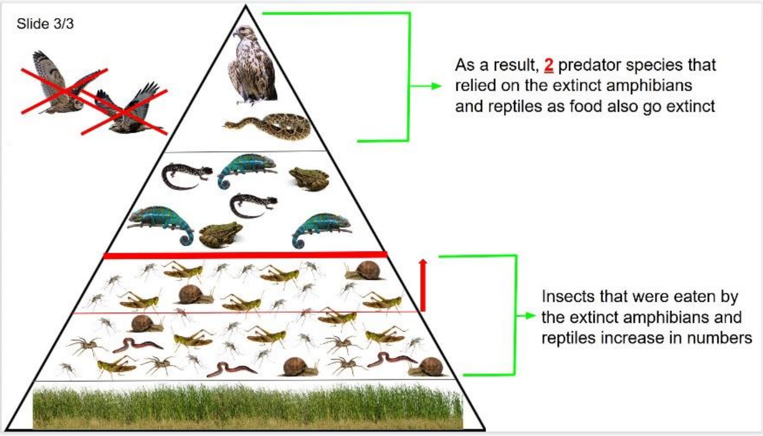

How **concerned** are you about a loss of biodiversity from the disease-related deaths of native amphibians and reptiles?

- Not at all
- Slightly
- Moderately
- Very
- Extremely

What do you think the **risk** is that the diseases discussed in this survey could result in a loss of biodiversity?

- None
- Low
- Moderate
- High
- Very high

Economic Impacts

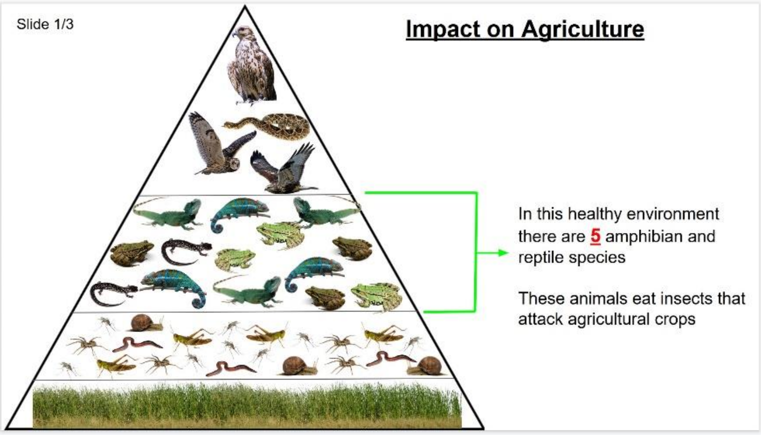

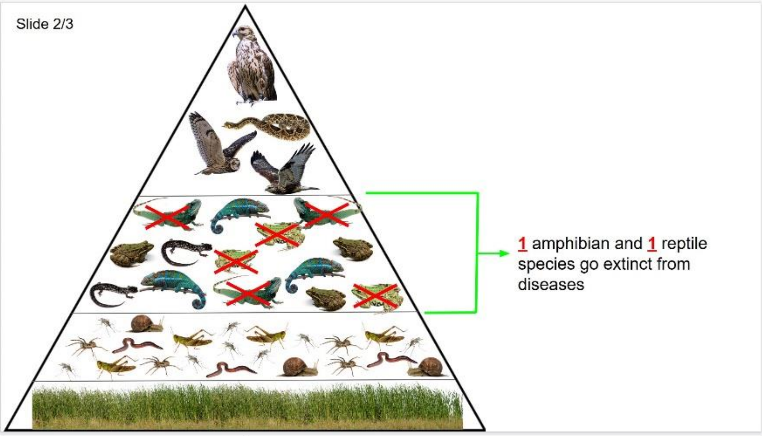

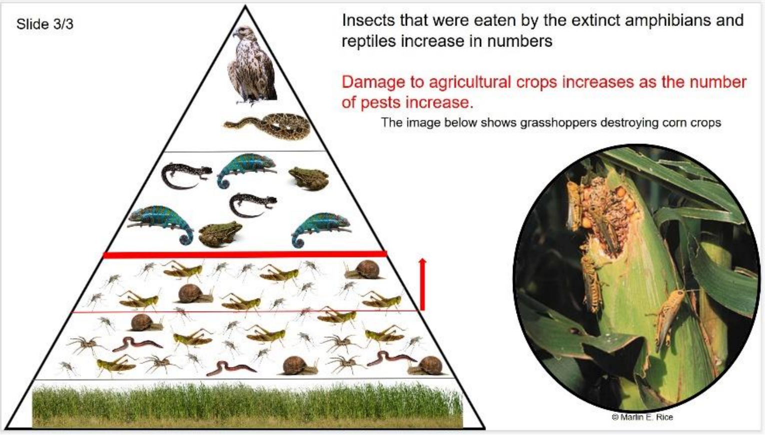

How **concerned** are you about a negative economic impact to agriculture from disease-related deaths of native amphibians and reptiles?

- Not at all
- Slightly
- Moderately
- Very
- Extremely

What do you think the **risk** is that the diseases discussed in this survey could result in a negative economic impact to agriculture?

- None
- Low
- Moderate
- High
- Very high

Threat to aquaculture

Aquaculture is an economically important industry. Ranaviruses can spread from amphibians and reptiles to fish, and kill aquaculture fish.
Aquaculture is the breeding and harvesting of aquatic animals for food.
The United States generates **$1.5 billion** a year from farm-raised fish.
More than 20% of seafood and fish products in the U.S. come from fish farming.
[*Images used to illustrate this question are omitted from this document.*]

How **concerned** are you about a negative economic impact to aquaculture from the disease-related deaths of fish?

- Not at all
- Slightly
- Moderately
- Very
- Extremely

What do you think the **risk** is that ranavirus could result in an negative economic impact to aquaculture?

- None
- Low
- Moderate
- High
- Very high

Threat to the live amphibian and reptile trade

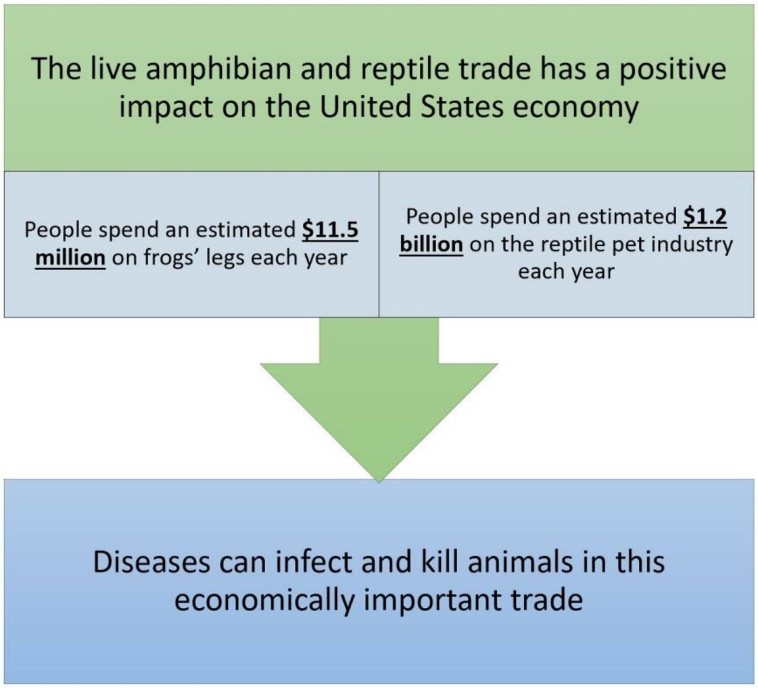

How **concerned** are you about a negative economic impact to the following markets from disease-related deaths of amphibians and reptiles?

	Not at all	Slightly	Moderately	Very	Extremely
The amphibian and reptile pet trade	☐	☐	☐	☐	☐
The frog leg market	☐	☐	☐	☐	☐

What do you think the **risk** is the diseases discussed in this survey could result in a negative economic impact to the following markets?

	None	Low	Moderate	High	Very high
The amphibian and reptile pet trade	☐	☐	☐	☐	☐
The frog leg market	☐	☐	☐	☐	☐

Human Health and Well-being Impacts

Salmonella: **202 people caught salmonella from turtles** between 2015 and 2016.

- Most people recover without treatment, but salmonella can be dangerous for children, older adults, and pregnant women.

[*Images used to illustrate this question are omitted from this document.*]

How **<u>concerned</u>** are you about the <u>spread</u> of salmonella from captive amphibians and reptiles to…

	Not at all	Slightly	Moderately	Very	Extremely
Other amphibians and reptiles in the live animal trade	☐	☐	☐	☐	☐
Native amphibians and reptiles	☐	☐	☐	☐	☐
Pets	☐	☐	☐	☐	☐
Livestock, such as cows, sheep, and goats	☐	☐	☐	☐	☐
Humans	☐	☐	☐	☐	☐

The following demonstrates how the loss of 2 amphibians and reptile species can impact the number of insects.

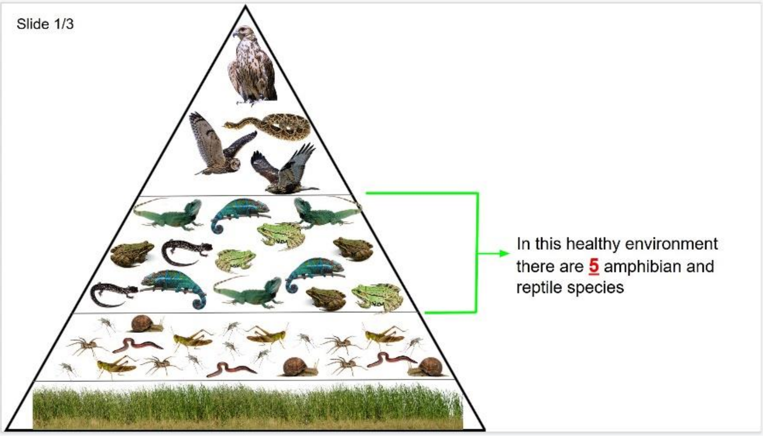

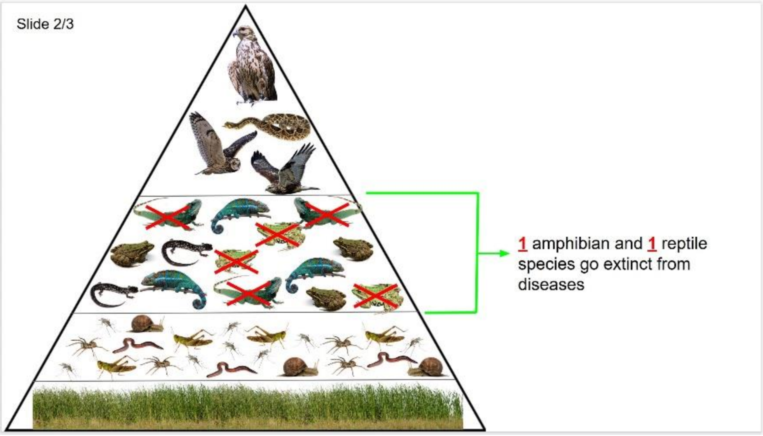

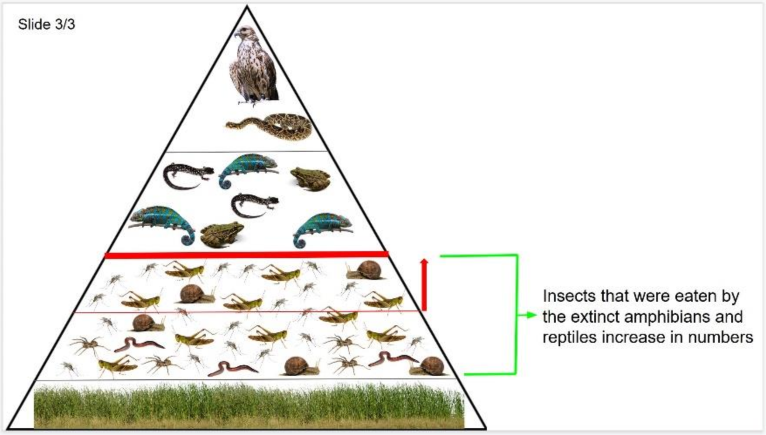

“Pest” control

Amphibians and reptiles eat many insects that humans consider pests (for example, mosquitos, flies, beetles, grasshoppers, and slugs). For example, 1 million frogs eat approximately 7.4 million pounds of insect pests in a year.

Insect pests increase as the numbers of amphibians and reptiles decrease.

How **concerned** are you about an increase in insect pests from the disease-related deaths of native amphibians and reptiles?

- Not at all
- Slightly
- Moderately
- Very
- Extremely

What do you think the **risk** is that the diseases discussed in this survey could result in an increase in insect pests?

- None
- Low
- Moderate
- High
- Very high

Disease control

Amphibians and reptiles eat many insects that carry diseases that affect humans (for example, West Nile virus, malaria, Zika virus, and Lyme disease).

These diseases in humans may increase as the numbers of amphibians and reptiles decrease.

How **concerned** are you about an increase in insect-borne diseases from disease-related deaths of native amphibians and reptiles?

- Not at all
- Slightly
- Moderately
- Very
- Extremely

What do you think the **risk** is that the diseases discussed in this survey could result in an increase in insect-borne diseases?

- None
- Low
- Moderate
- High
- Very high

Management of the Live Amphibian and Reptile Trade

Below are several potential ways to manage the disease transmission risk associated with the trade in amphibians and reptiles. (*Please assume that all of the management actions are equally achievable*.)

Please indicate how much you oppose or support the following potential regulations.

	Strongly oppose	Slightly oppose	Neutral	Slightly favor	Strongly favor
A law that requires the **quarantine and veterinary observation** of all amphibians and reptiles imported into the United States by the federal government.	□	□	□	□	□
**Mandatory tests** by the federal government of all shipments of amphibians and reptiles for selected diseases of concern.	□	□	□	□	□
A mandatory '**Best Practices Program**'. The program would require live amphibian and reptile importers and exporters to follow methods to **improve the care and reduce the stress** of transported animals and **decontaminate** all shipping materials (for example soil and water).	□	□	□	□	□

Government Management of Amphibian and Reptile Diseases

Please indicate how strongly you agree or disagree with the following statements.

The federal government…

	Strongly disagree	Somewhat disagree	Neither agree nor disagree	Somewhat agree	Strongly agree
Has the knowledge to manage the amphibian and reptile disease transmission risk	□	□	□	□	□
Has the money to manage the amphibian and reptile disease transmission risk	□	□	□	□	□
Has sufficient skilled people to manage the amphibian and reptile disease transmission risk	□	□	□	□	□
If you are reading this, please select 'somewhat disagree'	□	□	□	□	□
Has been effective in managing the amphibian and reptile disease transmission risk	□	□	□	□	□
Can be trusted to properly manage the amphibian and reptile disease transmission risk	□	□	□	□	□

Below are some statements about a random person. How similar is this person to you? (*Please take your time to read each statement and answer as honestly as possible*.)

	1 – Not at all like me	2	3	4 – Neutral	5	6	7 – Very much like me
It is important to them to prevent environmental pollution	□	□	□	□	□	□	□
It is important to them that every person has equal opportunities	□	□	□	□	□	□	□
It is important to them to have fun	□	□	□	□	□	□	□
It is important to them to have control over rothers' actions	□	□	□	□	□	□	□
It is important to them to take care of those who are worse off	□	□	□	□	□	□	□
It is important to them to be influential	□	□	□	□	□	□	□
It is important to them to have money and possessions	□	□	□	□	□	□	□

It is important to them to enjoy life's pleasures	□	□	□	□	□	□	□
It is important to them that there is no war or conflict	□	□	□	□	□	□	□
It is important to them to respect nature	□	□	□	□	□	□	□
It is important to them to work hard and be ambitious	□	□	□	□	□	□	□
It is important to them to protect the environment	□	□	□	□	□	□	□
It is important to them to be helpful to others	□	□	□	□	□	□	□
It is important to them to do things they enjoy	□	□	□	□	□	□	□
It is important to them to have authority over others	□	□	□	□	□	□	□
It is important to them to be in unity with nature	□	□	□	□	□	□	□
It is important to them that every person is treated justly	□	□	□	□	□	□	□

Have you participated in the following activities within the past 12 months?

	Yes	No
Hiking	□	□
Camping	□	□
Canoeing/kayaking	□	□
Gardening	□	□
Hunting	□	□
Wildlife viewing	□	□
Going to the zoo	□	□

Now that you have had some time to think about issues related to the live amphibian and reptile trade, how concerned are you about the disease transmission risk associated with the trade?

- Not at all
- Slightly
- Moderately
- Very
- Extremely

I would describe my political views as:

Extremely liberal Moderate Extremely conservative

1 7
