# Supplementary material for: Investigating public support for biosecurity measures to mitigate pathogen transmission through the herpetological trade
Source: PLoS One. 2022 Jan 21;17(1):e0262719. doi: 10.1371/journal.pone.0262719 (PMC8782347; doi:10.1371/journal.pone.0262719)
Supplement: S1 Table — (PDF) [file pone.0262719.s003.pdf]

S1 Table: Survey respondents' demographic characteristics and ownership of domestic animals.

	All respondents		California		Florida		New York		Texas	
	No.	%	No.	%	No.	%	No.	%	No.	%
Gender:										
Male	945	47.1	235	47.2	234	47.0	227	44.8	249	49.4
Female	1,054	52.5	261	52.4	260	52.2	280	55.2	253	50.2
Prefer not to say	8	0.4	2	0.4	4	0.8	0	0.0	2	0.4
Age:										
18-24 years	233	11.6	58	11.7	45	9.0	57	11.2	73	14.5
25-34 years	349	17.4	88	17.7	72	14.5	80	15.8	109	21.6
35-44 years	324	16.1	66	13.3	81	16.3	79	15.6	98	19.4
45-54 years	360	17.9	106	21.3	76	15.3	89	17.6	89	17.7
55-64 years	346	17.2	77	15.5	105	21.1	104	20.5	60	11.9
65-74 years	245	12.2	60	12.1	75	15.1	63	12.4	47	9.3
75 years or over	150	7.5	43	8.6	44	8.8	35	6.9	28	5.6
Education:										
Less than 12th grade	39	1.9	10	2.0	5	1.0	13	2.6	11	2.2
High school graduate or GED	343	17.1	79	15.9	95	19.1	73	14.4	96	19.1
Some college/associate or technical degree	778	38.8	198	39.8	180	36.1	183	36.1	217	43.1
Bachelor's degree	527	26.3	147	29.5	137	27.5	127	25.1	116	23.0
Graduate or professional degree	320	15.9	64	12.9	81	16.3	111	21.9	64	12.7
Race:										
White	1,585	79.0	347	69.7	424	85.1	413	81.5	401	79.6
Black or African American	196	9.8	54	10.8	43	8.6	52	10.3	47	9.3
Asian	100	5.0	57	11.5	10	2.0	17	3.4	16	3.2
American Indian or Alaska Native	58	2.9	18	3.6	14	2.8	8	1.6	18	3.6
Native Hawaiian or Pacific Islander	17	0.9	10	2.0	0	0.0	1	0.2	6	1.2
Other	101	5.0	32	6.4	15	3.0	22	4.3	32	6.4
Prefer not to say	39	1.9	14	2.8	7	1.4	8	1.6	10	2.0
Are you Hispanic and/or Latino?										
Yes	463	23.1	115	23.1	84	16.9	75	14.8	189	37.5
No	1,519	75.7	374	75.1	410	82.3	426	84.0	309	61.3
Prefer not to say	25	1.3	9	1.8	4	0.8	6	1.2	6	1.2
Household contains members under 18 years	607	30.2	143	28.7	135	27.1	140	27.6	189	37.5
Household currently owns pets	1,338	66.7	325	65.3	324	65.1	326	64.3	363	72.0
Type of pets:										

Dog	990	49.3	239	48.0	251	50.4	217	42.8	283	56.2
Cat	667	33.2	157	31.5	152	30.5	196	38.7	162	32.1
Fish	142	7.1	27	5.4	31	6.2	50	9.9	34	6.8
Bird	75	3.7	17	3.4	17	3.4	21	4.1	20	4.0
Reptile (e.g., snake, lizard, turtle, tortoise)	64	3.2	12	2.4	15	3.0	18	3.6	19	3.8
Rodent or small mammal	40	2.0	8	1.6	12	2.4	12	2.4	8	1.6
Amphibian (e.g., frog, toad, salamander, newt)	10	0.5	1	0.2	1	0.2	5	1.0	3	0.6
Insect or arachnid	2	0.1	0	0.0	1	0.2	1	0.2	0	0.0
Other	19	1.0	5	1.0	2	0.4	6	1.2	6	1.2
Household owns livestock	43	2.1	8	1.6	6	1.2	14	2.8	15	3.0
Household owns poultry	39	1.9	10	2.0	10	2.0	10	2.0	9	1.8
Household owns livestock and poultry	47	2.3	10	2.0	10	2.0	14	2.8	13	2.6
