# Supplementary material for: Investigating public support for biosecurity measures to mitigate pathogen transmission through the herpetological trade
Source: PLoS One. 2022 Jan 21;17(1):e0262719. doi: 10.1371/journal.pone.0262719 (PMC8782347; doi:10.1371/journal.pone.0262719)
Supplement: S2 Table — (PDF) [file pone.0262719.s004.pdf]

S2 Table. Confirmatory factor analysis for respondents' support for biosecurity for different survey versions that presented the ecological impacts, economic impacts, human health and wellbeing impacts, or all impacts of pathogen transmission.

	Ecological impacts survey version		Economic impacts survey version		Human health and wellbeing impacts survey version		All impacts survey version	
	Coeff.[†]	Cronbach's alpha[‡]	Coeff.	Cronbach's alpha	Coeff.	Cronbach's alpha	Coeff.	Cronbach's alpha
Loadings:								
x1: A law that requires the quarantine and veterinary observation of all amphibians and reptiles imported into the United States	0.76***	0.788	0.85***	0.756	0.78***	0.756	0.80***	0.791
x2: Mandatory tests of all shipments of amphibians and reptiles for selected diseases of concern	0.88***	0.722	0.88***	0.733	0.88***	0.699	0.92***	0.725
x3: A mandatory 'Best Practices Program' requiring live amphibian and reptile importers and exporters to improve the care and reduce the stress of transported animals and decontaminate all shipping materials	0.74***	0.804	0.70***	0.850	0.71***	0.816	0.72***	0.851
Variances:								
error.x1	0.42		0.28		0.39		0.35	
error.x2	0.23		0.23		0.23		0.15	
error.x3	0.45		0.51		0.50		0.49	
Support for biosecurity	1.00		1.00		1.00		1.00	
N	507		507		505		488	
RMSEA	<0.001		<0.001		<0.001		<0.001	
CFI	1.000		1.000		1.000		1.000	
Cronbach's alpha for scale		0.836		0.846		0.828		0.852

[†] Standardized values. *** denotes significance at p<0.01. ** denotes significance at p<0.05. * denotes significance at p<0.1.

[‡] Cronbach's alpha if items are removed from the scale.
