# Supplementary material for: Investigating public support for biosecurity measures to mitigate pathogen transmission through the herpetological trade
Source: PLoS One. 2022 Jan 21;17(1):e0262719. doi: 10.1371/journal.pone.0262719 (PMC8782347; doi:10.1371/journal.pone.0262719)
Supplement: S3 Table — (PDF) [file pone.0262719.s005.pdf]

S3 Table. Distribution of respondents' prior knowledge of reasons for herpetological imports (n=2,007).

	Median	Percent of respondents			
		Not at all	Slightly	Moderately	Highly
Awareness that live frogs are imported into the United States for human consumption	Slightly	46.0	28.0	19.2	6.9
Awareness that amphibians are imported for use as fishing bait	Slightly	47.4	26.5	18.0	8.0
Awareness that amphibians and reptiles are imported to supply the pet industry	Slightly	23.4	30.5	30.5	15.6
