# Supplementary material for: Investigating public support for biosecurity measures to mitigate pathogen transmission through the herpetological trade
Source: PLoS One. 2022 Jan 21;17(1):e0262719. doi: 10.1371/journal.pone.0262719 (PMC8782347; doi:10.1371/journal.pone.0262719)
Supplement: S4 Table — (PDF) [file pone.0262719.s006.pdf]

S4 Table. Confirmatory factor analysis for respondents' prior knowledge of reasons for herpetological imports ('knowledge of herpetological imports') for different survey versions that presented the ecological impacts, economic impacts, human health and wellbeing impacts, or all impacts of pathogen transmission.

	Ecological impacts survey version		Economic impacts survey version		Human health and wellbeing impacts survey version		All impacts survey version	
	Coeff.†	Cronbach's alpha‡	Coeff.	Cronbach's alpha	Coeff.	Cronbach's alpha	Coeff.	Cronbach's alpha
Loadings:								
x1: Awareness that live frogs are imported into the United States for human consumption	0.69***	0.593	0.77***	0.609	0.70***	0.630	0.71***	0.651
x2: Awareness that amphibians are imported for use as fishing bait	0.75***	0.557	0.67***	0.665	0.69***	0.631	0.72***	0.642
x3: Awareness that amphibians and reptiles are imported to supply the pet industry	0.56***	0.686	0.65***	0.681	0.67***	0.650	0.67***	0.677
Variances:								
error.x1	0.52		0.41		0.52		0.50	
error.x2	0.43		0.55		0.52		0.48	
error.x3	0.69		0.58		0.56		0.55	
Knowledge of herpetological imports	1.00		1.00		1.00		1.00	
N	507		507		505		488	
RMSEA	<0.001		<0.001		<0.001		<0.001	
CFI	1.000		1.000		1.000		1.000	
Cronbach's alpha for scale		0.704		0.738		0.724		0.742

† Standardized values. *** denotes significance at $p<0.01$. ** denotes significance at $p<0.05$. * denotes significance at $p<0.1$.

‡ Cronbach's alpha if items are removed from the scale.
