# Supplementary material for: Investigating public support for biosecurity measures to mitigate pathogen transmission through the herpetological trade
Source: PLoS One. 2022 Jan 21;17(1):e0262719. doi: 10.1371/journal.pone.0262719 (PMC8782347; doi:10.1371/journal.pone.0262719)
Supplement: S5 Table — (PDF) [file pone.0262719.s007.pdf]

S5 Table. Distribution of responses to the question "How much do you like or dislike the following animals?" (n=2,007).

	Median	Percent of respondents				
		Strongly dislike	Dislike	Neither like nor dislike	Like	Strongly like
Snakes	Dislike	25.6	26.0	23.4	17.6	7.4
Lizards	Neither like nor dislike	6.0	15.6	31.6	32.8	14.1
Turtles/tortoises	Like	0.9	2.3	19.5	48.0	29.2
Frogs	Neither like nor dislike	4.4	12.6	35.8	34.9	12.4
Toads	Neither like nor dislike	5.5	16.2	40.4	28.4	9.6
Salamanders/newts	Neither like nor dislike	5.5	14.1	40.6	28.6	11.3
Freshwater fish	Like	0.9	1.6	23.5	47.9	26.1
Saltwater fish	Like	1.1	2.1	26.3	45.6	24.9
