# Supplementary material for: Investigating public support for biosecurity measures to mitigate pathogen transmission through the herpetological trade
Source: PLoS One. 2022 Jan 21;17(1):e0262719. doi: 10.1371/journal.pone.0262719 (PMC8782347; doi:10.1371/journal.pone.0262719)
Supplement: S6 Table — (PDF) [file pone.0262719.s008.pdf]

S6 Table. Confirmatory factor analysis for respondents' 'attitudes towards herpetofauna' for different survey versions that presented the ecological impacts, economic impacts, human health and wellbeing impacts, or all impacts of pathogen transmission.

	Ecological impacts survey version		Economic impacts survey version		Human health and wellbeing impacts survey version		All impacts survey version	
	Coeff.†	Cronbach's alpha‡	Coeff.	Cronbach's alpha	Coeff.	Cronbach's alpha	Coeff.	Cronbach's alpha
Loadings:								
x1: Snakes	0.65***	0.899	0.66***	0.904	0.69***	0.868	0.66***	0.891
x2: Lizards	0.90***	0.854	0.86***	0.874	0.82***	0.837	0.85***	0.857
x3: Turtles/tortoises	0.65***	0.889	0.58***	0.910	0.57***	0.876	0.57***	0.894
x4: Frogs	0.80***	0.863	0.85***	0.871	0.76***	0.834	0.78***	0.863
x5: Toads	0.77***	0.869	0.82***	0.876	0.75***	0.835	0.81***	0.862
x6: Salamanders/newts	0.85***	0.863	0.89***	0.874	0.80***	0.837	0.88***	0.860
Variances:								
error.x1	0.57		0.56		0.53		0.56	
error.x2	0.19		0.27		0.33		0.28	
error.x3	0.58		0.66		0.67		0.68	
error.x4	0.35		0.27		0.42		0.40	
error.x5	0.41		0.32		0.44		0.35	
error.x6	0.28		0.21		0.36		0.23	
Attitudes towards herpetofauna	1.00		1.00		1.00		1.00	
Covariance:								
error.x1 with error.x2			0.26***				0.24***	
error.x1 with error.x3					-0.28***			
error.x3 with error.x4							0.16***	
error.x4 with error.x5	0.45***		0.58***		0.55***		0.53***	
N	507		507		505		488	
RMSEA	0.042		0.020		0.034		0.041	
CFI	0.973		0.996		0.987		0.985	
χ^2	15.174*		8.481		11.138		10.858*	
Cronbach's alpha for scale		0.892		0.903		0.871		0.891

† Standardized values. *** denotes significance at p<0.01. ** denotes significance at p<0.05. * denotes significance at p<0.1.

‡ Cronbach's alpha if items are removed from the scale.
