# Supplementary material for: Investigating public support for biosecurity measures to mitigate pathogen transmission through the herpetological trade
Source: PLoS One. 2022 Jan 21;17(1):e0262719. doi: 10.1371/journal.pone.0262719 (PMC8782347; doi:10.1371/journal.pone.0262719)
Supplement: S7 Table — (PDF) [file pone.0262719.s009.pdf]

S7 Table. Distribution of responses to the questions used to measure respondents' sensitivity to general health risks (n=2,007).

	Median	Percent of respondents				
		Not at all	Slightly	Moderately	Very	Extremely
Please rate how important it is to you to protect the health of…						
Animals in the live animal trade	Very	2.3	3.4	13.9	32.5	47.9
Native wildlife	Extremely	1.3	2.6	10.0	29.3	56.8
The natural environment	Extremely	0.8	1.6	9.7	29.9	58.0
Pets	Extremely	0.8	1.5	7.3	28.7	61.7
Livestock, such as cows, sheep, and goats	Very	1.7	2.6	14.2	33.1	48.4
Humans	Extremely	1.2	1.4	6.3	20.2	70.9
