# Supplementary material for: Investigating public support for biosecurity measures to mitigate pathogen transmission through the herpetological trade
Source: PLoS One. 2022 Jan 21;17(1):e0262719. doi: 10.1371/journal.pone.0262719 (PMC8782347; doi:10.1371/journal.pone.0262719)
Supplement: S8 Table — (PDF) [file pone.0262719.s010.pdf]

S8 Table. Confirmatory factor analysis for the importance that respondents placed on protecting the health of animals in the live animal trade, native wildlife, the natural environment, pets, and livestock ('sensitivity to general health risks') for different survey versions that presented the ecological impacts, economic impacts, human health and wellbeing impacts, or all impacts of pathogen transmission.

	Ecological impacts survey version		Economic impacts survey version		Human health and wellbeing impacts survey version		All impacts survey version	
	Coeff.†	Cronbach's alpha‡	Coeff.	Cronbach's alpha	Coeff.	Cronbach's alpha	Coeff.	Cronbach's alpha
Loadings:								
x1: Animals in the live animal trade	0.85***	0.862	0.87***	0.863	0.87***	0.851	0.90***	0.874
x2: Native wildlife	0.74***	0.856	0.76***	0.858	0.79***	0.849	0.81***	0.872
x3: The natural environment	0.71***	0.865	0.74***	0.868	0.73***	0.857	0.75***	0.884
x4: Pets	0.76***	0.876	0.73***	0.880	0.69***	0.879	0.68***	0.906
x5: Livestock	0.78***	0.872	0.82***	0.867	0.78***	0.866	0.86***	0.881
Variances:								
error.x1	0.28		0.24		0.23		0.19	
error.x2	0.45		0.43		0.38		0.34	
error.x3	0.50		0.46		0.47		0.44	
error.x4	0.43		0.46		0.53		0.54	
error.x5	0.39		0.32		0.39		0.26	
Sensitivity to general health risks	1.00		1.00		1.00		1.00	
Covariance:								
error.x1 with error.x3			-0.23***					
error.x2 with error.x3	0.58***		0.56***		0.52***		0.51***	
N	507		507		505		488	
RMSEA	0.050		<0.001		0.049		0.031	
CFI	0.954		1.000		0.966		0.986	
χ^2	9.533**		2.545		8.871*		5.862	
Cronbach's alpha for scale		0.890		0.891		0.886		0.905

† Standardized values. *** denotes significance at p<0.01. ** denotes significance at p<0.05. * denotes significance at p<0.1.

‡ Cronbach's alpha if items are removed from the scale.
