# Supplementary material for: Investigating public support for biosecurity measures to mitigate pathogen transmission through the herpetological trade
Source: PLoS One. 2022 Jan 21;17(1):e0262719. doi: 10.1371/journal.pone.0262719 (PMC8782347; doi:10.1371/journal.pone.0262719)
Supplement: S9 Table — (PDF) [file pone.0262719.s011.pdf]

S9 Table. Distribution of responses to the questions used to measure respondents' sensitivity to herpetological trade risks (n=2,007).

	Median	Percent of respondents				
		Not at all	Slightly	Moderately	Very	Extremely
How concerned are you about disease transmission from captive amphibians and reptiles to…						
Other captive amphibians	Very	4.9	12.4	30.3	30.3	22.1
Native wildlife	Very	3.3	9.3	24.7	32.5	30.1
Pets	Very	4.1	9.0	20.2	31.4	35.3
Livestock, such as cows, sheep, and goats	Very	3.3	8.3	21.9	33.0	33.5
Humans	Very	3.9	6.7	16.8	29.5	43.0
