# Supplementary material for: Investigating public support for biosecurity measures to mitigate pathogen transmission through the herpetological trade
Source: PLoS One. 2022 Jan 21;17(1):e0262719. doi: 10.1371/journal.pone.0262719 (PMC8782347; doi:10.1371/journal.pone.0262719)
Supplement: S10 Table — (PDF) [file pone.0262719.s012.pdf]

S10 Table. Confirmatory factor analysis for respondents' level of concern about pathogen transmission from captive herpetofauna to other captive animals, native wildlife, pets, livestock, and humans ('sensitivity to herpetological trade risk') for different survey versions that presented the ecological impacts, economic impacts, human health and wellbeing impacts, or all impacts of pathogen transmission.

	Ecological impacts survey version		Economic impacts survey version		Human health and wellbeing impacts survey version		All impacts survey version	
	Coeff.†	Cronbach's alpha‡	Coeff.	Cronbach's alpha	Coeff.	Cronbach's alpha	Coeff.	Cronbach's alpha
Loadings:								
x1: Other captive amphibians	0.76***	0.887	0.74***	0.877	0.74***	0.872	0.77***	0.904
x2: Native wildlife	0.86***	0.869	0.81***	0.862	0.80***	0.860	0.83***	0.895
x3: Pets	0.89***	0.867	0.90***	0.860	0.89***	0.858	0.84***	0.896
x4: Livestock	0.88***	0.860	0.88***	0.856	0.83***	0.860	0.91***	0.888
x5: Humans	0.63***	0.913	0.60***	0.911	0.64***	0.897	0.69***	0.915
Variances:								
error.x1	0.42		0.46		0.46		0.41	
error.x2	0.26		0.35		0.35		0.31	
error.x3	0.20		0.19		0.21		0.30	
error.x4	0.23		0.22		0.31		0.18	
error.x5	0.61		0.64		0.59		0.52	
Sensitivity to herpetological trade risk	1.00		1.00		1.00		1.00	
Covariance:								
error.x1 with error.x2	0.36***		0.48***		0.43***		0.54***	
error.x3 with error.x5							0.35***	
error.x4 with error.x5	0.31***		0.21***		0.24***		0.32***	
N	507		507		505		488	
RMSEA	0.043		0.026		<0.001		<0.001	
CFI	0.971		0.997		1.000		1.000	
χ^2	11.175**		4.007		2.810		1.300	
Cronbach's alpha for scale		0.902		0.897		0.893		0.918

† Standardized values. *** denotes significance at p<0.01. ** denotes significance at p<0.05. * denotes significance at p<0.1.
‡ Cronbach's alpha if items are removed from the scale.
