# Supplementary material for: Investigating public support for biosecurity measures to mitigate pathogen transmission through the herpetological trade
Source: PLoS One. 2022 Jan 21;17(1):e0262719. doi: 10.1371/journal.pone.0262719 (PMC8782347; doi:10.1371/journal.pone.0262719)
Supplement: S11 Table — (PDF) [file pone.0262719.s013.pdf]

S11 Table. Distribution of respondents' risk perceptions pertaining to transmission of *Bd*, *Bsal*, ranaviruses and *Salmonella* by captive herpetofauna (n=2,007).

	Median	Percent of respondents				
		None	Low	Moderate	High	Very high
What do you think the risk is that chytrid would be transmitted from captive amphibians to...						
Other captive amphibians	High	0.9	5.0	24.0	35.9	34.1
Native amphibians	High	0.7	7.9	29.0	35.4	26.9
What do you think the risk is that ranavirus would be transmitted from captive amphibians and reptiles to...						
Other captive amphibians and reptiles	High	0.8	5.5	24.7	36.9	32.1
Native amphibians and reptiles	High	0.8	6.8	28.8	36.7	26.8
Native fish	High	0.9	7.7	28.1	36.4	27.0
What do you think the risk is that salmonella would be transmitted from captive amphibians and reptiles to...						
Other captive amphibians and reptiles	High	1.4	6.5	26.4	38.4	27.3
Native amphibians and reptiles	High	1.7	7.9	29.2	37.7	23.5
Pets	High	1.4	13.2	29.4	33.4	22.5
Livestock, such as cows, sheep, and goats	High	1.3	12.6	29.3	33.5	23.3
Humans	High	1.1	9.8	21.6	32.9	34.6
