# Supplementary material for: Investigating public support for biosecurity measures to mitigate pathogen transmission through the herpetological trade
Source: PLoS One. 2022 Jan 21;17(1):e0262719. doi: 10.1371/journal.pone.0262719 (PMC8782347; doi:10.1371/journal.pone.0262719)
Supplement: S12 Table — (PDF) [file pone.0262719.s014.pdf]

S12 Table. Confirmatory factor analysis for respondents' 'susceptibility to herpetological pathogen transmission' for different survey versions that presented the ecological impacts, economic impacts, human health and wellbeing impacts, or all impacts of pathogen transmission.

	Ecological impacts survey version		Economic impacts survey version		Human health and wellbeing impacts survey version		All impacts survey version	
	Coeff.[†]	Cronbach's alpha[‡]	Coeff.	Cronbach's alpha	Coeff.	Cronbach's alpha	Coeff.	Cronbach's alpha
Loadings:								
x1: Chytrid transmitted to other captive amphibians	0.73***	0.922	0.72***	0.930	0.66***	0.919	0.75***	0.928
x2: Chytrid transmitted to native amphibians	0.83***	0.920	0.77***	0.930	0.78***	0.918	0.81***	0.925
x3: Ranavirus transmitted to other captive amphibians and reptiles	0.72***	0.921	0.79***	0.927	0.70***	0.917	0.93***	0.926
x4: Ranavirus transmitted to native amphibians and reptiles	0.83***	0.918	0.89***	0.925	0.84***	0.914	0.84***	0.922
x5: Ranavirus transmitted to native fish	0.77***	0.921	0.87***	0.927	0.81***	0.915	0.79***	0.924
x6: Salmonella transmitted to other captive amphibians and reptiles	0.77***	0.921	0.77***	0.929	0.88***	0.916	0.74***	0.926
x7: Salmonella transmitted to native amphibians and reptiles	0.88***	0.917	0.87***	0.927	0.77***	0.913	0.87***	0.923
x8: Salmonella transmitted to pets	0.69***	0.922	0.74***	0.929	0.70***	0.915	0.73***	0.925
x9: Salmonella transmitted to livestock	0.66***	0.923	0.74***	0.930	0.64***	0.918	0.69***	0.928
x10: Salmonella transmitted to humans	0.69***	0.924	0.71***	0.931	0.61***	0.923	0.66***	0.930
Variances:								
error.x1	0.46		0.48		0.56		0.44	
error.x2	0.32		0.40		0.39		0.35	
error.x3	0.48		0.38		0.51		0.14	
error.x4	0.31		0.20		0.29		0.29	
error.x5	0.40		0.25		0.34		0.37	
error.x6	0.41		0.41		0.22		0.45	
error.x7	0.22		0.25		0.41		0.24	

error.x8	0.52	0.45	0.51	0.47
error.x9	0.57	0.45	0.59	0.52
error.x10	0.53	0.50	0.63	0.57
Susceptibility to herpetological pathogen transmission	1.00	1.00	1.00	1.00
Covariance:				
error.x1 with error.x2	0.38***	0.46***	0.28***	0.31***
error.x1 with error.x3	0.35***	0.47***	0.33***	
error.x1 with error.x6	0.32***	0.34***		0.28***
error.x1 with error.x7				-0.24***
error.x2 with error.x3				-0.70***
error.x2 with error.x6			-0.82***	
error.x2 with error.x9		0.10***		
error.x3 with error.x4	0.54***		0.33***	
error.x3 with error.x5	0.35***	0.26***	0.23***	
error.x3 with error.x6	0.30***	0.32***		
error.x3 with error.x7				-1.16***
error.x3 with error.x8				-0.48***
error.x3 with error.x9		-0.08**		-0.71***
error.x3 with error.x10				-0.55***
error.x4 with error.x5	0.56***	0.46***	0.43***	0.63***
error.x4 with error.x6			-0.83***	
error.x4 with error.x8			-0.16***	
error.x4 with error.x9				-0.09**
error.x4 with error.x10			-0.12**	
error.x5 with error.x6			-0.74***	
error.x5 with error.x8			-0.10*	
error.x6 with error.x7	0.35***	0.41***	0.24***	0.30***
error.x6 with error.x10		0.07*	-0.20**	
error.x7 with error.x8			0.33***	
error.x7 with error.x9	0.17***		0.28***	
error.x8 with error.x9	0.53***	0.43***	0.59***	0.46***
error.x8 with error.x10	0.47***	0.44***	0.35***	0.45***
error.x9 with error.x10	0.41***	0.44***	0.35***	0.32***
N	507	507	505	488
RMSEA	0.050	0.044	0.016	0.050

CFI	0.934		0.971		0.997		0.954	
χ^2	54.633***		41.981***		18.097		47.964***	
Cronbach's alpha for scale		0.928		0.935		0.925		0.933

† Standardized values. *** denotes significance at $p<0.01$. ** denotes significance at $p<0.05$. * denotes significance at $p<0.1$.

‡ Cronbach's alpha if items are removed from the scale.
