# Supplementary material for: Investigating public support for biosecurity measures to mitigate pathogen transmission through the herpetological trade
Source: PLoS One. 2022 Jan 21;17(1):e0262719. doi: 10.1371/journal.pone.0262719 (PMC8782347; doi:10.1371/journal.pone.0262719)
Supplement: S13 Table — (PDF) [file pone.0262719.s015.pdf]

S13 Table. Distribution of respondents' risk concerns about the ecological impacts of pathogen transmission through the herpetological trade (n=995).

	Median	Percent of respondents				
		Not at all	Slightly	Moderately	Very	Extremely
How concerned are you about the spread of chytrid from captive amphibians to...						
Other amphibians in the live animal trade	Very	4.0	8.5	24.7	35.3	27.4
Native amphibians	Very	3.2	9.0	24.2	34.1	29.4
How concerned are you about the spread of ranaviruses from captive amphibians and reptiles to...						
Other amphibians and reptiles in the live animal trade	Very	3.3	8.5	24.5	35.8	27.8
Native amphibians and reptiles	Very	3.2	8.2	23.8	35.3	29.4
Native fish	Very	3.9	8.6	21.9	35.3	30.3
How concerned are you about the loss of biodiversity from the disease-related deaths of native amphibians and reptiles	Very	3.2	7.9	20.5	36.3	32.1
