# Supplementary material for: Investigating public support for biosecurity measures to mitigate pathogen transmission through the herpetological trade
Source: PLoS One. 2022 Jan 21;17(1):e0262719. doi: 10.1371/journal.pone.0262719 (PMC8782347; doi:10.1371/journal.pone.0262719)
Supplement: S14 Table — (PDF) [file pone.0262719.s016.pdf]

S14 Table. Confirmatory factor analysis for respondents' concern pertaining to ecological impacts of pathogen transmission through the herpetological trade ('sensitivity to ecological risks').

	Ecological impacts survey version		All impacts survey version	
	Coeff.†	Cronbach's alpha‡	Coeff.	Cronbach's alpha
Loadings:				
x1: Chytrid transmitted to other captive amphibians	0.87***	0.941	0.87***	0.947
x2: Chytrid transmitted to native amphibians	0.90***	0.941	0.89***	0.947
x3: Ranavirus transmitted to other captive amphibians and reptiles	0.87***	0.942	0.90***	0.947
x4: Ranavirus transmitted to native amphibians and reptiles	0.95***	0.939	0.96***	0.943
x5: Ranavirus transmitted to native fish	0.89***	0.946	0.91***	0.947
x6: Loss of biodiversity	0.79***	0.956	0.80***	0.958
Variances:				
error.x1	0.24		0.25	
error.x2	0.20		0.20	
error.x3	0.24		0.20	
error.x4	0.11		0.08	
error.x5	0.21		0.17	
error.x6	0.37		0.36	
Sensitivity to ecological risks	1.00		1.00	
Covariance:				
error.x1 with error.x2	0.41***		0.36***	
error.x1 with error.x3	0.40***		0.48***	
error.x3 with error.x4	0.42***			
error.x4 with error.x6	-0.25***			
N	507		488	
RMSEA	0.039		0.034	
CFI	0.988		0.985	
χ^2	8.758		10.903	
Cronbach's alpha for scale		0.953		0.956

† Standardized values. *** denotes significance at p<0.01. ** denotes significance at p<0.05. * denotes significance at p<0.1.

‡ Cronbach's alpha if items are removed from the scale.
