# Supplementary material for: Investigating public support for biosecurity measures to mitigate pathogen transmission through the herpetological trade
Source: PLoS One. 2022 Jan 21;17(1):e0262719. doi: 10.1371/journal.pone.0262719 (PMC8782347; doi:10.1371/journal.pone.0262719)
Supplement: S15 Table — (PDF) [file pone.0262719.s017.pdf]

S15 Table. Distribution of respondents' risk concerns about the economic impacts of pathogen transmission through the herpetological trade (n=995).

	Median	Percent of respondents				
		Not at all	Slightly	Moderately	Very	Extremely
How concerned are you about a negative economic impact to [industry] from disease-related deaths of native amphibians and reptiles?						
Agriculture	Very	2.0	6.4	23.2	39.0	29.3
Aquaculture	Very	1.3	8.4	23.0	39.2	28.0
The amphibian and reptile pet trade	Moderately	7.1	12.9	30.3	30.8	19.0
The frog leg market	Moderately	12.4	16.9	27.4	25.3	18.0
