# Supplementary material for: Investigating public support for biosecurity measures to mitigate pathogen transmission through the herpetological trade
Source: PLoS One. 2022 Jan 21;17(1):e0262719. doi: 10.1371/journal.pone.0262719 (PMC8782347; doi:10.1371/journal.pone.0262719)
Supplement: S16 Table — (PDF) [file pone.0262719.s018.pdf]

S16 Table. Confirmatory factor analysis for respondents' concern pertaining to economic impacts of pathogen transmission through the herpetological trade ('sensitivity to economic risks').

	Economic impacts survey version		All impacts survey version	
	Coeff.[†]	Cronbach's alpha[‡]	Coeff.	Cronbach's alpha
Loadings:				
x1: Agriculture	0.60***	0.837	0.55***	0.815
x2: Aquaculture	0.64***	0.829	0.60***	0.796
x3: Amphibian and reptile trade	0.94***	0.784	0.97***	0.752
x4: Frog leg market	0.83***	0.823	0.77***	0.816
Variances:				
error.x1	0.64		0.69	
error.x2	0.59		0.64	
error.x3	0.11		0.06	
error.x4	0.32		0.41	
Sensitivity to economic risks	1.00		1.00	
Covariance:				
error.x1 with error.x2	0.52***		0.57***	
N	507		488	
RMSEA	0.030		0.012	
CFI	0.999		1.000	
χ^2	1.445		1.066	
Cronbach's alpha for scale		0.858		0.838

[†] Standardized values. *** denotes significance at $p<0.01$. ** denotes significance at $p<0.05$. * denotes significance at $p<0.1$.

[‡] Cronbach's alpha if items are removed from the scale.
