# Supplementary material for: Investigating public support for biosecurity measures to mitigate pathogen transmission through the herpetological trade
Source: PLoS One. 2022 Jan 21;17(1):e0262719. doi: 10.1371/journal.pone.0262719 (PMC8782347; doi:10.1371/journal.pone.0262719)
Supplement: S17 Table — (PDF) [file pone.0262719.s019.pdf]

S17 Table. Distribution of respondents' risk perceptions related to the economic impacts of pathogen transmission through the live herpetological trade (n=995).

	Median	Percent of respondents				
		None	Low	Moderate	High	Very high
What do you think the risk is that the diseases discussed in this survey could result in a negative economic impact to…						
Agriculture	High	1.0	4.7	23.3	38.9	32.1
Aquaculture	High	0.9	6.2	24.2	38.9	29.7
The amphibian and reptile pet trade	High	2.1	10.3	30.8	33.6	23.3
The frog leg market	High	3.7	11.8	29.3	32.2	23.0
