# Supplementary material for: Investigating public support for biosecurity measures to mitigate pathogen transmission through the herpetological trade
Source: PLoS One. 2022 Jan 21;17(1):e0262719. doi: 10.1371/journal.pone.0262719 (PMC8782347; doi:10.1371/journal.pone.0262719)
Supplement: S18 Table — (PDF) [file pone.0262719.s020.pdf]

S18 Table. Confirmatory factor analysis for respondents' perceived 'susceptibility to economic risks' associated with pathogen transmission through the live herpetological trade.

	Economic impacts survey version		All impacts survey version	
	Coeff.†	Cronbach's alpha‡	Coeff.	Cronbach's alpha
Loadings:				
x1: Agriculture	0.56***	0.857	0.68	0.878
x2: Aquaculture	0.66***	0.828	0.73	0.858
x3: Amphibian and reptile trade	0.92***	0.813	0.95	0.844
x4: Frog leg market	0.88***	0.823	0.86	0.871
Variances:				
error.x1	0.68		0.54	
error.x2	0.57		0.47	
error.x3	0.16		0.10	
error.x4	0.22		0.26	
Susceptibility to economic risks	1.00		1.00	
Covariance:				
error.x1 with error.x2	0.57***		0.53	
N	507		488	
RMSEA	0.043		0.050	
CFI	0.997		0.988	
χ^2	1.948		3.701*	
Cronbach's alpha for scale		0.868		0.894

† Standardized values. *** denotes significance at p<0.01. ** denotes significance at p<0.05. * denotes significance at p<0.1.

‡ Cronbach's alpha if items are removed from the scale.
