# Supplementary material for: Investigating public support for biosecurity measures to mitigate pathogen transmission through the herpetological trade
Source: PLoS One. 2022 Jan 21;17(1):e0262719. doi: 10.1371/journal.pone.0262719 (PMC8782347; doi:10.1371/journal.pone.0262719)
Supplement: S19 Table — (PDF) [file pone.0262719.s021.pdf]

S19 Table. Distribution of respondents' concerns about the human health and wellbeing impacts of pathogen transmission through the live herpetological trade (n=993).

	Median	Percent of respondents				
		Not at all	Slightly	Moderately	Very	Extremely
How concerned are you about the spread of salmonella from captive amphibians and reptiles to...						
Other amphibians and reptiles in the live animal trade	Very	3.3	11.4	27.9	32.2	25.2
Native amphibians and reptiles	Very	3.1	12.4	28.4	33.2	22.9
Pets	Very	3.7	11.6	22.9	32.2	29.6
Livestock, such as cows, sheep, and goats	Very	4.3	10.8	23.5	33.9	27.5
Humans	Very	3.8	7.8	16.6	29.0	42.8
