# Supplementary material for: Investigating public support for biosecurity measures to mitigate pathogen transmission through the herpetological trade
Source: PLoS One. 2022 Jan 21;17(1):e0262719. doi: 10.1371/journal.pone.0262719 (PMC8782347; doi:10.1371/journal.pone.0262719)
Supplement: S20 Table — (PDF) [file pone.0262719.s022.pdf]

S20 Table. Confirmatory factor analysis for respondents' concern pertaining to human health and wellbeing impacts of pathogen transmission through the live herpetological trade ('sensitivity to human health and wellbeing risks').

	Human health and wellbeing impacts survey version		All impacts survey version	
	Coeff.[†]	Cronbach's alpha[‡]	Coeff.	Cronbach's alpha
Loadings:				
x1: Salmonella transmitted to other captive amphibians	0.76***	0.888	0.88***	0.922
x2: Salmonella transmitted to native amphibians	0.82***	0.882	0.87***	0.923
x3: Salmonella transmitted to pets	0.88***	0.883	0.86***	0.926
x4: Salmonella transmitted to livestock	0.88***	0.884	0.90***	0.924
x5: Salmonella transmitted to humans	0.78***	0.895	0.83***	0.930
x6: Increase in insect pests	0.55***	0.905	0.75***	0.932
x7: Increase in insect-borne diseases	0.64***	0.897	0.74***	0.931
Variances:				
error.x1	0.42		0.22	
error.x2	0.33		0.24	
error.x3	0.22		0.26	
error.x4	0.23		0.19	
error.x5	0.39		0.31	
error.x6	0.70		0.44	
error.x7	0.60		0.45	
Sensitivity to economic risks	1.00		1.00	
Covariance:				
error.x1 with error.x2	0.56***		0.45***	
error.x3 with error.x5			0.17**	
error.x6 with error.x7	0.57***		0.58***	
N	505		488	
RMSEA	0.044		0.050	
CFI	0.971		0.952	
χ^2	23.929**		25.994***	
Cronbach's alpha for scale		0.905		0.937

[†] Standardized values. *** denotes significance at p<0.01. ** denotes significance at p<0.05. * denotes significance at p<0.1.

[‡] Cronbach's alpha if items are removed from the scale.
