# Supplementary material for: Investigating public support for biosecurity measures to mitigate pathogen transmission through the herpetological trade
Source: PLoS One. 2022 Jan 21;17(1):e0262719. doi: 10.1371/journal.pone.0262719 (PMC8782347; doi:10.1371/journal.pone.0262719)
Supplement: S21 Table — (PDF) [file pone.0262719.s023.pdf]

S21 Table. Respondents' trust in the government to mitigate pathogen transmission risks associated with the live herpetological trade (n=2,007).

	Median	Percent of respondents				
		SD†	D	N	A	SA
The government…						
has the knowledge to manage the amphibian and reptile disease transmission risk	0	7.3	14.8	30.1	31.1	16.7
has the money to manage the amphibian and reptile disease transmission risk	0	8.2	16.3	28.6	27.1	19.8
has sufficient skilled people to manage the amphibian and reptile disease transmission risk	0	9.9	21.8	30.8	25.0	12.5
has been effective in managing the amphibian and reptile disease transmission risk	0	11.6	22.3	42.5	17.4	6.3
can be trusted to properly manage the amphibian and reptile disease transmission risk	0	12.4	24.8	30.7	23.5	8.6

† SD: strongly disagree = 2; D: somewhat disagree = -1; N: neither agree nor disagree = 0; A: somewhat agree = 1; SA: strongly agree = 2
