# Supplementary material for: Investigating public support for biosecurity measures to mitigate pathogen transmission through the herpetological trade
Source: PLoS One. 2022 Jan 21;17(1):e0262719. doi: 10.1371/journal.pone.0262719 (PMC8782347; doi:10.1371/journal.pone.0262719)
Supplement: S22 Table — (PDF) [file pone.0262719.s024.pdf]

S22 Table. Confirmatory factor analysis for respondents' 'social trust' for different survey versions that presented the ecological impacts, economic impacts, human health and wellbeing impacts, or all impacts of pathogen transmission.

	Ecological impacts survey version		Economic impacts survey version		Human health and wellbeing impacts survey version		All impacts survey version	
	Coeff.[†]	Cronbach's alpha[‡]	Coeff.	Cronbach's alpha	Coeff.	Cronbach's alpha	Coeff.	Cronbach's alpha
Loadings:								
x1: government has the knowledge to manage the amphibian and reptile disease transmission risk	0.61***	0.752	0.79***	0.769	0.77***	0.776	0.80***	0.810
x2: government has the money to manage the amphibian and reptile disease transmission risk	0.84***	0.766	0.57***	0.823	0.60***	0.819	0.63***	0.854
x3: government has sufficient skilled people to manage the amphibian and reptile disease transmission risk	0.79***	0.817	0.79***	0.768	0.82***	0.765	0.88***	0.795
x4: government has been effective in managing the amphibian and reptile disease transmission risk	0.55***	0.806	0.64***	0.790	0.59***	0.800	0.64***	0.831
x5: government can be trusted to properly manage the amphibian and reptile disease transmission risk	0.68***	0.774	0.68***	0.780	0.68***	0.784	0.72***	0.814
Variances:								
error.x1	0.63		0.37		0.41		0.35	
error.x2	0.29		0.67		0.64		0.61	
error.x3	0.37		0.37		0.32		0.23	
error.x4	0.70		0.59		0.66		0.59	
error.x5	0.54		0.53		0.05		0.48	
Social trust	1.00		1.00		1.00		1.00	
Covariance:								
error.x4 with error.x5	0.32***		0.26***		0.28***		0.35***	
N	507		507		505		488	
RMSEA	0.069		0.046		0.046		0.049	

CFI	0.960		0.979		0.982		0.983	
χ^2	13.522***		8.273*		8.255*		8.684*	
Cronbach's alpha for scale		0.819		0.822		0.824		0.852

† Standardized values. *** denotes significance at $p<0.01$. ** denotes significance at $p<0.05$. * denotes significance at $p<0.1$.

‡ Cronbach's alpha if items are removed from the scale.
