# Supplementary material for: Investigating public support for biosecurity measures to mitigate pathogen transmission through the herpetological trade
Source: PLoS One. 2022 Jan 21;17(1):e0262719. doi: 10.1371/journal.pone.0262719 (PMC8782347; doi:10.1371/journal.pone.0262719)
Supplement: S23 Table — How similar is this person to you?” (n = 2,007). (PDF) [file pone.0262719.s025.pdf]

S23 Table. Distribution of responses to the Environmental Portrait Value Questionnaire: "Below are some statements about a random man/woman/person. How similar is this person to you?" (n=2,007).

	Median	Percent of respondents						
		1†	2	3	4	5	6	7
Biospheric values:								
It is important to him/her/them to prevent environmental pollution	6	1.5	1.7	2.8	13.7	21.8	25.5	33.1
It is important to him/her/them to protect the environment	6	0.9	1.0	2.4	9.2	19.4	26.2	40.9
It is important to him/her/them to respect nature	7	0.1	0.5	1.3	5.6	15.5	26.6	50.4
It is important to him/her/them to be in unity with nature	6	1.1	1.8	3.5	17.0	21.8	25.1	29.6
Altruistic values:								
It is important to him/her/them that every person has equal opportunities	6	1.2	0.5	1.5	9.5	16.9	24.3	46.0
It is important to him/her/them to take care of those who are worse off	5	1.7	2.0	2.7	17.4	26.4	24.5	25.3
It is important to him/her/them that every person is treated justly	7	0.2	0.6	1.2	6.2	14.0	25.7	52.1
It is important to him/her/them that there is no war or conflict	6	1.3	1.6	2.5	14.7	17.0	23.5	39.3
It is important to him/her/them to be helpful to others	6	0.5	0.3	1.0	6.4	18.4	29.8	43.6
Hedonic values:								
It is important to him/her/them to have fun	6	1.2	1.2	2.6	16.4	25.8	26.4	26.3
It is important to him/her/them to enjoy life's pleasures	6	0.5	0.7	2.2	10.4	22.9	27.7	35.5
It is important to him/her/them to do things he/she/they enjoy	6	0.1	0.8	1.0	6.5	21.1	29.8	40.6
Egoistic values:								
It is important to him/her/them to have control over others' actions	3	21.6	14.3	15.1	25.6	11.4	6.7	5.4
It is important to him/her/them to have authority over others	3	23.9	16.2	17.0	24.0	9.3	5.8	3.6
It is important to him/her/them to be influential	4	6.2	5.6	8.1	30.7	22.7	13.2	13.5
It is important to him/her/them to have money and possessions	5	7.1	7.6	10.0	24.4	25.4	14.4	11.1
It is important to him/her/them to work hard and be ambitious	6	1.2	1.8	2.8	12.7	21.4	25.2	34.8

† Not at all like me = 1; neutral = 4; very much like me = 7
