# Supplementary material for: Investigating public support for biosecurity measures to mitigate pathogen transmission through the herpetological trade
Source: PLoS One. 2022 Jan 21;17(1):e0262719. doi: 10.1371/journal.pone.0262719 (PMC8782347; doi:10.1371/journal.pone.0262719)
Supplement: S24 Table — (PDF) [file pone.0262719.s026.pdf]

S24 Table. Confirmatory factor analysis for respondents' 'biospheric values'.

	Ecological impacts survey version		Economic impacts survey version		Human health and wellbeing impacts survey version		All impacts survey version	
	Coeff.†	Cronbach's alpha‡	Coeff.	Cronbach's alpha	Coeff.	Cronbach's alpha	Coeff.	Cronbach's alpha
Loadings:								
x1: It is important to him/her/them to prevent environmental pollution	0.70***	0.810	0.71***	0.855	0.75***	0.853	0.76***	0.854
x2: It is important to him/her/them to protect the environment	0.81***	0.771	0.83***	0.816	0.87***	0.813	0.84***	0.829
x3: It is important to him/her/them to respect nature	0.84***	0.808	0.84***	0.852	0.81***	0.855	0.84***	0.855
x4: It is important to him/her/them to be in unity with nature	0.70***	0.848	0.81***	0.853	0.78***	0.862	0.79***	0.867
Variances:								
error.x1	0.51		0.49		0.44		0.42	
error.x2	0.30		0.30		0.25		0.30	
error.x3	0.52		0.30		0.34		0.29	
error.x4	0.35		0.34		0.39		0.38	
Biospheric values	1.00		1.00		1.00		1.00	
Covariance:								
error.x1 with error.x2	0.37***		0.41***		0.29***		0.30***	
N	507		507		505		488	
RMSEA	<0.001		<0.001		<0.001		<0.001	
CFI	1.000		1.000		1.000		1.000	
χ^2	0.326		3.510*		0.250		0.350	
Cronbach's alpha for scale		0.850		0.878		0.880		0.884

† Standardized values. *** denotes significance at p<0.01. ** denotes significance at p<0.05. * denotes significance at p<0.1.

‡ Cronbach's alpha if items are removed from the scale.
