# Supplementary material for: Investigating public support for biosecurity measures to mitigate pathogen transmission through the herpetological trade
Source: PLoS One. 2022 Jan 21;17(1):e0262719. doi: 10.1371/journal.pone.0262719 (PMC8782347; doi:10.1371/journal.pone.0262719)
Supplement: S25 Table — (PDF) [file pone.0262719.s027.pdf]

S25 Table. Confirmatory factor analysis for respondents' 'altruistic values'.

	Ecological impacts survey version		Economic impacts survey version		Human health and wellbeing impacts survey version		All impacts survey version	
	Coeff.[†]	Cronbach's alpha[‡]	Coeff.	Cronbach's alpha	Coeff.	Cronbach's alpha	Coeff.	Cronbach's alpha
Loadings:								
x1: It is important to him/her/them that every person has equal opportunities	0.78***	0.722	0.81***	0.768	0.70***	0.749	0.71***	0.768
x2: It is important to him/her/them to take care of those who are worse off	0.53***	0.764	0.61***	0.799	0.57***	0.768	0.72***	0.789
x3: It is important to him/her/them that every person is treated justly	0.78***	0.727	0.76***	0.779	0.77***	0.757	0.84***	0.767
x4: It is important to him/her/them that there is no war or conflict	0.52***	0.786	0.58***	0.821	0.58***	0.800	0.56***	0.805
x5: It is important to him/her/them to be helpful to others	0.65***	0.732	0.70***	0.777	0.68***	0.759	0.74***	0.766
Variances:								
error.x1	0.40		0.34		0.51		0.49	
error.x2	0.72		0.63		0.68		0.49	
error.x3	0.39		0.42		0.41		0.29	
error.x4	0.73		0.66		0.66		0.68	
error.x5	0.58		0.51		0.54		0.45	
Altruistic values	1.00		1.00		1.00		1.00	
Covariance:								
error.x1 with error.x2					0.24***			
error.x2 with error.x3							-0.47***	
error.x2 with error.x5	0.24***		0.25***		0.26***			
N	507		507		505		488	
RMSEA	<0.001		<0.001		<0.001		0.017	
CFI	1.000		1.000		1.000		0.995	
χ^2	2.157		0.179		1.424		4.586	
Cronbach's alpha for scale		0.786		0.824		0.804		0.815

[†] Standardized values. *** denotes significance at p<0.01. ** denotes significance at p<0.05. * denotes significance at p<0.1.

‡ Cronbach's alpha if items are removed from the scale.
