# Supplementary material for: Investigating public support for biosecurity measures to mitigate pathogen transmission through the herpetological trade
Source: PLoS One. 2022 Jan 21;17(1):e0262719. doi: 10.1371/journal.pone.0262719 (PMC8782347; doi:10.1371/journal.pone.0262719)
Supplement: S26 Table — (PDF) [file pone.0262719.s028.pdf]

S26 Table. Confirmatory factor analysis for respondents' 'hedonic values'.

	Ecological impacts survey version		Economic impacts survey version		Human health and wellbeing impacts survey version		All impacts survey version	
	Coeff.†	Cronbach's alpha‡	Coeff.	Cronbach's alpha	Coeff.	Cronbach's alpha	Coeff.	Cronbach's alpha
Loadings:								
x1: It is important to him/her/them to have fun	0.77***	0.741	0.72***	0.801	0.67***	0.767	0.76***	0.776
x2: It is important to him/her/them to enjoy life's pleasures	0.76***	0.737	0.85***	0.720	0.84***	0.659	0.82***	0.736
x3: It is important to him/her/them to do things he/she/they enjoy	0.78***	0.738	0.79***	0.759	0.75***	0.715	0.78***	0.768
Variances:								
error.x1	0.41		0.48		0.56		0.42	
error.x2	0.42		0.28		0.30		0.33	
error.x3	0.40		0.37		0.44		0.39	
Hedonic values	1.00		1.00		1.00		1.00	
N	507		507		505		488	
RMSEA	0.000		0.000		0.000		0.000	
CFI	1.000		1.000		1.000		1.000	
Cronbach's alpha for scale		0.809		0.826		0.789		0.826

† Standardized values. *** denotes significance at $p<0.01$. ** denotes significance at $p<0.05$. * denotes significance at $p<0.1$.

‡ Cronbach's alpha if items are removed from the scale.
