# Supplementary material for: Investigating public support for biosecurity measures to mitigate pathogen transmission through the herpetological trade
Source: PLoS One. 2022 Jan 21;17(1):e0262719. doi: 10.1371/journal.pone.0262719 (PMC8782347; doi:10.1371/journal.pone.0262719)
Supplement: S27 Table — (PDF) [file pone.0262719.s029.pdf]

S27 Table. Confirmatory factor analysis for respondents' 'egoistic values'.

	Ecological impacts survey version		Economic impacts survey version		Human health and wellbeing impacts survey version		All impacts survey version	
	Coeff.†	Cronbach's alpha‡	Coeff.	Cronbach's alpha	Coeff.	Cronbach's alpha	Coeff.	Cronbach's alpha
Loadings:								
x1: It is important to him/her/them to have control over others' actions	0.59***	0.673	0.64***	0.630	0.73***	0.630	0.04***	0.641
x2: It is important to him/her/them to have authority over others	0.59***	0.636	0.87***	0.635	0.84***	0.644	0.05***	0.622
x3: It is important to him/her/them to be influential	0.65***	0.742	0.69***	0.694	0.63***	0.725	0.05***	0.743
x4: It is important to him/her/them to have money and possessions	0.58***	0.735	0.49***	0.696	0.50***	0.734	0.04***	0.751
Variances:								
error.x1	0.65		0.60		0.46		0.06	
error.x2	0.65		0.25		0.30		0.08	
error.x3	0.58		0.52		0.60		0.06	
error.x4	0.66		0.76		0.75		0.04	
Egoistic values	1.00		1.00		1.00			
Covariance:								
error.x1 with error.x2	0.49***							
error.x2 with error.x3			-0.83**		-0.40**		-0.45	
error.x2 with error.x4	0.17***							
N	507		507		505		488	
RMSEA	<0.001		0.042		<0.001		<0.001	
CFI	1.000		0.996		1.000		1.000	
χ^2			1.911		0.294		0.204	
Cronbach's alpha for scale		0.757		0.726		0.745		0.752

† Standardized values. *** denotes significance at p<0.01. ** denotes significance at p<0.05. * denotes significance at p<0.1.
‡ Cronbach's alpha if items are removed from the scale.
