# Supplementary material for: Investigating public support for biosecurity measures to mitigate pathogen transmission through the herpetological trade
Source: PLoS One. 2022 Jan 21;17(1):e0262719. doi: 10.1371/journal.pone.0262719 (PMC8782347; doi:10.1371/journal.pone.0262719)
Supplement: S28 Table — (PDF) [file pone.0262719.s030.pdf]

S28 Table. Structural equation model of respondents' support for improved biosecurity measures when presented with the ecological risks associated with pathogen transmission through the live herpetological trade (model 1, n=507).

	Coef.	Std. Err.	p
Structural Regression			
Support for biosecurity			
Importance of protecting the health of humans	0.108	0.047	0.020
Sensitivity to ecological risks	0.330	0.043	<0.001
Social trust	0.081	0.049	0.095
Altruistic values	0.258	0.065	<0.001
Egoistic values	-0.182	0.067	0.007
Hedonic values	0.135	0.070	0.055
Sensitivity to ecological risks			
Attitudes towards herpetofauna	0.139	0.031	<0.001
Sensitivity to herpetological trade risks	0.180	0.040	<0.001
Perceived susceptibility to herpetological disease transmission	0.400	0.044	<0.001
Perceived susceptibility to ecological risks (loss of biodiversity owing to herpetological diseases)	0.418	0.041	<0.001
Biospheric values	0.166	0.046	<0.001
Hedonic values	-0.082	0.036	0.022
Sensitivity to herpetological trade risks			
Years of education	-0.060	0.040	0.137
Hispanic and/or Latino	0.112	0.040	0.005
Biospheric values	0.364	0.048	<0.001
Perceived susceptibility to herpetological pathogen transmission			
Perceived percentage of captive amphibians and reptiles in the live animal trade that are healthy	-0.126	0.041	<0.001
Prior knowledge of salmonella	0.098	0.041	0.016
Like freshwater fish	0.054	0.062	0.379
Like saltwater fish	0.164	0.062	0.008
Social trust			
Years of education	-0.122	0.047	0.009
Household contains members under 18 years	0.251	0.046	<0.001
Measurement Models			
Support for biosecurity			
x1: A law that requires the quarantine and veterinary observation of all amphibians and reptiles imported into the United States	0.753	0.024	<0.001

x2: Mandatory tests of all shipments of amphibians and reptiles for selected diseases of concern	0.857	0.021	<0.001
x3: Mandatory 'Best Practices Program' requiring live amphibian and reptile importers and exporters to improve care and reduce stress of transported animals and decontaminate all shipping materials	0.746	0.025	<0.001
Sensitivity to ecological risks			
x1: Chytrid transmitted to other captive amphibians	0.838	0.013	<0.001
x2: Chytrid transmitted to native amphibians	0.864	0.012	<0.001
x3: Ranavirus transmitted to other captive amphibians and reptiles	0.828	0.014	<0.001
x4: Ranavirus transmitted to native amphibians and reptiles	0.917	0.010	<0.001
x5: Ranavirus transmitted to native fish	0.835	0.014	<0.001
x6: Loss of biodiversity	0.776	0.018	<0.001
Covariance: error.x1 with error.x2	0.408	0.039	<0.001
Covariance: error.x1 with error.x3	0.394	0.031	<0.001
Covariance: error.x3 with error.x4	0.442	0.043	<0.001
Covariance: error.x4 with error.x6	-0.324	0.058	<0.001
Attitudes towards herpetofauna			
x1: Snakes	0.651	0.028	<0.001
x2: Lizards	0.890	0.013	<0.001
x3: Turtles/tortoises	0.672	0.027	<0.001
x4: Frogs	0.794	0.019	<0.001
x5: Toads	0.746	0.023	<0.001
x6: Salamanders/newts	0.838	0.016	<0.001
Covariance: error.x4 with error.x5	0.489	0.038	<0.001
Sensitivity to herpetological trade risks			
x1: Other captive amphibians	0.746	0.023	<0.001
x2: Native wildlife	0.832	0.017	<0.001
x3: Pets	0.845	0.016	<0.001
x4: Livestock	0.875	0.015	<0.001
x5: Humans	0.609	0.031	<0.001
Covariance: error.x1 with error.x2	0.359	0.050	<0.001
Covariance: error.x4 with error.x5	0.261	0.055	<0.001
Perceived susceptibility to herpetological pathogen transmission			
x1: Chytrid transmitted to other captive amphibians	0.684	0.026	<0.001
x2: Chytrid transmitted to native amphibians	0.788	0.021	<0.001
x3: Ranavirus transmitted to other captive amphibians and reptiles	0.693	0.026	<0.001

x4: Ranavirus transmitted to native amphibians and reptiles	0.822	0.020	<0.001
x5: Ranavirus transmitted to native fish	0.761	0.023	<0.001
x6: Salmonella transmitted to other captive amphibians and reptiles	0.662	0.028	<0.001
x7: Salmonella transmitted to native amphibians and reptiles	0.790	0.021	<0.001
x8: Salmonella transmitted to pets	0.627	0.031	<0.001
x9: Salmonella transmitted to livestock	0.631	0.030	<0.001
x10: Salmonella transmitted to humans	0.628	0.030	<0.001
Covariance: error.x1 with error.x2	0.395	0.038	<0.001
Covariance: error.x1 with error.x3	0.393	0.031	<0.001
Covariance: error.x1 with error.x6	0.295	0.031	<0.001
Covariance: error.x3 with error.x4	0.515	0.035	<0.001
Covariance: error.x3 with error.x5	0.295	0.043	<0.001
Covariance: error.x3 with error.x6	0.290	0.029	<0.001
Covariance: error.x4 with error.x5	0.520	0.042	<0.001
Covariance: error.x6 with error.x7	0.555	0.033	<0.001
Covariance: error.x7 with error.x9	0.167	0.032	<0.001
Covariance: error.x8 with error.x9	0.563	0.033	<0.001
Covariance: error.x8 with error.x10	0.464	0.038	<0.001
Covariance: error.x9 with error.x10	0.385	0.040	<0.001
Social trust			
x1: Government has the knowledge to manage the amphibian and reptile disease transmission risk	0.770	0.026	<0.001
x2: Government has the money to manage the amphibian and reptile disease transmission risk	0.600	0.034	<0.001
x3: Government has sufficient skilled people to manage the amphibian and reptile disease transmission risk	0.830	0.023	<0.001
x4: Government has been effective in managing the amphibian and reptile disease transmission risk	0.536	0.037	<0.001
x5: Government can be trusted to properly manage the amphibian and reptile disease transmission risk	0.670	0.030	<0.001
Covariance: error.x4 with error.x5	0.341	0.044	<0.001
Altruistic values			
x1: It is important to him/her/them that every person has equal opportunities	0.736	0.026	<0.001
x2: It is important to him/her/them to take care of those who are worse off	0.532	0.037	<0.001
x3: It is important to him/her/them that every person is treated justly	0.770	0.024	<0.001
x4: It is important to him/her/them that there is no war or conflict	0.570	0.034	<0.001

x5: It is important to him/her/them to be helpful to others	0.674	0.029	<0.001
Covariance: error.x2 with error.x5	0.247	0.047	<0.001
Biospheric values			
x1: It is important to him/her/them to prevent environmental pollution	0.751	0.024	<0.001
x2: It is important to him/her/them to protect the environment	0.798	0.021	<0.001
x3: It is important to him/her/them to respect nature	0.810	0.020	<0.001
x4: It is important to him/her/them to be in unity with nature	0.696	0.027	<0.001
Covariance: error.x1 with error.x2	0.295	0.053	<0.001
Egoistic values			
x1: It is important to him/her/them to have control over others' actions	0.477	0.044	<0.001
x2: It is important to him/her/them to have authority over others	0.462	0.045	<0.001
x3: It is important to him/her/them to be influential	0.776	0.042	<0.001
x4: It is important to him/her/them to have money and possessions	0.536	0.045	<0.001
Covariance: error.x1 with error.x2	0.551	0.032	<0.001
Covariance: error.x2 with error.x4	0.218	0.038	<0.001
Hedonic values			
x1: It is important to him/her/them to have fun	0.786	0.025	<0.001
x2: It is important to him/her/them to enjoy life's pleasures	0.761	0.026	<0.001
x3: It is important to him/her/them to do things he/she/they enjoy	0.762	0.026	<0.001
Covariance: error.Concern about herpetological trade with error.Herpetological disease risk perceptions	0.512	0.044	<0.001
Covariance: like freshwater fish with like saltwater fish	0.750	0.019	<0.001
Root mean squared error of approximation (RMSEA)	0.048		
Comparative fit index	0.904		
Akaike's information criterion (AIC)	74,357.091		
Bayesian information criterion (BIC)	75,663.701		
