# Supplementary material for: Investigating public support for biosecurity measures to mitigate pathogen transmission through the herpetological trade
Source: PLoS One. 2022 Jan 21;17(1):e0262719. doi: 10.1371/journal.pone.0262719 (PMC8782347; doi:10.1371/journal.pone.0262719)
Supplement: S29 Table — (PDF) [file pone.0262719.s031.pdf]

S29 Table. Structural equation model of respondents' support for improved biosecurity measures when presented with the economic risks associated with pathogen transmission through the live herpetological trade (model 2, n=507).

	Coef.	Std. Err.	p
Structural Regression			
Support for biosecurity			
Sensitivity to economic risks	0.234	0.069	0.001
Perceived susceptibility to herpetological disease transmission	0.138	0.062	0.026
Biospheric values	0.258	0.059	<0.001
Egoistic values	-0.075	0.045	0.097
Sensitivity to economic risks			
Perceived susceptibility to economic risks	0.915	0.023	<0.001
Sensitivity to general health risks	0.126	0.030	<0.001
Egoistic values	0.064	0.026	0.014
Perceived susceptibility to herpetological pathogen transmission			
Perceived percentage of captive amphibians and reptiles in the live animal trade that are healthy	-0.291	0.042	<0.001
Prior knowledge of chytrid	0.076	0.045	0.091
Prior knowledge of ranavirus	-0.080	0.045	0.075
Prior knowledge of salmonella	0.079	0.044	0.071
Like freshwater fish	0.168	0.084	0.046
Like saltwater fish	0.132	0.084	0.118
Sensitivity to general health risks			
Female	0.161	0.040	<0.001
Age (years)	0.017	0.041	0.686
Black	-0.019	0.040	0.638
Political views	-0.032	0.041	0.439
Biospheric values	0.605	0.036	<0.001
Egoistic values	-0.051	0.042	0.218
Measurement Models			
Support for biosecurity			
x1: A law that requires the quarantine and veterinary observation of all amphibians and reptiles imported into the United States	0.839	0.020	<0.001
x2: Mandatory tests of all shipments of amphibians and reptiles for selected diseases of concern	0.860	0.019	<0.001

x3: Mandatory 'Best Practices Program' requiring live amphibian and reptile importers and exporters to improve care and reduce stress of transported animals and decontaminate all shipping materials	0.713	0.026	<0.001
Sensitivity to economic risks			
x1: Agriculture	0.781	0.023	<0.001
x2: Aquaculture	0.843	0.020	<0.001
x3: Amphibian and reptile trade	0.736	0.025	<0.001
x4: Frog leg market	0.652	0.028	<0.001
Covariance: error.x3 with error.x4	0.538	0.034	<0.001
Perceived susceptibility to economic risks			
x1: Agriculture	0.759	0.024	<0.001
x2: Aquaculture	0.844	0.020	<0.001
x3: Amphibian and reptile trade	0.742	0.025	<0.001
x4: Frog leg market	0.707	0.027	<0.001
Covariance: error.x1 with error.x2	0.194	0.050	<0.001
Covariance: error.x3 with error.x4	0.601	0.033	<0.001
Sensitivity to general health risks			
x1: Animals in the live animal trade	0.865	0.016	<0.001
x2: Native wildlife	0.755	0.023	<0.001
x3: The natural environment	0.760	0.026	<0.001
x4: Pets	0.720	0.024	<0.001
x5: Livestock	0.817	0.018	<0.001
Covariance: error.x1 with error.x3	-0.285	0.061	<0.001
Covariance: error.x2 with error.x3	0.537	0.041	<0.001
Perceived susceptibility to herpetological pathogen transmission			
x1: Chytrid transmitted to other captive amphibians	0.704	0.025	<0.001
x2: Chytrid transmitted to native amphibians	0.764	0.022	<0.001
x3: Ranavirus transmitted to other captive amphibians and reptiles	0.784	0.021	<0.001
x4: Ranavirus transmitted to native amphibians and reptiles	0.875	0.017	<0.001
x5: Ranavirus transmitted to native fish	0.818	0.021	<0.001
x6: Salmonella transmitted to other captive amphibians and reptiles	0.658	0.029	<0.001
x7: Salmonella transmitted to native amphibians and reptiles	0.764	0.023	<0.001
x8: Salmonella transmitted to pets	0.678	0.029	<0.001
x9: Salmonella transmitted to livestock	0.662	0.030	<0.001
x10: Salmonella transmitted to humans	0.674	0.028	<0.001
Covariance: error.x1 with error.x2	0.497	0.032	<0.001

Covariance: error.x1 with error.x3	0.441	0.030	<0.001
Covariance: error.x1 with error.x6	0.264	0.028	<0.001
Covariance: error.x3 with error.x4	0.380	0.049	<0.001
Covariance: error.x3 with error.x5	0.253	0.050	<0.001
Covariance: error.x3 with error.x6	0.287	0.029	<0.001
Covariance: error.x3 with error.x9	-0.101	0.030	0.001
Covariance: error.x4 with error.x5	0.461	0.055	<0.001
Covariance: error.x6 with error.x7	0.654	0.027	<0.001
Covariance: error.x8 with error.x9	0.527	0.037	<0.001
Covariance: error.x8 with error.x10	0.383	0.043	<0.001
Covariance: error.x9 with error.x10	0.393	0.042	<0.001
Biospheric values			
x1: It is important to him/her/them to prevent environmental pollution	0.731	0.026	<0.001
x2: It is important to him/her/them to protect the environment	0.821	0.019	<0.001
x3: It is important to him/her/them to respect nature	0.830	0.019	<0.001
x4: It is important to him/her/them to be in unity with nature	0.794	0.021	<0.001
Covariance: error.x1 with error.x2	0.338	0.051	<0.001
Egoistic values			
x1: It is important to him/her/them to have control over others' actions	0.628	0.036	<0.001
x2: It is important to him/her/them to have authority over others	0.863	0.041	<0.001
x3: It is important to him/her/them to be influential	0.730	0.048	<0.001
x4: It is important to him/her/them to have money and possessions	0.488	0.040	<0.001
Covariance: error.x2 with error.x3	-0.954	0.300	0.001
Covariance: sensitivity to economic risks (agriculture) with perceived susceptibility to economic risks (agriculture)	0.491	0.040	<0.001
Covariance: sensitivity to economic risks (aquaculture) with perceived susceptibility to economic risks (aquaculture)	0.356	0.057	<0.001
Covariance: sensitivity to economic risks (frog leg market) with perceived susceptibility to economic risks (frog leg market)	0.394	0.037	<0.001
Covariance: sensitivity to economic risks (frog leg market) with perceived susceptibility to economic risks (amphibian and reptile trade)	0.235	0.041	<0.001
Covariance: like freshwater fish with like saltwater fish	0.858	0.012	<0.001
Root mean squared error of approximation (RMSEA)	0.051		
Comparative fit index	0.921		
Akaike's information criterion (AIC)	51,906.519		
Bayesian information criterion (BIC)	52,942.504		
