# Supplementary material for: Investigating public support for biosecurity measures to mitigate pathogen transmission through the herpetological trade
Source: PLoS One. 2022 Jan 21;17(1):e0262719. doi: 10.1371/journal.pone.0262719 (PMC8782347; doi:10.1371/journal.pone.0262719)
Supplement: S30 Table — (PDF) [file pone.0262719.s032.pdf]

S30 Table. Structural equation model of respondents' support for improved biosecurity measures when presented with the human health and wellbeing risks associated with pathogen transmission through the live herpetological trade (model 3, n=505).

	Coef.	Std. Err.	p
Structural Regression			
Support for biosecurity			
Sensitivity to human health and wellbeing risks	0.226	0.069	<0.001
Importance of protecting the health of humans	0.076	0.045	0.090
Agreement that the occurrence of wildlife disease has been made worse by humans and their activities	0.098	0.044	0.025
Perceived susceptibility to herpetological disease transmission	0.094	0.069	0.172
Altruistic values	0.315	0.054	<0.001
Egoistic values	-0.148	0.046	0.001
Sensitivity to human health and wellbeing risks			
Perceived susceptibility to human health and wellbeing risks (increase in insect pests owing to herpetological disease transmission)	0.068	0.046	0.136
Perceived susceptibility to human health and wellbeing risks (increase in insect-borne diseases owing to herpetological disease transmission)	0.268	0.048	<0.001
Perceived susceptibility to herpetological disease transmission	0.352	0.044	<0.001
Sensitivity to herpetological trade risks	0.430	0.040	<0.001
Like freshwater fish	0.088	0.041	0.032
Like saltwater fish	-0.046	0.041	0.256
Egoistic values	0.066	0.032	0.038
Sensitivity to herpetological trade risks			
Knowledge about the animal trade	0.086	0.046	0.061
Knowledge of number of live amphibians and live reptiles imported into the United States	0.134	0.043	0.002
Respondent fishes	0.060	0.043	0.164
Female	0.086	0.042	0.042
Years of education	-0.140	0.043	0.001
Hispanic and/or Latino	0.064	0.042	0.126
Biospheric values	0.425	0.042	<0.001
Egoistic values	0.097	0.046	0.037
Measurement Models			
Support for biosecurity			

x1: A law that requires the quarantine and veterinary observation of all amphibians and reptiles imported into the United States	0.790	0.023	<0.001
x2: Mandatory tests of all shipments of amphibians and reptiles for selected diseases of concern	0.852	0.021	<0.001
x3: Mandatory ‘Best Practices Program’ requiring live amphibian and reptile importers and exporters to improve care and reduce stress of transported animals and decontaminate all shipping materials	0.727	0.026	<0.001
Sensitivity to human health and wellbeing risks			
x1: Salmonella transmitted to other captive amphibians	0.729	0.023	<0.001
x2: Salmonella transmitted to native amphibians	0.803	0.019	<0.001
x3: Salmonella transmitted to pets	0.802	0.020	<0.001
x4: Salmonella transmitted to livestock	0.768	0.022	<0.001
x5: Salmonella transmitted to humans	0.694	0.025	<0.001
x6: Increase in insect pests	0.587	0.032	<0.001
x7: Increase in insect-borne diseases	0.670	0.028	<0.001
Covariance: error.x1 with error.x2	0.566	0.034	<0.001
Covariance: error.x3 with error.x4	0.303	0.049	<0.001
Covariance: error.x6 with error.x7	0.512	0.037	<0.001
Perceived susceptibility to herpetological pathogen transmission			
x1: Chytrid transmitted to other captive amphibians	0.688	0.026	<0.001
x2: Chytrid transmitted to native amphibians	0.769	0.022	<0.001
x3: Ranavirus transmitted to other captive amphibians and reptiles	0.717	0.025	<0.001
x4: Ranavirus transmitted to native amphibians and reptiles	0.825	0.019	<0.001
x5: Ranavirus transmitted to native fish	0.804	0.020	<0.001
x6: Salmonella transmitted to other captive amphibians and reptiles	0.827	0.021	<0.001
x7: Salmonella transmitted to native amphibians and reptiles	0.757	0.021	<0.001
x8: Salmonella transmitted to pets	0.702	0.025	<0.001
x9: Salmonella transmitted to livestock	0.664	0.026	<0.001
x10: Salmonella transmitted to humans	0.627	0.030	<0.001
Covariance: error.x1 with error.x2	0.299	0.047	<0.001
Covariance: error.x1 with error.x3	0.275	0.039	<0.001
Covariance: error.x2 with error.x3	-0.090	0.046	0.053
Covariance: error.x2 with error.x6	-0.437	0.063	<0.001
Covariance: error.x3 with error.x4	0.389	0.044	<0.001
Covariance: error.x3 with error.x5	0.271	0.048	<0.001
Covariance: error.x4 with error.x5	0.460	0.047	<0.001

Covariance: error.x4 with error.x6	-0.396	0.065	<0.001
Covariance: error.x4 with error.x8	-0.135	0.038	<0.001
Covariance: error.x4 with error.x10	-0.101	0.038	0.008
Covariance: error.x5 with error.x6	-0.371	0.065	<0.001
Covariance: error.x5 with error.x8	-0.088	0.038	0.022
Covariance: error.x6 with error.x7	0.352	0.047	<0.001
Covariance: error.x6 with error.x10	-0.148	0.046	0.001
Covariance: error.x7 with error.x8	0.323	0.036	<0.001
Covariance: error.x7 with error.x9	0.186	0.035	<0.001
Covariance: error.x8 with error.x9	0.553	0.031	<0.001
Covariance: error.x8 with error.x10	0.299	0.039	<0.001
Covariance: error.x9 with error.x10	0.293	0.039	<0.001
Sensitivity to herpetological trade risks			
x1: Other captive amphibians	0.758	0.023	<0.001
x2: Native wildlife	0.823	0.018	<0.001
x3: Pets	0.880	0.015	<0.001
x4: Livestock	0.817	0.018	<0.001
x5: Humans	0.644	0.029	<0.001
Covariance: error.x1 with error.x2	0.353	0.047	<0.001
Covariance: error.x4 with error.x5	0.208	0.046	<0.001
Altruistic values			
x1: It is important to him/her/them that every person has equal opportunities	0.686	0.029	<0.001
x2: It is important to him/her/them to take care of those who are worse off	0.597	0.035	<0.001
x3: It is important to him/her/them that every person is treated justly	0.757	0.025	<0.001
x4: It is important to him/her/them that there is no war or conflict	0.603	0.033	<0.001
x5: It is important to him/her/them to be helpful to others	0.672	0.030	<0.001
Covariance: error.x1 with error.x2	0.228	0.047	<0.001
Covariance: error.x2 with error.x5	0.224	0.046	<0.001
Biospheric values			
x1: It is important to him/her/them to prevent environmental pollution	0.749	0.024	<0.001
x2: It is important to him/her/them to protect the environment	0.849	0.017	<0.001
x3: It is important to him/her/them to respect nature	0.827	0.018	<0.001
x4: It is important to him/her/them to be in unity with nature	0.770	0.022	<0.001
Covariance: error.x1 with error.x2	0.302	0.055	<0.001
Egoistic values			
x1: It is important to him/her/them to have control over others' actions	0.704	0.035	<0.001

x2: It is important to him/her/them to have authority over others	0.848	0.037	<0.001
x3: It is important to him/her/them to be influential	0.690	0.045	<0.001
x4: It is important to him/her/them to have money and possessions	0.480	0.039	<0.001
Covariance: error.x2 with error.x3	-0.601	0.195	0.002
Covariance: perceived risk that herpetological diseases can result in an increase in insect pests with perceived risk that herpetological diseases can result in an increase in insect-borne diseases	0.732	0.021	<0.001
Covariance: like freshwater fish with like saltwater fish	0.688	0.023	<0.001
Covariance: concern about herpetological trade (other captive amphibians) with sensitivity to human health and wellbeing risks (salmonella transmitted to other captive amphibians)	0.174	0.033	<0.001
Covariance: concern about herpetological trade (pets) with sensitivity to human health and wellbeing risks (salmonella transmitted to native amphibians and reptiles)	-0.279	0.044	<0.001
Covariance: concern about herpetological trade (livestock) with sensitivity to human health and wellbeing risks (salmonella transmitted to livestock)	0.236	0.042	<0.001
Covariance: concern about herpetological trade (humans) with sensitivity to human health and wellbeing risks (salmonella transmitted to humans)	0.343	0.039	<0.001
Covariance: herpetological disease risk perceptions (ranavirus transmitted to other captive amphibians and reptiles) with sensitivity to human health and wellbeing risks (salmonella transmitted to other captive amphibians)	0.175	0.030	<0.001
Covariance: herpetological pathogen risk perceptions (salmonella transmitted to other captive amphibians and reptiles) with sensitivity to human health and wellbeing risks (salmonella transmitted to other captive amphibians)	0.204	0.037	<0.001
Covariance: herpetological pathogen risk perceptions (salmonella transmitted to livestock) with sensitivity to human health and wellbeing risks (salmonella transmitted to livestock)	0.300	0.033	<0.001
Covariance: herpetological pathogen risk perceptions (salmonella transmitted to humans) with sensitivity to human health and wellbeing risks (salmonella transmitted to humans)	0.334	0.039	<0.001
Root mean squared error of approximation (RMSEA)	0.050		
Comparative fit index	0.907		
Akaike's information criterion (AIC)	60,987.603		
Bayesian information criterion (BIC)	62,318.338		
