# Supplementary material for: Investigating public support for biosecurity measures to mitigate pathogen transmission through the herpetological trade
Source: PLoS One. 2022 Jan 21;17(1):e0262719. doi: 10.1371/journal.pone.0262719 (PMC8782347; doi:10.1371/journal.pone.0262719)
Supplement: S31 Table — (PDF) [file pone.0262719.s033.pdf]

S31 Table. Structural equation model of respondents' support for improved biosecurity measures when presented with the ecological, economic, and human health and wellbeing risks associated with pathogen transmission through the live herpetological trade (model 4, n=488).

	Coef.	Std. Err.	p
Structural Regression			
Support for biosecurity			
Importance of protecting the health of humans	0.116	0.046	0.013
Sensitivity to human health and wellbeing risks	0.366	0.052	<0.001
Sensitivity to general health risks	0.164	0.052	0.002
Perceived susceptibility to herpetological disease transmission	0.120	0.063	0.055
Sensitivity to human health and wellbeing risks			
Perceived susceptibility to human health and wellbeing risks (increase in insect pests owing to herpetological disease transmission)	0.257	0.052	<0.001
Perceived susceptibility to human health and wellbeing risks (increase in insect-borne diseases owing to herpetological disease transmission)	0.230	0.056	<0.001
Sensitivity to ecological risks	0.243	0.057	<0.001
Sensitivity to economic risks	0.387	0.073	<0.001
Perceived susceptibility to herpetological disease transmission	0.096	0.042	0.024
Sensitivity to herpetological trade risks	0.243	0.042	<0.001
Attitudes towards herpetofauna	-0.104	0.031	0.001
Sensitivity to ecological risks			
Like freshwater fish	0.117	0.047	0.013
Like saltwater fish	-0.095	0.047	0.043
Perceived susceptibility to ecological risks (loss of biodiversity from herpetological disease transmission)	0.682	0.030	<0.001
Biospheric values	0.201	0.039	<0.001
Sensitivity to economic risks			
Perceived susceptibility to economic risks	0.970	0.015	<0.001
Hedonic values	0.093	0.028	0.001
Perceived susceptibility to economic risks			
Egoistic values	0.174	0.040	<0.001
Sensitivity to general health risks			
Agreement that most environmental problems are caused by humans interfering with nature	0.096	0.055	0.084
Agreement that the occurrence of wildlife disease has been made worse by humans and their activities	0.130	0.052	0.012

Altruistic values	0.505	0.045	<0.001
Perceived susceptibility to herpetological pathogen transmission			
Perceived percentage of captive amphibians and reptiles in the live animal trade that are healthy	-0.061	0.035	0.080
Prior knowledge of chytrid	-0.040	0.039	0.302
Prior knowledge of ranavirus	0.051	0.038	0.177
Prior knowledge of salmonella	0.065	0.034	0.055
Hedonic values	0.141	0.041	0.001
Sensitivity to herpetological trade risks			
Knowledge of herpetological imports	0.107	0.051	0.036
Female	0.151	0.043	0.001
Black/African American	0.081	0.042	0.054
Biospheric values	0.481	0.040	<0.001
Measurement Models			
Support for biosecurity			
x1: A law that requires the quarantine and veterinary observation of all amphibians and reptiles imported into the United States	0.799	0.021	<0.001
x2: Mandatory tests of all shipments of amphibians and reptiles for selected diseases of concern	0.872	0.019	<0.001
x3: Mandatory 'Best Practices Program' requiring live amphibian and reptile importers and exporters to improve care and reduce stress of transported animals and decontaminate all shipping materials	0.704	0.025	<0.001
Sensitivity to human health and wellbeing risks			
x1: Salmonella transmitted to other captive amphibians	0.749	0.023	<0.001
x2: Salmonella transmitted to native amphibians	0.713	0.026	<0.001
x3: Salmonella transmitted to pets	0.670	0.028	<0.001
x4: Salmonella transmitted to livestock	0.706	0.027	<0.001
x5: Salmonella transmitted to humans	0.621	0.029	<0.001
x6: Increase in insect pests	0.766	0.022	<0.001
x7: Increase in insect-borne diseases	0.788	0.022	<0.001
Covariance: error.x1 with error.x2	0.580	0.038	<0.001
Covariance: error.x3 with error.x5	0.421	0.046	<0.001
Covariance: error.x6 with error.x7	0.306	0.062	<0.001
Sensitivity to ecological risks			
x1: Chytrid transmitted to other captive amphibians	0.839	0.014	<0.001
x2: Chytrid transmitted to native amphibians	0.886	0.011	<0.001

x3: Ranavirus transmitted to other captive amphibians and reptiles	0.877	0.012	<0.001
x4: Ranavirus transmitted to native amphibians and reptiles	0.948	0.006	<0.001
x5: Ranavirus transmitted to native fish	0.911	0.009	<0.001
x6: Loss of biodiversity	0.813	0.017	<0.001
Covariance: error.x1 with error.x2	0.372	0.035	<0.001
Covariance: error.x1 with error.x3	0.530	0.032	<0.001
Sensitivity to economic risks			
x1: Agriculture	0.820	0.020	<0.001
x2: Aquaculture	0.835	0.019	<0.001
x3: Amphibian and reptile trade	0.706	0.026	<0.001
x4: Frog leg market	0.578	0.033	<0.001
Covariance: error.x3 with error.x4	0.559	0.032	<0.001
Perceived susceptibility to economic risks			
x1: Agriculture	0.808	0.019	<0.001
x2: Aquaculture	0.850	0.017	<0.001
x3: Amphibian and reptile trade	0.815	0.019	<0.001
x4: Frog leg market	0.735	0.024	<0.001
Covariance: error.x1 with error.x2	0.182	0.050	<0.001
Covariance: error.x3 with error.x4	0.485	0.039	<0.001
Sensitivity to general health risks			
x1: Animals in the live animal trade	0.881	0.014	<0.001
x2: Native wildlife	0.815	0.019	<0.001
x3: The natural environment	0.760	0.023	<0.001
x4: Pets	0.689	0.026	<0.001
x5: Livestock	0.855	0.016	<0.001
Covariance: error.x2 with error.x3	0.503	0.040	<0.001
Perceived susceptibility to herpetological pathogen transmission			
x1: Chytrid transmitted to other captive amphibians	0.729	0.023	<0.001
x2: Chytrid transmitted to native amphibians	0.792	0.020	<0.001
x3: Ranavirus transmitted to other captive amphibians and reptiles	0.875	0.016	<0.001
x4: Ranavirus transmitted to native amphibians and reptiles	0.820	0.015	<0.001
x5: Ranavirus transmitted to native fish	0.772	0.018	<0.001
x6: Salmonella transmitted to other captive amphibians and reptiles	0.712	0.023	<0.001
x7: Salmonella transmitted to native amphibians and reptiles	0.809	0.019	<0.001
x8: Salmonella transmitted to pets	0.700	0.026	<0.001
x9: Salmonella transmitted to livestock	0.675	0.027	<0.001

x10: Salmonella transmitted to humans	0.633	0.029	<0.001
Covariance: error.x1 with error.x2	0.265	0.050	<0.001
Covariance: error.x1 with error.x6	0.173	0.044	<0.001
Covariance: error.x1 with error.x7	-0.265	0.055	<0.001
Covariance: error.x2 with error.x3	-0.591	0.075	<0.001
Covariance: error.x3 with error.x4	-0.625	0.082	<0.001
Covariance: error.x3 with error.x8	-0.339	0.072	<0.001
Covariance: error.x3 with error.x9	-0.504	0.072	<0.001
Covariance: error.x3 with error.x10	-0.450	0.068	<0.001
Covariance: error.x4 with error.x5	0.627	0.029	<0.001
Covariance: error.x4 with error.x9	-0.079	0.034	0.019
Covariance: error.x6 with error.x7	0.401	0.045	<0.001
Covariance: error.x8 with error.x9	0.477	0.039	<0.001
Covariance: error.x8 with error.x10	0.445	0.039	<0.001
Covariance: error.x9 with error.x10	0.294	0.046	<0.001
Sensitivity to herpetological trade risks			
x1: Other captive amphibians	0.786	0.021	<0.001
x2: Native wildlife	0.842	0.017	<0.001
x3: Pets	0.834	0.017	<0.001
x4: Livestock	0.898	0.014	<0.001
x5: Humans	0.690	0.028	<0.001
Covariance: error.x1 with error.x2	0.504	0.043	<0.001
Covariance: error.x3 with error.x5	0.342	0.045	<0.001
Covariance: error.x4 with error.x5	0.321	0.053	<0.001
Attitudes towards herpetofauna			
x1: Snakes	0.641	0.031	<0.001
x2: Lizards	0.837	0.018	<0.001
x3: Turtles/tortoises	0.596	0.032	<0.001
x4: Frogs	0.769	0.022	<0.001
x5: Toads	0.795	0.020	<0.001
x6: Salamanders/newts	0.857	0.017	<0.001
Covariance: error.x1 with error.x2	0.249	0.052	<0.001
Covariance: error.x3 with error.x4	0.157	0.040	<0.001
Covariance: error.x4 with error.x5	0.515	0.038	<0.001
Knowledge of herpetological imports			
x1: Aware that live frogs are imported for human consumption	0.678	0.034	<0.001

x2: Aware that amphibians are imported for use as fishing bait	0.736	0.033	<0.001
x3: Aware that amphibians and reptiles are imported to supply the pet industry	0.681	0.036	<0.001
Altruistic values			
x1: It is important to him/her/them that every person has equal opportunities	0.719	0.025	<0.001
x2: It is important to him/her/them to take care of those who are worse off	0.640	0.031	<0.001
x3: It is important to him/her/them that every person is treated justly	0.756	0.024	<0.001
x4: It is important to him/her/them that there is no war or conflict	0.628	0.031	<0.001
x5: It is important to him/her/them to be helpful to others	0.744	0.024	<0.001
Covariance: error.x2 with error.x3	-0.120	0.057	0.035
Biospheric values			
x1: It is important to him/her/them to prevent environmental pollution	0.780	0.021	<0.001
x2: It is important to him/her/them to protect the environment	0.830	0.018	<0.001
x3: It is important to him/her/them to respect nature	0.830	0.017	<0.001
x4: It is important to him/her/them to be in unity with nature	0.771	0.021	<0.001
Covariance: error.x1 with error.x2	0.260	0.052	<0.001
Egoistic values			
x1: It is important to him/her/them to have control over others' actions	0.740	0.030	<0.001
x2: It is important to him/her/them to have authority over others	0.878	0.031	<0.001
x3: It is important to him/her/them to be influential	0.616	0.045	<0.001
x4: It is important to him/her/them to have money and possessions	0.495	0.039	<0.001
Covariance: error.x2 with error.x3	-0.385	0.147	0.009
Hedonic values			
x1: It is important to him/her/them to have fun	0.748	0.025	<0.001
x2: It is important to him/her/them to enjoy life's pleasures	0.836	0.021	<0.001
x3: It is important to him/her/them to do things he/she/they enjoy	0.770	0.024	<0.001
Covariance: sensitivity to economic risks (agriculture) with perceived susceptibility to economic risks (agriculture)	0.409	0.046	<0.001
Covariance: sensitivity to economic risks (aquaculture) with perceived susceptibility to economic risks (aquaculture)	0.355	0.053	<0.001
Covariance: sensitivity to economic risks (frog leg market) with perceived susceptibility to economic risks (frog leg market)	0.293	0.032	<0.001
Covariance: perceived susceptibility to herpetological pathogen transmission with perceived susceptibility to economic risks	0.692	0.029	<0.001
Root mean squared error of approximation (RMSEA)	0.055		
Comparative fit index	0.851		
Akaike's information criterion (AIC)	87,396.271		

Bayesian information criterion (BIC)	89,457.907
